# Cord blood DNA methylation and cell-type composition are not significantly associated with severe preeclampsia after cell-type and clinical covariate adjustment

**DOI:** 10.1093/gigascience/giag002

**Published:** 2026-01-16

**Authors:** Xiaotong Yang, Wenting Liu, Zhixin Mao, Yuheng Du, Cameron Lassiter, Fadhl M Alakwaa, Paula A Benny, Lana X Garmire

**Affiliations:** Department of Computational Medicine and Bioinformatics, University of Michigan, Ann Arbor, MI 48109, USA; Department of Computational Medicine and Bioinformatics, University of Michigan, Ann Arbor, MI 48109, USA; Department of Computational Medicine and Bioinformatics, University of Michigan, Ann Arbor, MI 48109, USA; Department of Computational Medicine and Bioinformatics, University of Michigan, Ann Arbor, MI 48109, USA; Epidemiology, University of Hawaii Cancer Center, Honolulu, HI 96813, USA; Department of Internal Medicine, Nephrology, University of Michigan, Ann Arbor, MI 48109, USA; Department of Obstetrics, Gynecology, and Women's Health, University of Hawaii, Honolulu, HI 96826, USA; Department of Computational Medicine and Bioinformatics, University of Michigan, Ann Arbor, MI 48109, USA

**Keywords:** epigenome-wide association studies, DNA methylation, preeclampsia, cell-type deconvolution, cord blood, women’s health

## Abstract

**Background:**

Preeclampsia is a severe pregnancy complication that threatens maternal and neonatal health and well-being. Previous studies on epigenome-wide association studies (EWAS) of preeclampsia produced inconsistent results in cord blood tissues, and one possible explanation is their failure to rigorously adjust for cell proportions, gestational age, or other necessary variables.

**Methods:**

Here, we calculated the DNA methylation changes in cord blood from newborns affected by preeclampsia, using a multi-ethnic cohort from the Hawaii population (24 cases, 38 controls). We comprehensively adjusted for variables, such as maternal age, body mass index, and parity, and estimated the cell proportions. We also re-analyzed 2 previous datasets with adjustments to estimated cell proportions and conducted a pooled analysis by merging all 3 datasets together to increase the statistical power (58 cases, 71 controls). Lastly, we included idiopathic preterm (preterm delivery with no known reasons) cord blood samples (*n* = 11) to disentangle the effect of severe preeclampsia and small gestational age.

**Results:**

We showed that after adjusting cell-type proportions and patient clinical characteristics, most of the so-called statistically significant CpG methylation changes associated with severe preeclampsia disappeared in our own data, 2 public datasets, and the pooled analysis combining all 3 datasets. This result still holds after including idiopathic preterm samples in the control group. Rather, we found that gestation progression is accompanied by statistically significant proportion changes in several cell types, such as granulocytes, nucleated red blood cells (nRBCs), CD8T cells, and B cells, which contribute to most DNA methylation differences between case and control groups. Preeclampsia has interactions on cell proportion changes in granulocytes, monocytes, and nRBCs.

**Conclusions:**

In summary, our study shows that the previously reported differentially methylated patterns in cord blood are actually artifacts due to not properly adjusting for cell-type heterogeneity, gestational age, and clinical covariates. Severe preeclampsia is not associated with statistically significant DNA methylation changes but changes in cell proportion. This finding alerts to the scientific rigor needed in EWAS.

## Introduction

Preeclampsia is characterized by new-onset hypertension with proteinuria or one or more adverse conditions after 20 weeks of gestation [1]. Preeclampsia is one of the leading causes of maternal and perinatal morbidities and mortalities, affecting 2–8% of pregnancies globally and around 3.1% in the United States [2, 3]. Preeclampsia manifests as a diverse syndrome with multiple subtypes: based on blood pressure, clinical findings, and degree of proteinuria, preeclampsia can also be classified into severe preeclampsia or mild preeclampsia. Severe preeclampsia poses a greater risk to maternal and fetal health and may involve different pathways than mild preeclampsia with similar onset time [4]. Based on the onset time, preeclampsia can be divided into early-onset preeclampsia (EOPE), which occurs before 34 weeks of gestation, or late-onset preeclampsia (LOPE), which occurs after 34 weeks. If left untreated, preeclampsia may also progress into rare but life-threatening eclampsia, which causes maternal seizures. The complexity of preeclampsia poses additional challenges in understanding its root causes.

Numerous studies have been conducted to investigate the molecular mechanisms of preeclampsia [5], exploring genetic [6], epigenetic [7, 8], transcriptomic [9], lipidomic [10], and telomere length [11, 12] changes in placentas or blood of preeclampsia patients. The placenta reflects maternal–fetal interface changes, and maternal blood reveals systemic maternal physiology adaptation during pregnancy, both unable to address impacts on offspring. On the other hand, the theory of the utero origin of diseases proposes that many chronic diseases are deeply rooted in the fetal stage, the very early phase of human development [13, 14]. As the epigenome is both inheritable and prone to alteration by diseases, it is a plausible link mediating the effect of preeclampsia on offspring. Toward this, epigenome-wide association studies (EWAS) have been attempted by different studies to investigate if preeclampsia affects offspring [15–19]. However, these studies did not reach coherent conclusions on the association between preeclampsia and cord blood DNA methylation profiles.

Using an EWAS approach, Ching et al. [15] were the first to report significant global hypomethylation in cord blood from infants affected by EOPE, based on a study of 12 preeclampsia cases and 8 controls. However, in the same year, Herzog et al. [16] reported global hypermethylation in cord blood affected by EOPE compared to those in other preterm births, based on 10 EOPE samples, opposite to Ching et al. [15]. Later, Gao et al. [17] claimed statistically significant hypermethylation affecting the expression of *AVPR1a, OXTR*, and *PKCB* in preeclamptic umbilical veins, with 40 preeclampsia cases. Knihtilä et al. [19] found differentially methylated CpGs in preeclamptic cord blood associated with the cardiovascular pathway using 16 preeclampsia cases. Different from the above individual studies, Kazmi et al. [18] conducted a large-scale pooled analysis (135 preeclampsia cases) and reported 26 new statistically significant CpG sites not previously associated with preeclampsia after adjusting for estimated cell proportions and certain clinical factors. However, their results did not adjust for the gestational age, which is strongly associated with preeclampsia [18]. One possible reason for the inconsistency in previous studies may be that many of these earlier studies did not adjust for cell-type heterogeneity [15, 17, 19] or gestational age [18]. A new study with good experimental design and rigorous statistical analysis is thus necessary to assess if these variables truly contributed to the discrepancy and detect the real association between preeclampsia and methylation patterns without such interference.

Cord blood consists of many diverse cell types, each with a distinct epigenome profile [20, 21]. Thus, the varying cell types in each sample can affect the overall DNA methylation profile at the bulk level [18]. It is therefore essential to account for such heterogeneity, to improve the robustness and validity, as well as avoid biased conclusions of EWAS biomarker detection. Particularly, the analysis needs to be adjusted for cell proportions. Moreover, if essential clinical data such as gestational age are available (as they should be), then the analysis needs to be adjusted for the important clinical variables as well. In this study, we specifically address these issues to seek potential epigenomic and cellular markers in cord blood associated with severe preeclampsia.

## Materials and methods

### The Hawaii Biorepository cohort

The umbilical cord whole blood DNA samples were obtained from the Hawaii Biorepository (HiBR). HiBR collected placenta, maternal, and cord blood samples from deliveries at Kapiolani Women and Children’s Hospital from 2006 to 2013. It is one of the largest research tissue repositories in the Pacific region, containing specimens from more than 9,250 mother–child pairs at the time of sample collection. The repository obtains informed consent from women postpartum to donate their placenta, umbilical cord, and excess cord and maternal blood (routinely collected for care purposes). Umbilical cord samples were collected immediately after delivery. Severe preeclampsia was characterized by obstetricians and gynecologists (OBGYNs) at Kapiolani Medical Center: among patients with preeclampsia (blood pressure ≥140/90 mmHg with proteinuria), those who additionally present with (i) severe-range hypertension (blood pressure ≥160/110 mmHg), (ii) severe proteinuria (≥5 g in a 24-hour urine specimen or ≥3+ on 2 random urine samples collected at least 4 hours apart), or (iii) evidence of organ dysfunction [22].

This is part of a parent study to investigate the multi-omics biomarkers for severe preeclampsia and how the mothers and their female offspring are protected against breast cancers later in life, using the previously biobanked samples in the HiBR [10, 12]. The parental study includes women with severe preeclampsia who delivered singletons and were matched 1-to-1 by healthy preeclampsia-free deliveries based on maternal age, ethnicity, and pre-pregnancy body mass index (BMI). For this nested cord blood study, we included those who delivered female babies and had cord blood samples remaining in the HiBR. We evaluated sample integrity, purity, and concentration on the NanoDrop and removed samples of low quality, with 260/280 (~1.8) and 260/230 (~2.0) ratios serving as standard-quality thresholds. Variables such as maternal age, ethnicity, pre-pregnancy BMI, gestational age, parity, and self-reported smoking status during pregnancy were recorded. Ethnicity includes European ancestry, Asian, and Pacific Islander ancestry. Patients with unknown ethnicity were excluded. Smoking is the self-reported smoking status (yes/no) during pregnancy. This cord blood cohort contains 24 women with severe preeclampsia and 38 preeclampsia-free healthy controls. The demographic and clinical information of the patients was collected and analyzed to identify any potential associations with DNA methylation. Data usage was approved by IRB #CHS23976.

### Additional cohorts

To validate the observations in the HiBR cohort, we searched PubMed for published studies of preeclampsia and cord blood (including cord blood mononuclear cells) DNA methylation and included all public datasets identified [15, 16, 23]. We were able to obtain DNA methylome Illumina 450k data from the following studies: (i) Ching et al. [15] with 12 preeclampsia cases and 8 controls, along with 6 clinical variables available, including maternal age, maternal BMI, maternal ethnicity, gestational age, infant gender, and infant birth weight. (ii) Herzog et al. [16] with 23 severe preeclampsia samples (including 10 EOPE and 13 LOPE samples) and 25 control samples, but no clinical variables available (GSE103253). (iii) Cord blood leukocytes from Kashima et al. [23] with 20 preeclampsia cases and 90 controls, as well as 7 clinical variables available, including maternal age, maternal BMI, maternal smoking before pregnancy, parity, gestational age, infant gender, and delivery (GSE110828). We were unable to obtain data from the study by Kazmi et al. [18] as the data are not publicly available. We obtained the raw data from Ching et al. [15]. The data were filtered, normalized, and then corrected for batch effects by slide and array, using the R package ”ChAMP.” For the Herzog et al. [16] and Kashima et al. [23] datasets, we directly used the processed DNA methylation data (β matrix) deposited to the Gene Expression Omnibus (GEO).

We also included cord blood samples from Fernando et al. [24] (GSE66459). This dataset includes 11 idiopathic preterm birth samples and 11 full-term samples. Idiopathic preterm births are preterm deliveries with no known reasons, triggered by spontaneous preterm labor (PtLb) with intact membranes or preterm premature rupture of membranes (PPROMs). All samples from this cohort are preeclampsia-free. We directly used the normalized β matrix deposited to GEO.

### Sample preparation

Umbilical cord blood samples were collected immediately after delivery. To prepare for DNA extraction, we first added 3 volumes of RBC Lysis Solution (Qiagen) to 1 volume of clotted blood, which was then vortexed and incubated on a shaker for 15 minutes at room temperature. The sample was then centrifuged to pellet white blood cells and clot particulates, and the supernatant was carefully poured into a waste bucket. The pellet was resuspended in an additional volume of RBC Lysis Solution and incubated again for 15 minutes. After another centrifugation step, the supernatant was carefully removed, leaving behind 200 µL of residual liquid. The pellet was then vigorously resuspended in the residual liquid before being combined with a master mix of Cell Lysis and Proteinase K Solution (Qiagen). The mixture was vortexed and incubated at 55°C until homogeneous, with intermittent vortexing to facilitate digestion. Once homogeneous, the samples were subjected to DNA purification on the Autopure Machine (Qiagen) following the manufacturer’s instructions.

### DNA extraction and methylation profiling

DNA was extracted from prepared cord blood samples by HiBR using the AllPrep DNA/RNA/Protein Mini Kit (Qiagen) according to the manufacturer’s instructions. We obtained preextracted genomic DNA of whole cord blood samples from the HiBR and conducted DNA Illumina EPIC Beadchip assays through the University of Hawaii Cancer Center Genomics Core. Case and control samples were interleaved on the plate to ensure the sample group was independent of batch effects. We used the EZ DNA Methylation kit for the bisulfite conversion step (Zymo Research).

### DNA methylation data preprocessing and quality control

We used the R package “ChAMP” for data preprocessing (Supplementary Fig. S1). We first filtered probes using the following criteria sequentially: (i) removing probes with a detection *P*-value above 0.01 on any sample (7,941 probes), (ii) removing probes with a bead count <3 in at least 5% of samples (27,731 probes), (iii) removing non-CG(ch) labeled probes (2,673 probes), and (iv) removing probes that align to multiple locations (25,194 probes). We used the newer annotation list by Zhou et al. [25] to identify these probes. During the quality control step, we removed 1 control sample with a distinct β density distribution (Supplementary Fig. S2A, B). We normalized the remaining samples using BMIQ methods [26] embedded in “ChAMP.” The preprocessed data matrix contains 62 samples and 819,325 probes. We converted the original methylation intensity (β) to M-values using “beta2m” function from the “lumi” package to reduce heteroskedasticity [27], where M-values are defined as the log2 ratio of the β value of each probe.

### Cell-type deconvolution in umbilical cord whole blood

Bulk-level DNA in umbilical cord whole blood (CB) includes at least 7 most common blood cell types: granulocytes, B lymphocytes (B cells), CD4^+^ lymphocytes (CD4T), cytotoxic T lymphocytes (CD8T), monocytes, natural killer (NK) cells, and nucleated red blood cells (nRBCs). Each sample may have different compositions of the cell types above, thus needing deconvolution. We adopted Houseman’s constrained projection (CP) algorithm [28] and a combined cord blood cell-type reference as recommended by Gervin et al. [21]. This combined reference includes 263 cord blood cell-type signatures from 4 previous large studies [29–32] and is used in many previous cord blood cell-type estimation studies [33, 34]. We applied this deconvolution approach consistently across all datasets, both in-house and public. We adjusted the estimated cell-type proportions in all the differential analyses.

### Clinical variables and source of variance analysis

We retrieved 6 commonly adjusted clinical variables in cord blood EWAS study from the biobank, including maternal age, ethnicity (including Asian, European ancestry, and Pacific Islander), parity, pre-pregnancy BMI, delivery gestational age, and smoking status [18]. We imputed 3 samples (including 1 severe preeclampsia and 2 controls) with missing BMI using the mean values of each sample group. To assess their relative contribution to methylation variation, we performed the source of variance (SOV) analysis on these clinical variables and previously estimated sample cell proportions as done before [10, 35, 36]. Specifically, we regressed each CpG site on all clinical variables and applied 2-way analysis of variance (ANOVA) to each regression. Next, we averaged the *F*-statistics for each variable across CpG sites. Importantly, we only used SOV to confirm the importance of these variables, not to select; all variables were retained in the downstream regression models. A directed acyclic graph (DAG) illustrating the assumed causal relationships among exposure, outcome, and covariates is provided in the Supplementary Fig. S3. Note that although gestational age and cell proportions are mediators on the causal pathway between preeclampsia (PE) and DNA methylation, in EWAS studies, it is still common practice to adjust for them, because researchers are interested in the direct effect of a disease on DNA methylation, which is more biologically meaningful [18, 19].

### CpG-level EWAS

We calculated the differentially methylated probes (DMPs) between severe preeclampsia cases and controls by fitting linear regression with empirical Bayes-moderated statistics on each probe. The *P*-value of preeclampsia was adjusted with Benjamini–Hochberg (BH) adjustment (type I error threshold of 0.05). We included study participants’ gestational age, BMI, parity status, ethnicity, and methylation-derived cell compositions in the linear model and compared the result without adjusting for these variables using “limma” package [37]. We included batch variables (Slide and Array) in the regression as dummy variables to avoid the batch effect. Because estimated cell-type proportions are compositional (sum to one), we transformed the vector of proportions using the isometric log-ratio (ILR) transform with a sequential binary partition to obtain an orthonormal set of k − 1 coordinates from R package “compositions.” The resulting ILR coordinates were included as covariates in the EWAS regression to adjust for cell composition without inducing collinearity. We defined hypermethylated CpGs as statistically significant CpGs with positive log2-transformed fold change (logFC) and hypomethylated CpGs as statistically significant CpGs with negative logFC, respectively. We calculated the empirical null inflation factor (λ) for each EWAS result as estimated by the “BACON” package [38], which measures how much test statistics deviate from the expected null, indicating possible bias or confounding. An empirical null-based bias and inflation adjustment (“BACON” method) was applied when λ exceeded 1.2, a commonly accepted threshold for substantial inflation. We used volcano plots to illustrate the global DNA methylation changes between the cases and controls.

We also explored the potential cell-specific DNA methylation changes in preeclampsia using the cellDMC function from the Bioconductor package “EpiDISH.”

### Pooled analysis using 3 datasets

We also combined the 3 cohorts (in-house, Ching et al. [15], Herzog et al. [16]) and conducted a pooled analysis to improve the test power and produce more robust results. We did not include data from Kashimi et al. [23] because their data were extracted from cord blood mononuclear cells (CBMCs) instead of the whole blood.

To reduce non-biological variation introduced by technical factors such as different array runs, slides, and laboratories, we harmonized the datasets following this pipeline: (i) we filtered the raw in-house EPIC data and raw 450k data from Ching et al. [15] individually, then merged them based on overlapping CpG sites and normalized the dataset; (ii) we combined the merged and normalized dataset with the normalized β matrix from Herzog et al. [16], downloaded directly from GEO; and (iii) we applied the ComBat function to harmonize the datasets while preserving sample group information. We then calculated the differentially methylated probes between severe preeclampsia cases and controls using limma and plotted the volcano plot on the harmonized data. We estimated the cell proportions of the merged data using Houseman’s CP method and the cell-type reference by Gervin et al. [21]. Lastly, we calculated differentially methylated probes again, with adjustment to the estimated cell types, and plotted another volcano plot.

### Including idiopathic preterm samples to decouple preeclampsia and small gestational age

Many preeclampsia patients are delivered preterm to avoid severe maternal complications, resulting in an inevitable positive correlation of preeclampsia status and smaller gestational age. To decouple preeclampsia and gestational age, we include another study, Fernando et al. [24] (GSE66459). The dataset contains DNA methylation data of 11 idiopathic preterm and 11 term samples processed with the Illumina 450K Human methylation bead chip array. We merged our in-house data with data from Fernando et al. [24] by common CpGs, then computed the differentially methylated probes with adjustment of gestational age and infant sex and estimated the cell proportions again using the limma package. Similarly, we examined and corrected for potential bias and inflation using the empirical null distribution from the “BACON” R package [38].

### Differential methylated regions

To identify the differentially methylated regions (DMRs), we used the bumphunter method from the R package bumphunter. We used the “clustermaker” function to identify clusters, with default parameters; the “bootstrap” method to generate null candidate regions; and 0.2 and −0.2 as the upper and lower bounds of the candidate regions (for more details of parameter choice, see “5.3–DMR.R” in the code). The result was adjusted for the same clinical variables and cell-type proportions as the CpG-level differential analysis. We used the Family-Wise Error Rate (FWER) method to adjust the *P*-values of each DMR and used an adjusted *P-*value of <0.05 as the cutoff for statistically significant DMRs.

### Gene-level EWAS

We further examined the methylation signal difference between severe preeclampsia and controls at gene and pathway levels. We annotated the CpGs [39], selected those located on the promoter region, and aggregated the methylation signals of all CpGs within a gene promoter by taking the geometric mean. We then compared the aggregated methylation signals between severe preeclampsia cases and controls, using linear regression with empirical Bayes-moderated statistics. Additionally, we looked for pathways associated with promoter region methylation differences using the R package pathifier [40]. The *Pathifier* algorithm calculates a pathway deregulation score (PDS) for each sample and each pathway. We compared the pathway PDS scores in case and control samples adjusted for the same variables as the probe-level analysis; the resulting *P*-values were adjusted with the Benjamini–Hochberg false discovery rate (threshold *P*-values = 0.05).

## Results

### Overview of study design and cohort characteristics

**Figure 1:**
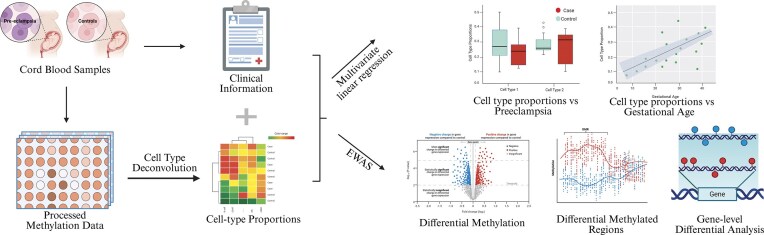
Study overview and experiment design. The entire data analysis procedure is outlined in this workflow, which incorporates methods that account for clinical variables and cell-type heterogeneity. Created in BioRender. Garmire, L. (2026) https://BioRender.com/3ujp26c

The overview of the study design is illustrated in Fig. 1. We analyzed whole cord blood from 24 severe preeclampsia cases and 39 controls collected at the Hawaii Biorepository (2006–2013). Maternal characteristics were similar between the preeclampsia and control groups, except cases had significantly earlier (*P* = 3.66 × 10^−6^) gestational age at delivery (Table 1). Genomic DNA was assayed on the Illumina EPIC BeadChip, and data were preprocessed with the R package *ChAMP* (Supplementary Fig. S2), leaving 62 samples and 819,325 probes for analysis.

**Table 1:** Patient characteristics

	Preeclampsia cases	Controls	
	(*n* = 24)	(*n* = 38)	
Variables	Mean (SD)	Mean (SD)	*P-*value
Maternal age (years)	28.75 (5.88)	27.24 (6.35)	0.40
Parity	1.54 (1.41)	1.57 (1.78)	0.77
BMI	32.24 (9.38)	27.75 (9.20)	0.07
Smoker (*n*)	6	8	0.96
Gestational age (weeks)	35.58 (2.90)	39.16 (0.92)	2.485e-07
**Ethnicity (*n*)**			0.60
Asian	12	21	–
European Ancestry	3	7	–
Pacific Islander	9	10	–

Numeric variables are compared with the Wilcoxon test. Categorical variables are compared with the chi-square test.

### Associations between cord blood cell types and severe preeclampsia

**Figure 2:**
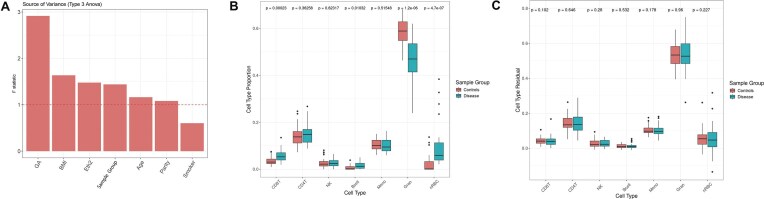
Cell types in samples. (A) The SOV analysis of cell-type composition from patient characteristics. Major contributors to DNA methylation variation were identified as the factors (x-axis) with an F-mean value greater than 1. (B) Side-by-side boxplots displaying cell-type proportions in preeclampsia cases versus controls before adjusting for clinical variables. An asterisk (*) is used to indicate a statistically significant difference by using multiple linear regression (MLR) between the case and control groups (*P* < 0.05), while “ns” is used to indicate a not statistically significant difference. (C) Side-by-side boxplots displaying cell-type proportions in preeclampsia cases versus controls with *P-*values from multiple linear regression of cell proportions on preeclampsia and all major factors for methylation variation in (A).

To learn the association between severe preeclampsia and cord blood cell composition, we performed cell-type deconvolution using the cord blood cell-type reference as recommended by Gervin et al. [21] and Houseman’s CP deconvolution algorithm [28]. We assessed the important cell-type compositions and clinical variables using the SOV analysis and ranked them by *F*-statistics. As shown in Fig. 2A, in addition to severe preeclampsia, gestational age, maternal BMI, and ethnicity also had statistically significant contributions to cell-type heterogeneity. Gestational age (*F*-statistic = 9.30) and maternal BMI (*F*-statistic = 3.10) rank higher than severe preeclampsia status (*F*-statistic = 2.41), with gestational age being the predominant influencing factor. We then compared the cell proportions between case and control groups (Fig. 2B): granulocyte proportions are statistically significantly lower (*t* = −0.11, *P* = 1.20e-06) in the severe preeclampsia group compared to the control group, while B cell (*t* = 6.93e-3, *P* = 0.01), nRBC (*t* = 0.08, *P* = 4.7e-7), and CD8T cell (*t* = 0.02, *P* = 2.3e-3) proportions seem statistically significantly higher in cases. However, the apparent differences in cell proportions in cases versus controls could very well be due to other reasons (e.g., gestational age) rather than the severe preeclampsia directly. With such caution, we calculated the association between cell proportions and severe preeclampsia again with linear regression. This time, we included gestational age, maternal age, ethnicity, BMI, and smoking status as covariate factors. For comparison, we plotted the cell proportions in severe preeclampsia versus controls and reported the *P-*values of preeclampsia from linear regression with other clinical covariates (Fig. 2C). The previously observed differences in cell proportions now disappear. The detailed linear regression results of cell proportions on clinical data can be found in Supplementary Table S1. We confirmed this finding and showed that the effect of PE on cell proportions is mediated by gestational age, using a mediation analysis of gestational age on the effect of PE on each cell type (Supplementary Fig. S4). In all, these results show that certain cord blood cell proportions vary among newborns, and it is important to adjust for cell-type heterogeneity before interrogating the association with severe preeclampsia to study the direct effect of preeclampsia on DNA methylation change.

### Lack of association between cord blood DNA methylation and severe preeclampsia

**Figure 3:**
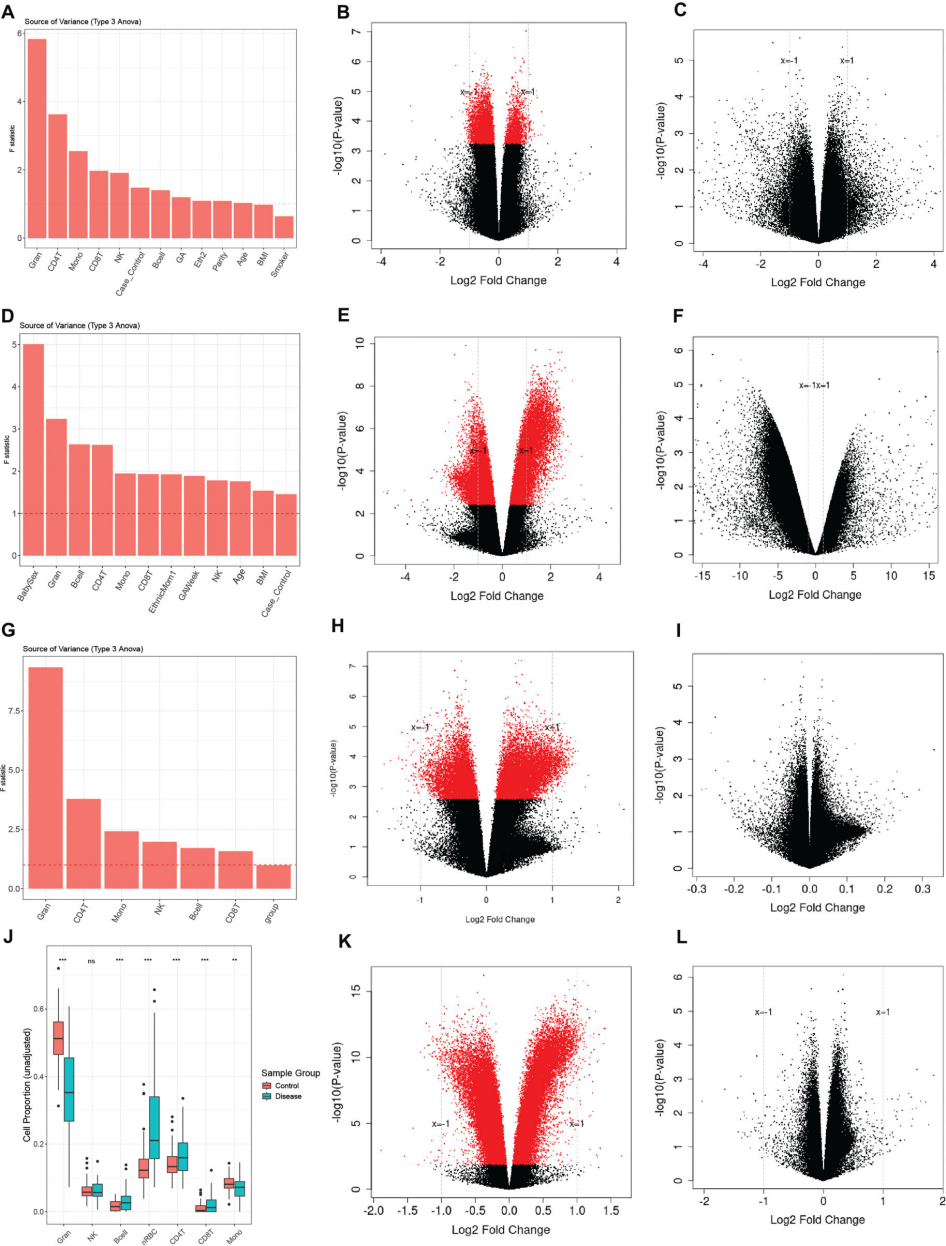
Preeclampsia is not associated with statistically significant changes in cord blood DNA methylation, after clinical variables, gestational age, and cell proportion adjustment. Results were generated using data from the HiBR cohort (A–C), Ching et al. [15] (D–F), Herzog et al. [16] (G–I), and pooled analysis combining the 3 cohorts (J–L). (A, D, G) The SOV analyses were conducted on cell types and clinical variables whenever available, to verify the proportion of DNA methylation variance attributable to these factors. (B, E, H, K) The volcano plot of the differential methylation analysis results without adjustment. The x-axis represents log fold change between severe preeclampsia and controls; the y-axis indicates negative log-transformed *P*-values after Benjamini–Hochberg (BH) adjustment. The red dots are differentially methylated probes (DMPs) associated with severe preeclampsia after BH adjustment, whereas the black dots represent non–statistically significant probes. (C, F, I, L) The volcano plot after adjusting for clinical variables, gestational age, and estimated cell proportions. (J) The cell proportion distributions in the combined 3 datasets, separated by sample groups. B cell: B-lymphocyte; Gran: granulocyte; Mono: monocytes; NK: natural killer cell; nRBC: nucleated red blood cell, CD4T.

Considering that most previous cord blood EWAS studies overlooked the adjustment for cell types or other clinical covariates (such as gestational age) within their samples, we further investigated the impact of these factors on the differential methylation analysis. We conducted the SOV analysis of the DNA methylation matrix on cell-type proportion and clinical covariates. Strikingly, all cell-type composition variables show the strongest and most dominant explanatory power of variation in the methylation data (Fig. 3A), ranking even higher than the severe preeclampsia condition itself. After the cell proportions, severe preeclampsia case/control, gestational age, maternal age, parity, and ethnicity also have larger *F*-statistics than the error term (*F*-statistics = 1), in descending order. Therefore, in the downstream analysis of differentially methylated CpGs, we adjusted for these variables.

As a comparison, we first conducted a differential methylation analysis on severe preeclampsia without adjusting for any clinical covariate or cell-type proportions. The analysis reveals a global hypomethylation pattern (Fig. 3B). We identified 229,730 differentially methylated CpGs with adjusted *P-*values less than 0.05. Among these CpGs, 184,102 exhibited hypomethylation, while 45,628 displayed hypermethylation. However, when we redid the differential methylation analysis after adjusting for cell-type heterogeneity and patient characteristics, all the CpGs differentially methylated above were no longer statistically significant (Fig. 3C). We also examined how the directions and magnitude of significant CpGs identified before adjustment changed after adjustment. Specifically, we used *t*-statistics from the limma regression as a measure of effect size. The magnitude of the effects substantially decreased after adjusting for gestational age and estimated cell-type proportions, and approximately 37% of CpGs reversed their direction of association (Supplementary Fig. S5). This large direction change and magnitude reduction support the notion that the apparent methylation differences observed before adjustment were largely attributable to variation in cell composition rather than true disease-related methylation changes.

We also explored cell-specific differential methylation with the “cellDMC” algorithm and found no significant association.

Additionally, we conducted DMR analysis, which also suggests no statistically significant association with severe preeclampsia when adjusted for the same variables of cell types and clinical contributors, as done in Fig. 3C (Supplementary Table S2). We extended the differential methylation analysis to the gene level by aggregating the CpGs located on gene promoters as the representation of promoter-level methylation (see Materials and Methods). Before adjusting for clinical covariates, gestational age, and cell proportions, we detected 4,767 differentially methylated genes. However, upon adjusting for both clinical covariates and cell types, none of the genes exhibited statistical significance. Similarly, we conducted a differential methylation analysis at the pathway level, employing the Pathifier algorithm (see Materials and Methods). Before the adjustment, we detected 200 statistically significant pathways; after adjustment, none of the pathways remained statistically significant.

To confirm this surprising finding that contradicts all previous EWAS studies on cord blood samples associated with preeclampsia, we re-analyzed all other available public whole cord blood (or CBMC) DNA methylation datasets associated with preeclampsia samples from Ching et al. [15], Herzog et al. [16], and Kashima et al. [23]. We estimated the cell-type proportions the same way, using Houseman’s CP algorithm and the new combined cord blood reference recommended by Gervin et al. [21]. We conducted the SOV analysis by considering cell proportions in both studies and clinical variables whenever available (for Ching et al. [15]). SOV shows that cell-type proportions, gestational age, maternal age, and preeclampsia are important variables, as they explain more variance than residual noise (*F*-statistics > 1) (Fig. 3D, G). For the dataset of Ching et al. [15], we used the original analysis pipeline that did not consider any adjustment and reproduced the differential methylation results earlier, which reported 68,458 statistically significant CpGs (Fig. 3E). However, once we adjust for the gestational age, cell proportions, and other clinical covariates, there are no longer statistically significant CpGs (Fig. 3F). For the newborn umbilical cord blood dataset of Herzog et al. [16], we conducted a differential methylation analysis as well. Without adjustment, we obtained 24,597 statistically significant CpGs (Fig. 3H). Again, once we adjusted the cell-type proportions (all 7 major whole cord blood cell types: monocytes, CD4T cells, natural killer, granulocytes, nRBCs, B cells, CD8T cells), there is no statistically significant CpG remaining (Fig. 3I). For Kashima et al. [23] data, we found no statistically significant CpGs even before cell-type adjustment (Supplementary Fig. S6). Thus, using all 3 other available cord blood datasets, we confirm that there indeed is a lack of association between cord blood DNA methylation and severe preeclampsia.

To increase the statistical power, we further conducted a pooled analysis by combining our in-house data, as well as data from Ching et al. [15] and Herzog et al. [16]. The 3 datasets were processed and harmonized as described in the Materials and Methods section. Again, the differential methylation result of combined data shows that many statistically significant CpGs are associated with preeclampsia before adjustment (Fig. 3I) but are not statistically significant after adjusting for cell proportions (Fig. 3L). Additionally, to best decouple preeclampsia and early gestational age, we included the idiopathic preterm samples from Fernando et al. [24]. The dataset has 11 preterm and 11 full-term samples whose cord blood DNA was processed with Illumina 450K beadchips. We merged them with our HiBR data and computed the differentially methylated CpGs (see Materials and Methods). Consistent with previous conclusions, we found no statistically significant difference between preeclampsia cases and non-preeclampsia controls, after adjusting for cell proportion, infant sex, and gestational age (Supplementary Fig. S7). We thus conclude that there is a lack of association between cord blood DNA methylation changes and severe preeclampsia.

### Association between cord blood cell type and gestational age

**Figure 4:**
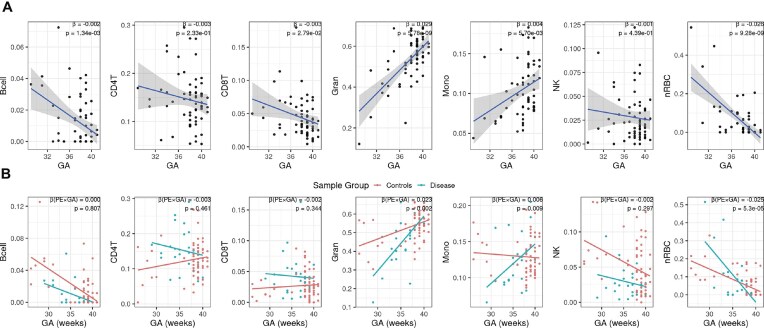
Cell-type proportions in relation to the gestational age. (A) Scatterplots depict the proportions of each cell type in cord blood from all samples, along with gestational age. The reported β and *P-*values measure the relationship between gestational age and each cell type, with a threshold of *P* < 0.05. (B) Scatterplot of estimated cell proportions from merged HiBR data and those from Fernando et al. [24], by gestational age. β and *P*-values refer to the coefficient and *P-*values of the interaction between preeclampsia and gestational age in each cell type.

Our earlier analysis shows that estimated cell proportions in cord blood are mostly correlated with gestational age (Fig. 2C). We thus conducted a more in-depth analysis. The most noticeable correlation comes from granulocytes, whose proportions increase from around 25% in week 32 to over 50% in week 40, with *P* < 2.11e-11 (Fig. 4A). The proportions of monocytes also statistically significantly increase as gestation progresses (β = 0.004, P = 5.70e-3). On the contrary, B cells, CD8T cells, and nRBCs statistically significantly decrease along the gestation (β = −0.002, *P* = 1.34e-3; β = −0.003, *P* = 2.79e-2; β = −0.026, *P* = 9.28e-9). We also plotted the trends of cell-type proportions related to gestational age per sample group by merging our HiBR data and data from Fernando et al. [24] (Fig. 4B). These trends of cell proportions are mostly the same in the case and control groups, except for monocytes, granulocytes, and nRBCs, which show a potential interaction between preeclampsia and gestational age.

**Figure 5:**
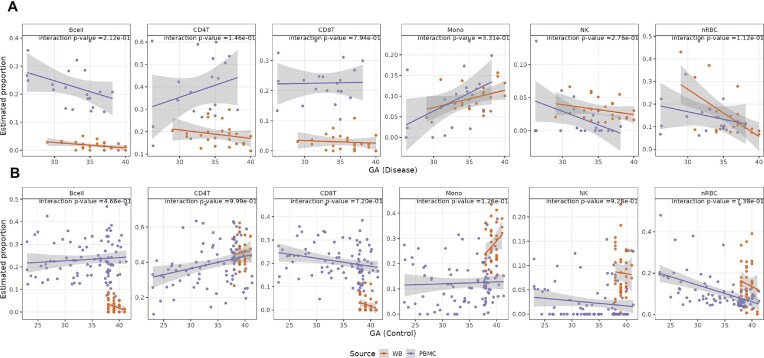
Cell proportions in relation to gestational age are coherent across 2 different datasets. The scatterplots compare the cell-type proportions with gestational age in our data and Kashima et al. [23]. Plots (A) displays the comparisons within preeclampsia case samples for both studies, whereas plots (B) display the comparisons within control samples for both studies. The purple line in each plot represents the fitted cell proportions in our whole cord blood samples, while the orange line represents the fitted cell proportions in CBMC cord blood samples from Kashima et al. [23]. We test whether the cell proportion changes in the 2 datasets, using CBMC versus the whole blood (WB), are statistically significantly different by linearly regressing cell proportions over gestational age, datasets, and the interaction between the datasets and gestational age. We report the *P-*values of such interaction terms. A not statistically significant *P*-value indicates that the cell proportion trends are not statistically different in the 2 datasets.

Furthermore, we validated the trends of cell-type proportions through gestational age using another public CBMC Illumina HumanMethylation450 BeadChip dataset (GSE110828), which comprises 20 preeclampsia cases and 90 non-preeclampsia controls [23]. Both the case and control groups include large percentages of preterm samples, with the delivery gestational ages ranging from 26.14 to 38.14 weeks in cases and 23.00 to 41.29 weeks in controls. We deconvoluted the CBMC cell types using the same combined cord blood reference. To ensure comparability of cell proportions between CBMC and whole blood, we eliminated granulocytes, which are unique to whole blood, and recalibrated the weights of the remaining cell types to sum to one. In both preeclampsia (Fig. 5A) and control samples (Fig. 5B), we observed the same increasing trend for monocytes and the same decreasing trend for CD8T cells, nRBC cells, and B cells. For NK cells, while both cohorts show consistent trends of decrease in the control samples with gestational age, the trend in preeclampsia samples is not conclusive, possibly related to the small sample size (*n* = 20) in the other CBMC cohort. Further, to test whether the cell proportion trends in relation with gestational age are consistent between the 2 datasets, we performed linear regression of each cell proportion (y-variables), over gestational age, CBMC versus whole blood dataset stratification, and the interaction terms between datasets and gestational age, with adjustment of other variables for preeclampsia cases and controls separately. None of the gestational age (GA) interaction terms were statistically significant (Fig. 5A). A nonsignificant interaction *P*-value indicates that the GA–cell-proportion trends do not differ statistically between the case and control groups. This shows that the association between gestational age and cell proportions is consistent and independent of datasets.

## Discussion

In this study, we show that there is a lack of association between severe preeclampsia and offspring’s cord blood DNA methylation changes after adjusting for cord blood cell proportions and clinical variables from multiple cohorts. Interestingly, aligning with our observation, recent work by Campbell et al. [41] also showed that substantial placental cellular heterogeneity in preeclampsia contributes to previously observed bulk gene expression differences. Besides the absence of CpG changes associated with severe preeclampsia, we also observed that the cell type proportions are not significantly different between cases and controls, after adjusting for clinical covariates. Together, these observations highlight the importance of rigorous adjustment for developmental and cellular heterogeneity when interpreting EWAS results in pregnancy-related conditions.

Furthermore, we noticed generally consistent associations between cell-type compositions in cord blood and gestational age in 2 independent cohorts, regardless of the existence of severe preeclampsia conditions. Granulocyte proportion showed the strongest quantitative changes along gestational age, agreeing with the previous findings that granulocytes in the fetus increase drastically in the last trimester of pregnancy [42, 43]. On the contrary, the estimated proportion of nRBCs in our study decreases drastically as gestational age increases, also consistent with previous findings [44–46]. Previously elevated nRBCs was also found in preterm infants and infants with lower birth weight [47], providing additional supporting evidence to our finding. Braid et al. [42] also reported decreases in B cells in cord blood using methylation-derived cell proportion. One explanation is that the large increase in granulocytes in late gestation makes the proportions of other cell types smaller, not necessarily the absolute value. Another possibility is that preterm infants are experiencing higher inflammation levels, which leads to a higher B cell proportion [42]. Taken together, these findings probe into the dynamic nature of cell-type composition in cord blood during gestation, confirming the importance of cell-type adjustment in methylation analysis.

Cell-type heterogeneity is one of the most influential factors affecting EWAS results. Over the years, various cell-type estimation methods for bulk-level epigenetic data have been developed [28, 48], making the assessment of cell proportions effects possible. In 2013, Liu et al. [49] first reported a large reduction in differentially methylated probes related to rheumatoid arthritis after adjusting for cell-type composition in whole blood. Some later studies confirmed the issue with cell-type heterogeneity on EWAS research in other tissues, such as breast tissue, saliva, and placenta tissue [33, 41, 50–52], emphasizing the importance of adjusting for cell-type heterogeneity in the EWAS of preeclampsia studied here. Kazmi et al. [18] conducted a large pooled analysis on the association of preeclampsia and newborn DNA methylation and detected a small number of 26 statistically significantly associated CpGs. However, they did not adjust for gestational age, which is strongly associated with both preeclampsia and DNA methylation, and therefore should be included as a key covariate [18]. Additionally, we show that gestational age is associated with cell proportion change, as reported before [53]. More importantly, although several previous studies aimed to identify preeclampsia-related epigenetic biomarkers using cord blood samples [15–18], the importance of adjusting for cell-type heterogeneity was mostly (3 of 4) overlooked. Using as many as 4 different cohorts, our investigation here shows that ignoring the cell-type heterogeneity and gestational age may have contributed to biased EWAS associations with preeclampsia, as done previously by multiple studies.

Some caveats are worth mentioning for this study. First, all the cell types in the blood are computationally inferred, rather than experimentally measured. In theory, technology such as flow cytometry is likely more powerful for direct applications in EWAS or preeclampsia. Now, cord blood references only contain 6 to 7 cell types; a reference with finer grid cell types can yield more insights. However, for retrospective studies such as this one, where only whole blood DNA was available, or for historically archived bulk DNA methylation data, computational deconvolution is the only viable option. Also, infections such as chorioamnionitis, which unfortunately is not part of the collected clinical variables here, may also influence the association between gestational age and cell proportions by triggering a surge of neutrophils. However, given the small proportions of chorioamnionitis, this might not be a major issue. In addition to the variables we already considered, lifestyle factors such as diet and physical activity may also influence DNA methylation patterns and should be included in future works. Genetic variation (e.g., polymorphisms, relatedness, structural variants) may also contribute to DNA methylation differences [54], though our attempt to remove single-nucleotide polymorphism overlapping CpGs should mitigate these issues to some degree. Future follow-up histopathological investigation into the maternal decidua may examine vascular malperfusion, but it is beyond the scope of this study. Lastly, the cord blood samples studied here are modest in size (*n* = 62). The subsequent pooled analysis in Fig. 3L has a total of 129 (preeclampsia = 58) cord blood samples, smaller than those in Kazmi et al. [18]. We could not exploit the data in Kazmi et al. [18] due to the lack of open access. It will be highly interesting to combine these data together for reanalysis with statistical rigor in the future. We also aim to emphasize the importance of variable adjustment in EWASs of pregnancy-related diseases, as drastic changes in cell proportions during pregnancy can strongly influence DNA methylation patterns.

## Conclusion

In summary, we could not find the evidence for statistically significant CpG methylation changes in EWAS analysis in association with severe preeclampsia, after adjusting for cell-type heterogeneity and clinical variables such as gestational age. Additionally, many cell-type proportions change drastically as pregnancy progresses.

## Availability of source code and requirements

Project name: CB_DNAm_PE

Project homepage: https://github.com/lanagarmire/CB_DNAm_PE.

License: GPL-3.0 license

System Requirements

Operating system: Windows, macOS, Linux

Programming language: R 4.1.2 [55].

Package management: “ChAMP” (version 2.24.0) for data preparation [56], “limma” for differential methylation analysis, “EpiDISH” [57] (version 2.10.0) for Houseman’s CP cell-type deconvolution algorithm, Bioconductor package “b9.bioc.FlowSorted.CordBloodCombined.450k” for cord blood DNA methylation reference, and “IlluminaHumanMethylationEPICanno.ilm10b4.hg19” for data annotation

## Supplementary Material

giag002_Supplementary_material

giag002_Authors_Response_To_Reviewer_Comments_original_submission

giag002_GIGA-D-25-00219_original_submission

giag002_GIGA-D-25-00219_Revision_1

giag002_Reviewer_1_Report_original_submission Tibor Varga -- 7/14/2025

giag002_Reviewer_2_Report_original_submission Karolis Koncevicius -- 7/15/2025

giag002_Reviewer_2_Report_Revision_1 Karolis Koncevicius -- 11/27/2025

giag002_Reviewer_3_Report_original_submission Mariona Bustamante -- 7/16/2025

giag002_Reviewer_3_Report_revision_1 Mariona Bustamante -- 11/15/2025

## Data Availability

In-house DNA methylation data have been deposited in the Gene Expression Omnibus (GEO) under accession number GSE299721. Reused public datasets are also available from GEO, including those from Herzog et al. [16] (GSE103253), Kashima et al. [23] (GSE110828), and Fernando et al. [24] (GSE66459), as well as from Ching et al. [15]. All additional supporting data are available in the *GigaScience* repository, GigaDB [58].

## Additional Files

**Supplementary Fig. S1**. Data processing workflow. The complete data preprocessing procedures consisted of filtration, imputation of missing values, quality control checks, normalization, and conversion of β values to M-values. BMIQ: beta-mixture quantile normalization. Created in BioRender. Garmire, L. (2026) https://BioRender.com/lyryqsk

**Supplementary Fig. S2**. Data quality control. (A, B) Density plots before and after BMIQ normalization and the removal of 1 control sample with a distinct β density distribution. Color represents significance level.

**Supplementary Fig. S3**. Directed acyclic graph (DAG) of assumed causal relationships. Preeclampsia (PE) is the exposure, and DNA methylation changes (DNAm) are the outcome. Pre-pregnancy body mass index (BMI), maternal age (MAge), ethnicity (Eth), smoking during pregnancy (smoke), and parity are confounders that are associated with both PE and DNA methylation. Gestational age at delivery (GA) and estimated cell proportions (CellProp) are mediators between PE and DNA methylation, but most cord blood EWAS still adjust for them because researchers are more interested in the direct effect of PE on DNA methylation.

**Supplementary Fig. S4**. Mediation analysis of gestational age between PE and estimated cell proportion. (A) Illustration of the mediation effect of gestational age (GA) on the effect of preeclampsia (PE) on cell proportion. (B) The effect size of the direct effect, indirect effect, and total effect of each cell proportion. The effects were calculated by regressing each cell proportion on preeclampsia and other clinical confounders: the direct effect is the coefficient of PE in the model, the indirect effect is the product of the coefficients of PE and GA, and the total effect is the sum of direct and indirect effects.

**Supplementary Fig. S5**. The effect size and direction of significant CpGs identified before and after adjustment.

**Supplementary Fig. S6**. Differential methylation results without confounding adjustment for the study by Kashima et al. No significant CpGs were found before any confounding adjustment.

**Supplementary Fig. S7**. Differential methylation result after adding data from Fernando et al. (GSE66459). The study by Fernando et al. contains 11 idiopathic preterm samples and 11 full-term samples (see Methods). (A) The source of variance (SOV) analyses were conducted on cell types and clinical covariates. (B) Estimated cell-type proportion in the merged dataset. (C) Volcano plot of differential methylation result adjusted for clinical confounders and estimated cell proportion.

**Supplementary Table S1**. Multiple linear regression of each cell type on clinical variables.

**Supplementary Table S2**. Top 20 differentially methylated regions from the bumphunter algorithm.

## Abbreviations

ANOVA: analysis of variance; BH: Benjamini–Hochberg; BMI: body mass index; CB: cord whole blood; CBMC: cord blood mononuclear cell; CP: constrained projection; DAG: directed acyclic graph; DMP: differentially methylated probe; DMR: differentially methylated region; EOPE: early-onset preeclampsia; EWAS: epigenome-wide association studies; GA: gestational age; GEO: Gene Expression Omnibus; HiBR: Hawaii Biorepository; ILR: isometric log-ratio; logFC: log2-transformed fold change; LOPE: late-onset preeclampsia; NK: natural killer; nRBC: nucleated red blood cell; PDS: pathway deregulation score; PE: preeclampsia; PPROM: preterm premature rupture of membrane; PtLb: preterm labor; SOV: source of variance.
