# Supplementary material for: Cord blood DNA methylation and cell-type composition are not significantly associated with severe preeclampsia after cell-type and clinical covariate adjustment
Source: Gigascience. 2026 Jan 16;15:giag002. doi: 10.1093/gigascience/giag002 (PMC13014471; doi:10.1093/gigascience/giag002)
Supplement: giag002_Supplementary_material [file giag002_supplementary_material.docx]

**
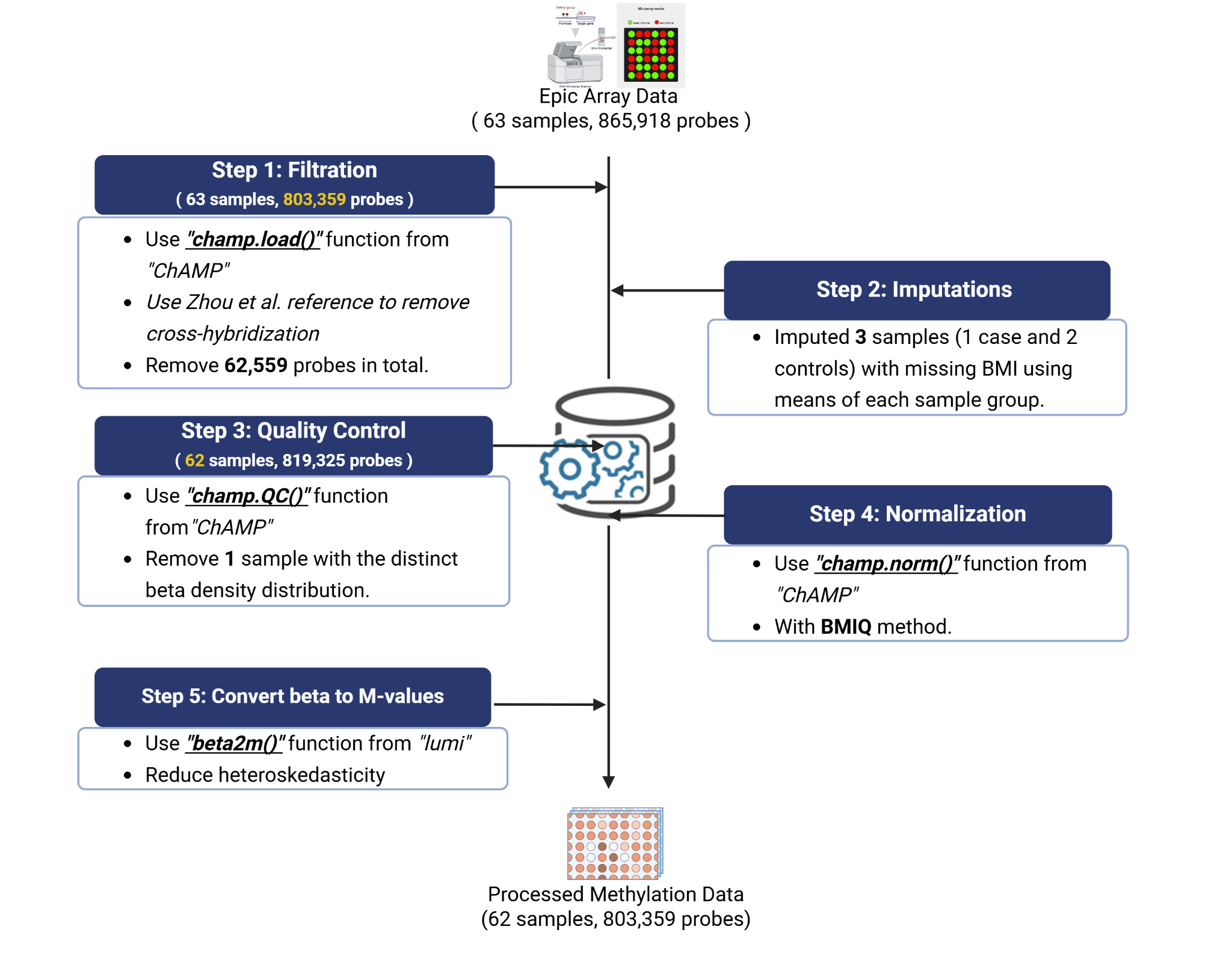
**

**Supplementary Figure 1:** Data Processing Workflow. The complete data pre-processing procedures consisted of filtration, imputation of missing values, quality control checks, normalization, and conversion of beta values to M-values. BMIQ: beta-mixture quantile normalization. Created in BioRender. Garmire, L. (2026) [https://BioRender.com/lyryqsk](https://biorender.com/lyryqsk)


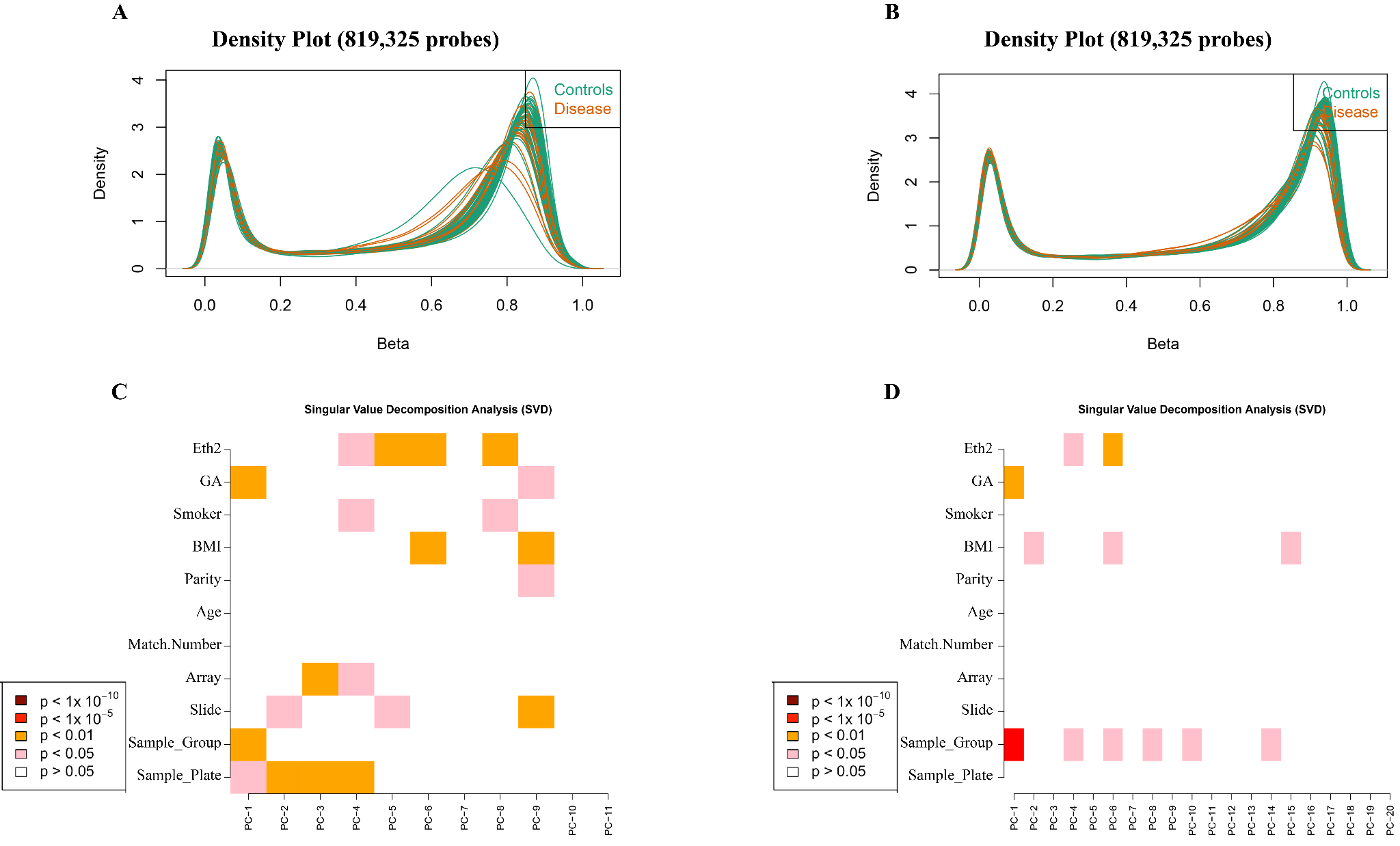


**Supplementary Figure 2: Data Quality Control.** (A-B) Density plots before and after BMIQ normalization and the removal of one control sample with a distinct beta density distribution. Color represents significance level.


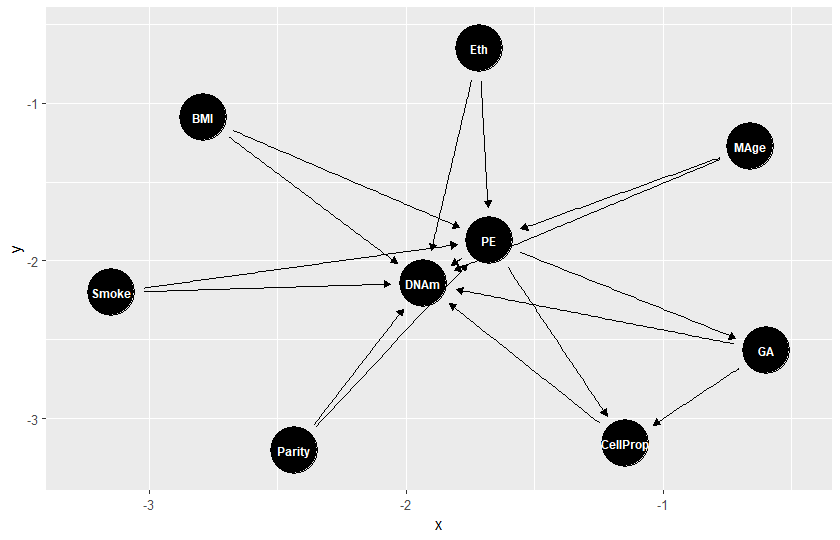


**Supplementary Figure 3: Directed acyclic graph (DAG) of assumed causal relationships.** Preeclampsia (PE) is the exposure, and DNA methylation change(DNAm) is the outcome. Pre-pregnancy BMI(BMI), maternal age (MAge), ethnicity (Eth), smoking during pregnancy (smoke) and parity are confounders that are associated with both PE and DNA methylation. Gestational age at delivery(GA) and estimated cell proportion (CellProp) are mediators between PE and DNA methylation, but most cord blood EWAS studies still adjust for them because researchers are more interested in the direct effect of PE on DNA methylation.


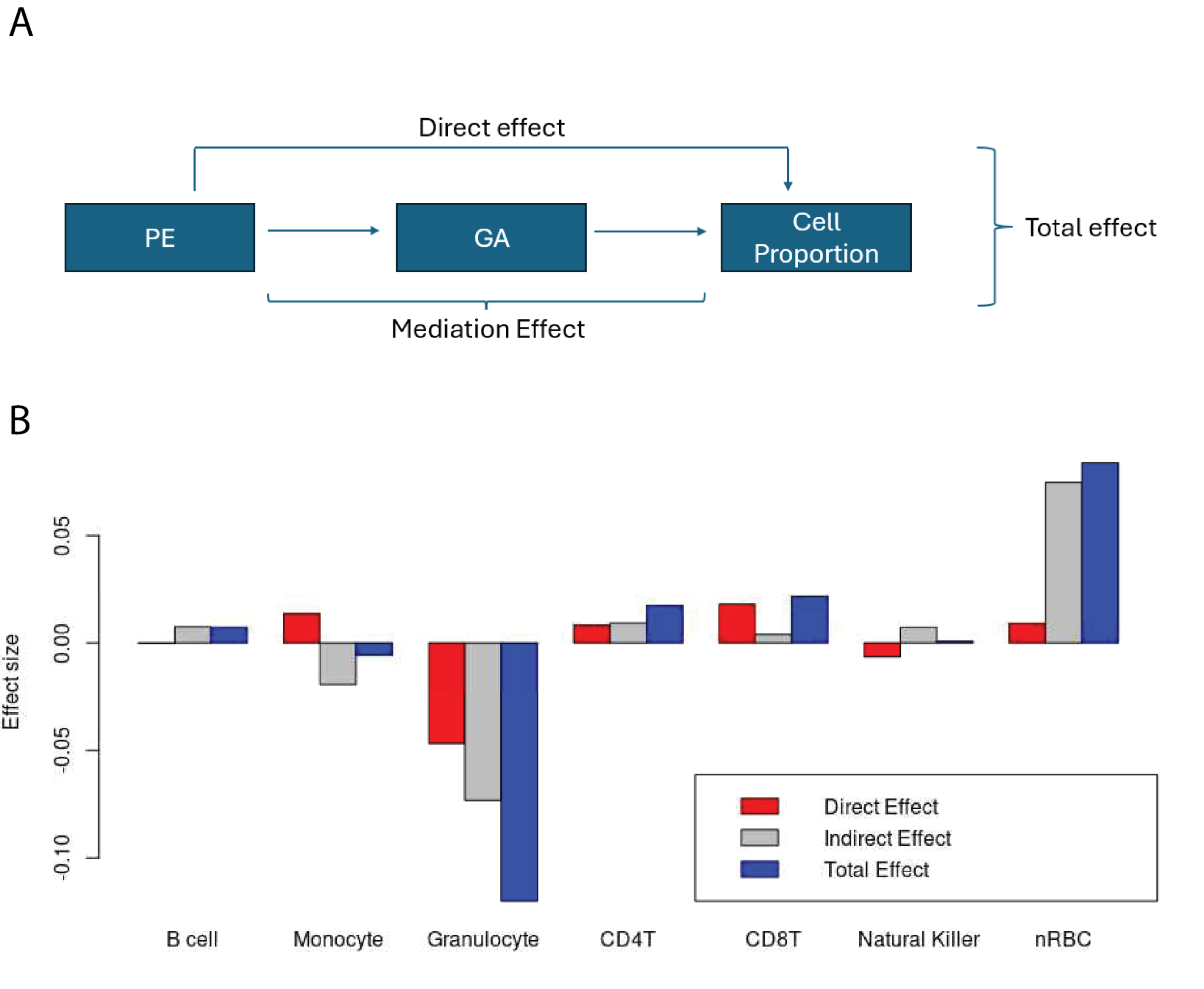


**Supplementary Figure 4: Mediation analysis of gestational age between PE and estimated cell proportion.** A) Illustration of the mediation effect of gestational age (GA) on the effect of preeclampsia (PE) on cell proportion on B)The effect size of direct effect, indirect effect and total effect of each cell proportion. The effects were calculated by regressing each cell proportion on preeclampsia and other clinical confounders-- the direct effect is the coefficient of PE in the model; the indirect effect is the product of the coefficients of PE and GA; the total effect is the sum of direct and indirect effects.


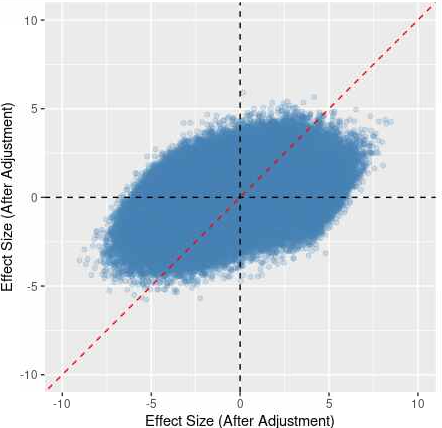


**Supplementary Figure 5: The effect size and direction of significant CpGs identified before and after the adjustment.**


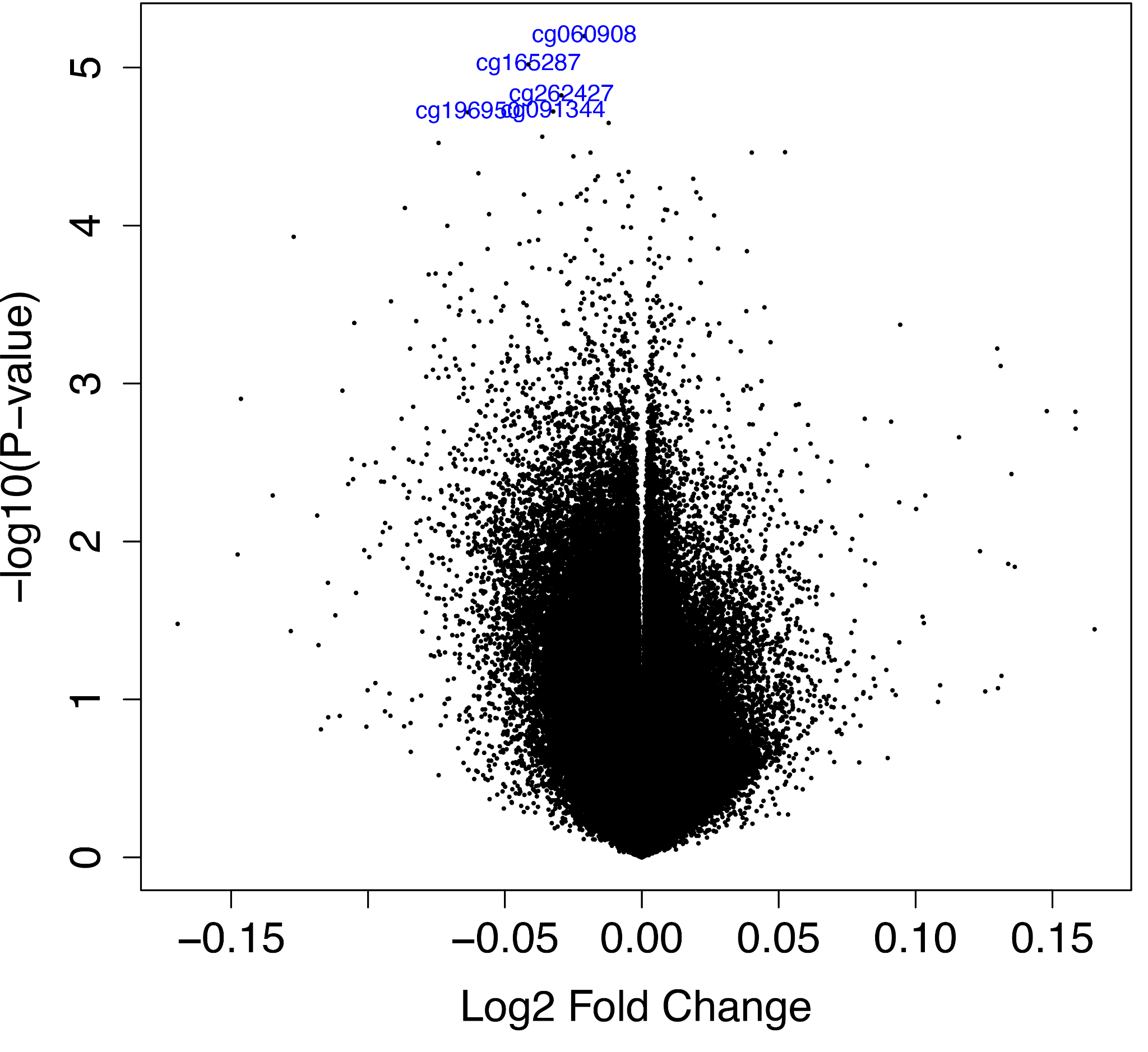


**Supplementary Figure 6: Differential methylation results without confounding adjustment for the study of Kashima K et al.** No significant CpGs were found before any confounding adjustment.


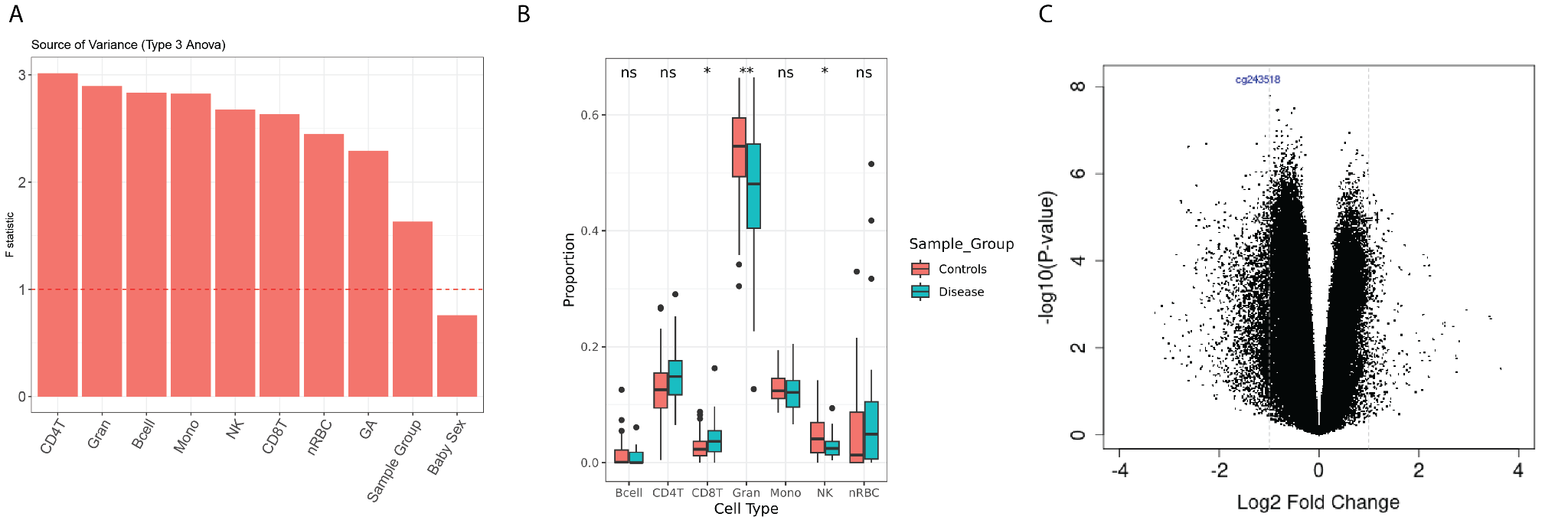


**Supplementary Figure 7: Differential methylation result after adding data from Fernando et al., (**[**GSE66459**](https://www.ncbi.nlm.nih.gov/geo/query/acc.cgi?acc=GSE66459)**).** Fernando et al. contain 11 idiopathic preterm samples and 11 full-term samples(see Methods). A) The Source of Variance (SOV) analyses were conducted on cell types and clinical covariates. B) Estimated cell type proportion in the merged dataset. C) volcano plot of differential methylation result adjusted for clinical confounders and estimated cell proportion.

**Supplementary Tables**

**Supplementary Table 1: Multiple linear regression of each cell type on clinical variables**

| **Supplementary Table 1: Linear regression of each cell type on clinical variables** | | | | | | | | | | | | | | |
| --- | --- | --- | --- | --- | --- | --- | --- | --- | --- | --- | --- | --- | --- | --- |
|  | *CD8T* | | *CD4T* | | *B cell* | | *Granulocyte* | | *Monocyte* | | *Natural Killer* | | *nRBC* | |
|  | *Coefficient* | *P-value* | *Coefficient* | *P-value* | *Coefficient* | *P-value* | *Coefficient* | *P-value* | *Coefficient* | *P-value* | *Coefficient* | *P-value* | *Coefficient* | *P-value* |
| ***(Intercept)*** | 9.55E-02 | 0.293 | 1.72E-01 | 0.312 | 1.49E-01 | **0.002** | -6.77E-01 | **0.018** | -2.22E-01 | **0.027** | 1.92E-01 | **0.013** | 1.38E+00 | **4.97E-07** |
| ***PE*** | 1.75E-02 | 0.102 | 9.08E-03 | 0.646 | -3.37E-03 | 0.532 | -1.63E-03 | 0.960 | 1.56E-02 | 0.178 | -9.51E-03 | 0.280 | -3.44E-02 | 0.227 |
| ***GA (week)*** | -1.05E-03 | 0.617 | -1.39E-03 | 0.724 | -3.27E-03 | **0.003** | 3.07E-02 | **1.43E-05** | 7.56E-03 | **0.001** | -3.27E-03 | 0.063 | -3.32E-02 | **2.04E-07** |
| ***BMI*** | -8.80E-04 | 0.087 | -6.23E-04 | 0.513 | -3.24E-04 | 0.214 | -1.41E-03 | 0.368 | 5.85E-04 | 0.290 | -5.94E-04 | 0.162 | 3.35E-03 | **0.016** |
| ***Age (year)*** | 8.94E-05 | 0.907 | 9.16E-04 | 0.524 | -3.25E-04 | 0.407 | 3.67E-03 | 0.125 | 6.72E-04 | 0.420 | -7.82E-04 | 0.222 | -4.58E-03 | **0.029** |
| ***Parity*** | 4.41E-04 | 0.868 | 6.30E-03 | 0.208 | 1.27E-03 | 0.349 | -1.31E-02 | 0.113 | 3.28E-03 | 0.257 | -4.15E-04 | 0.851 | 3.15E-03 | 0.657 |
| ***European Ancestry*** | 1.40E-03 | 0.895 | 2.33E-02 | 0.245 | 9.33E-03 | 0.090 | -7.70E-04 | 0.981 | -1.99E-02 | 0.089 | 5.95E-03 | 0.501 | -1.76E-02 | 0.538 |
| ***Pacific Islander*** | 1.57E-02 | 0.142 | 2.85E-02 | 0.154 | 1.10E-02 | **0.045** | 2.90E-02 | 0.376 | -1.25E-02 | 0.277 | -4.72E-03 | 0.591 | -7.88E-02 | **0.007** |
| ***Smoking*** | -3.07E-03 | 0.736 | -2.95E-02 | 0.087 | -2.16E-04 | 0.963 | 2.02E-02 | 0.473 | 1.46E-03 | 0.882 | 1.63E-02 | 0.034 | -2.46E-04 | 0.992 |

*The Asian race is used as the reference level.

**Supplementary Table 2: Top 20 Differentially Methylated Regions from the bumphunter algorithm**

chr: The chromosome where the DMR is located (e.g., chr1, chrX).

start: The genomic coordinate (base pair) where the DMR begins.

end: The genomic coordinate where the DMR ends.

value: The average estimated methylation difference (mean coefficient) between groups across CpGs in the region.

area: The sum of methylation differences across all CpGs in the DMR, reflecting both effect size and number of CpGs.

cluster: The identifier for the cluster of neighboring CpGs considered when detecting the DMR.

indexStart: The index (position) of the first CpG in the cluster contributing to this DMR.

indexEnd: The index (position) of the last CpG in the cluster contributing to this DMR.

L: The number of CpGs within the DMR.

clusterL: The total number of CpGs in the broader cluster that was scanned.

p.value: The unadjusted p-value tests whether the mean methylation difference (value) across CpGs in the region is significantly different from zero.

fwer: The family-wise error rate (FWER)–adjusted p-value controlling for multiple testing across all detected DMRs.

p.valueArea: The unadjusted p-value for the area statistic, testing the joint magnitude of methylation change and number of CpGs in the region.

fwerArea: The FWER-adjusted p-value for the area statistic.

| chr | start | end | value | area | cluster | indexStart | indexEnd | L | clusterL | p.value | fwer | p.valueArea | fwerArea |
| --- | --- | --- | --- | --- | --- | --- | --- | --- | --- | --- | --- | --- | --- |
| chr5 | 135415693 | 135416613 | 1.45 | 20.25379 | 372812 | 95297 | 95310 | 14 | 14 | 1.61E-06 | 0.032 | 4.24E-06 | 8.40E-02 |
| chr6 | 29648161 | 29648628 | -0.70 | 11.92384 | 388404 | 99779 | 99795 | 17 | 23 | 5.65E-06 | 0.112 | 2.42E-05 | 4.08E-01 |
| chr22 | 51016386 | 51017166 | -0.74 | 11.79156 | 304803 | 79434 | 79449 | 16 | 18 | 6.66E-06 | 0.132 | 2.48E-05 | 4.16E-01 |
| chr6 | 29911028 | 29911104 | -1.51 | 9.052504 | 388497 | 99999 | 100004 | 6 | 19 | 1.41E-06 | 0.028 | 5.65E-05 | 6.84E-01 |
| chr13 | 36871646 | 36872346 | 0.68 | 8.140038 | 124165 | 31581 | 31592 | 12 | 12 | 3.57E-05 | 0.54 | 7.51E-05 | 7.88E-01 |
| chr17 | 48585216 | 48585575 | 1.25 | 7.47451 | 199275 | 52911 | 52916 | 6 | 13 | 4.64E-06 | 0.088 | 9.42E-05 | 8.36E-01 |
| chr17 | 5402883 | 5403891 | -0.89 | 7.084428 | 188019 | 48717 | 48724 | 8 | 14 | 3.11E-05 | 0.468 | 0.000113 | 8.92E-01 |
| chr8 | 11666281 | 11666810 | -0.78 | 6.984819 | 438168 | 115543 | 115551 | 9 | 9 | 4.30E-05 | 0.56 | 0.000119 | 0.9 |
| chr4 | 81118188 | 81118794 | -0.86 | 6.861929 | 344634 | 88822 | 88829 | 8 | 8 | 3.65E-05 | 0.524 | 0.000127 | 9.04E-01 |
| chr22 | 43045530 | 43045990 | -0.93 | 6.521514 | 301876 | 78907 | 78913 | 7 | 9 | 3.03E-05 | 0.464 | 0.000148 | 0.924 |
| chr3 | 146261991 | 146262761 | 0.58 | 6.358762 | 325980 | 84796 | 84806 | 11 | 11 | 6.72E-05 | 0.772 | 0.000159 | 0.94 |
| chr1 | 248100345 | 248100614 | 1.19 | 5.933576 | 45837 | 11875 | 11879 | 5 | 8 | 9.08E-06 | 0.168 | 0.000197 | 9.56E-01 |
| chr5 | 128795058 | 128795827 | 0.96 | 5.761015 | 371362 | 94754 | 94759 | 6 | 19 | 3.07E-05 | 0.464 | 0.000214 | 0.964 |
| chr5 | 178986291 | 178986906 | -0.44 | 5.757518 | 381504 | 97756 | 97768 | 13 | 14 | 7.42E-05 | 0.792 | 0.000215 | 0.964 |
| chr1 | 153599487 | 153600156 | 0.40 | 5.576775 | 27935 | 7741 | 7754 | 14 | 15 | 7.38E-05 | 0.82 | 0.000236 | 0.972 |
| chr2 | 3642263 | 3642867 | 0.60 | 5.40927 | 237614 | 64697 | 64705 | 9 | 14 | 0.000112 | 0.848 | 0.000262 | 0.976 |
| chr8 | 17433625 | 17433926 | 0.74 | 5.167501 | 438754 | 115642 | 115648 | 7 | 19 | 9.75E-05 | 0.828 | 0.000299 | 0.988 |
| chr1 | 235292369 | 235292369 | -5.04 | 5.035918 | 43696 | 11536 | 11536 | 1 | 10 | 1.61E-06 | 0.028 | 0.000324 | 0.996 |
| chr8 | 143751533 | 143751796 | 1.27 | 5.070254 | 457083 | 118881 | 118884 | 4 | 8 | 8.07E-06 | 0.156 | 0.000317 | 0.996 |
| chr10 | 63660953 | 63661362 | 1.02 | 5.09352 | 55653 | 13495 | 13499 | 5 | 11 | 2.50E-05 | 0.42 | 0.000312 | 0.996 |
