# Supplementary material for: Cord blood DNA methylation and cell-type composition are not significantly associated with severe preeclampsia after cell-type and clinical covariate adjustment
Source: Gigascience. 2026 Jan 16;15:giag002. doi: 10.1093/gigascience/giag002 (PMC13014471; doi:10.1093/gigascience/giag002)
Supplement: giag002_GIGA-D-25-00219_Revision_1 [file giag002_giga-d-25-00219_revision_1.pdf]

GigaScience

Cell type proportions rather than DNA methylation in the cord blood show significant associations with severe preeclampsia

--Manuscript Draft--

**Manuscript Number:**	GIGA-D-25-00219R1
**Full Title:**	Cell type proportions rather than DNA methylation in the cord blood show significant associations with severe preeclampsia
**Article Type:**	Research
**Funding Information:**	U.S. National Library of Medicine (R01 LM012373) — Dr Lana Garmire<br>U.S. National Library of Medicine (R01 LM012907) — Dr Lana Garmire<br>Eunice Kennedy Shriver National Institute of Child Health and Human Development (R01 HD084633) — Dr Lana Garmire<br>National Institutes of Health (T32GM141746) — M.S. Xiaotong Yang

Abstract:

Abstract

Background

Preeclampsia is a severe pregnancy complication that threatens maternal and neonatal health and well-being. Previous studies on epigenome-wide association analysis (EWAS) of preeclampsia produced inconsistent results in cord blood tissues, and one possible explanation is their failure to rigorously adjust for cell proportions, gestational age, or other necessary variables.

Methods

Here, we calculated the DNA methylation change in cord blood from newborns affected by preeclampsia, using a multi-ethnic cohort from the Hawaii population (24 cases, 38 controls). We comprehensively adjusted for variables such as maternal age, body mass index (BMI), parity, and estimated the cell proportions. We also re-analyzed two previous datasets with adjustments to estimated cell proportions and conducted a pooled analysis by merging all three datasets together to increase the statistical power (58 cases, 71 controls). Lastly, we include idiopathic preterm (preterm delivery with no known reasons) cord blood samples (n=11) to disentangle the effect of severe preeclampsia and small gestational age.

Results

We showed that after adjusting cell type proportions and patient clinical characteristics, most of the so-called statistically significant CpG methylation changes associated with severe preeclampsia disappeared in our own data, two public datasets, and the pooled analysis combining all three datasets. This result still holds after including idiopathic preterm samples in the control group. Rather, we found that gestation progression is accompanied by statistically significant proportion changes in several cell types, such as granulocytes, nRBCs, CD8Ts, and B cells, which contribute to most DNA methylation differences between case and control groups. Preeclampsia has interactions on cell proportion changes in granulocytes, monocytes, and nRBCs.

Conclusions

In summary, our study shows that the previously reported differentially methylated patterns in cord blood are actually artifacts due to not properly adjusting for cell type heterogeneity, gestational age, and clinical covariates. Severe preeclampsia is not associated with statistically significant DNA methylation changes but changes in cell proportion. This finding alerts to the scientific rigor needed in EWAS.

**Corresponding Author:**	Lana Garmire<br><br>UNITED STATES
**Corresponding Author Secondary Information:**	
**Corresponding Author's Institution:**	
**Corresponding Author's Secondary**	

Powered by Editorial Manager® and ProduXion Manager® from Aries Systems Corporation

**Institution:**	
**First Author:**	Xiaotong Yang
**First Author Secondary Information:**	
**Order of Authors:**	Xiaotong Yang
	Wenting Liu
	Zhixin Mao
	Yuheng Du
	Cameron Lassiter
	Fadhl M. AlAkwaa
	Paula A Benny
	Lana Garmire
**Order of Authors Secondary Information:**	
**Response to Reviewers:**	Reviewer #1:<br><br>Credentials<br>I am an Associate Professor of Epidemiology and Health Equity at the Department of Public Health at the University of Copenhagen. I have been reviewing articles for over a decade for >30 biomedical journals, and I have been a key reviewer and statistical reviewer at the journal Diabetologia every year since 2020, with around 10 reviewed manuscripts each year for the past couple of years, with excellent editorial feedback. I obtained Clarivate Web of Science Academy certificates in Scientific Peer Review. My expertise is in cardiometabolic disease epidemiology, genetic epidemiology, biostatistics, machine learning, artificial intelligence, algorithmic fairness, and health inequalities. I have significant experience authoring and reviewing papers related to various omics studies, including genomics, epigenomics, and lipidomics. I am familiar with epigenetic association analysis pipelines and have reviewed, e.g., several papers related to epigenomics clocks. I also have the<br>necessary expertise to assess the study design and the statistical methodology of this paper. In fact, I have analyzed omics data using similar pipelines, and am familiar with some of the bioinformatics toolkits utilized in this paper (e.g., limma, pathway analysis tools). No AI technologies were used in the writing of this review other than a standard spell checker to correct typos.<br><br>Overall<br><br>I read the study by Yang X. et al. with interest. I was particularly interested in this study due to its dual aims: I really liked that the study had a strong biological hypothesis, but also aimed to highlight the importance of methodological considerations related to confounder adjustment. Based on my assessment, I see the second aim (statistical) fully delivered, but the first aim (biological/causal) potentially misleading or overstated. I fully agree with the authors in their final message that all studies should pay attention to proper confounder adjustment, including clinical variables and also cell proportions, when it comes to epigenomics analysis. However, the authors themselves fell into a trap of getting a bit lost in adjustments and not properly considering confounders. First, it appears that confounder selection was automated based on statistical relationships, which shows the lack of thought put into appropriately selecting these based on a priori hypotheses<br>(e.g., via drawing up a DAG). Second, the results are often misinterpreted. The authors claim a causal relationship (perhaps via poorly chosen wording) in the Discussion, and seem to conclude that cell proportions are key drivers in this study, whereas to me it appears, based on the results, that gestational age is the strongest correlate with DNA methylation patterns, rather than cell proportions. In my opinion, the following key findings emerged here, and this would be a logical order for presentation:1<br><br>1) Preeclampsia strongly associates with gestational age (known);<br><br>2) Cell proportions strongly correlate with gestational age (known);

Powered by Editorial Manager® and ProduXion Manager® from Aries Systems Corporation

3) CD8T cell proportions associate with preeclampsia even after gestational age adjustment (main effect, key novel finding);

4) Monocyte cell proportions show differential association with gestational age according to preeclampsia status (interaction, key novel finding).

Thank you for your suggestions. Our initial main motivation to submit this work, is to alert the readers the importance of adjusting for different cell types and clinical covariates (eg. GA) in the case study of preeclampsia. It was the surprising negative finding of lacking sufficient DNA methylation changes in association with severe preeclampsia after adjust these factors, which prompted us to share this story to the community. We figured that if we as bioinformaticians, had done the analysis inappropriately before, we should correct ourselves and let the community know how to do it properly. Thus, we think we should present the most significant negative results regarding the lack of association between DNA methylation changes and preeclampsia first. We then report other less striking but still important findings on cell proportions in association with gestational age next.

Thanks to the reminder of multiple hypothesis test, the CD8T cell proportion association with preeclampsia is not significant anymore, after gestational age adjustment.

Then, I would suggest the authors dive deeper into a "causal ideation": yes, DNA methylation associations disappear after adjustment for gestational age (and perhaps cell proportions), but this does not mean that they are not important. In fact, they can still be causal drivers of outcomes! But a more in-depth exploration into what comes first: the disease, the methylation, altered cell proportions, and what impacts what, would be a real added value to the Discussion of this paper. Please see my detailed comments below, hope they are helpful.

Introduction

- I suggest that you do not abbreviate preeclampsia as PE. It does not change the word count, and it is easier to read the full word out and avoid an unnecessary abbreviation.

Thank you for the suggestion. We have replaced all “PE” with “preeclampsia” in the manuscript.

- You mention that the Kazmi et al. article reported certain findings "after adjusting for cell types". Does this mean cell proportions, or how does this adjustment normally happen?

Yes, “adjusting for cell types” in Kazmi et al. means adjusting for estimated cell proportions. We included estimated cell proportions as variables in the linear regression model for differential DNA methylation analysis.

To avoid confusion, we changed “adjusting for cell type” to “adjusting for estimated cell proportion”.

- "(Kazmi et al) However, their results did not adjust for the gestational age, the major confounder of preeclampsia" - This sentence needs expansion. A confounder between what two factors? Can you explain how gestational age is a confounder in this setting (with respect to the key statistical criteria for being a confounder)?

Thank you for pointing this out. Our original wording was incorrect. Gestational age is a mediator not a confounder for DNA methylation outcome. We rephrased it to: “However, they did not adjust for gestational age, which is strongly associated with

both preeclampsia and DNA methylation, and therefore should be included as a key covariate.".

In this setting, gestational age (GA) at delivery is strongly associated with the exposure (preeclampsia) and also influences the outcome (cord-blood DNA methylation). The assumed causal pathway should look like:
PE → GA→ DNA methtylkation
Thus, GA is better described as a mediator rather than a confounder.

Still EWAS studies adjust for GA, in order to isolate methylation changes that are directly attributable to preeclampsia and independent of gestational age at the time of delivery [1,2]. In other words, we should understand in the epigenome changes due to preeclampsia itself, rather than these changes accompanying gestational progression.

We added clarifications and explanations for this claim.

[1] Herzog EM, Eggink AJ, van der Zee M, Lagendijk J, Willemsen SP, de Jonge R, Steegers EA, Steegers-Theunissen RP. The impact of early- and late-onset preeclampsia on umbilical cord blood cell populations. J Reprod Immunol. 2016 Aug;116:81-5. doi: 10.1016/j.jri.2016.05.002. Epub 2016 May 11. PMID: 27239988.
[2] Knihtilä HM, Kachroo P, Shadid I, Raissadati A, Peng C, McElrath TF, Litonjua AA, Demeo DL, Loscalzo J, Weiss ST, Mirzakhani H. Cord blood DNA methylation signatures associated with preeclampsia are enriched for cardiovascular pathways: insights from the VDAART trial. EBioMedicine. 2023 Dec;98:104890. doi: 10.1016/j.ebiom.2023.104890. Epub 2023 Nov 22. PMID: 37995466; PMCID: PMC10709000.

- The Introduction is excellent in clearly outlining a problem statement of poor confounder adjustment in epidemiological studies. I fully agree with the authors that this is often ignored in these studies. Key references are listed, showing a pattern of ignoring key confounders.
Thank you for your agreement

- Perhaps some more information in the Introduction on source tissues would be helpful to the reader. Are all the referred studies analyzed cord blood or rather maternal blood? What are the expected differences in results given tissue sources?

Thanks for your insight.

In the introduction section, we first mentioned studies [6-12], conducted on placentas [7-9, 11,12] and maternal blood[6,10]. After introducing the intergenerational effect of preeclampsia, we moved on overviewing studies on cord blood tissues[16, 18-20] and cord tissues [17].

We have specified the tissue origin of mentioned studies in the introduction. We also added a sentence to explain the expected difference between tissue types.

- "It is therefore essential to account for such heterogeneity, to improve the accuracy and sensitivity, and avoid biased conclusions" - Accuracy and sensitivity of what? From your problem statement and Introduction, my understanding is that this study relates to causal/biological discovery and highlighting methodological limitations (re: confounder adjustment). Accuracy and sensitivity are terms related to predictive statistics that have not been mentioned before, so these terms feel out of place. Besides, I do not think your study design is helpful for prediction.

Thank you for flagging this error. Indeed, "accuracy" and "sensitivity" are metrics used to evaluate prediction problems, and our study(EWAS) does not involve prediction tasks. Our intention was to emphasize robustness and validity. We will replace "accuracy" and sensitivity with "robustness and validity" to avoid confusion.

- "Particularly, to ensure that any differences in DNA methylation are due to confounding factors, the analysis needs to be adjusted for cell proportions" - I think you meant to say that "to ensure that differences in DNA methylation are NOT due to

confounding factors", right?

Thank you for pointing this out. We have fixed it in the manuscript.

- "In this study, we pay special attention to these issues to seek a plausible epigenomic association between severe PE and cord blood of offspring from PE patients." - This reads a bit clumsily, and wording should be changed to more accurately describe the goals of your study. Associations are not sought after "between PE and cord blood", but PE and various epigenetic and cellular markers IN cord blood.

We have rewritten this sentence following your suggestion: "In this study, we specifically address these issues to seek potential epigenomic and cellular markers in cord blood associated with severe preeclampsia."

- I am surprised that eclampsia is not mentioned in the Introduction as an even more severe condition. Is there any evidence related to the associations between epigenetic markers and cellular markers and eclampsia?

We thank the reviewer for this thoughtful comment. We now mention eclampsia as one of the most severe manifestations on the preeclampsia spectrum. However, the prevalence is very low (0.1–0.3% vs. ~3% for preeclampsia). Possibly due to this reason, in PubMed we did not find epigenome-wide association studies specifically on eclampsia.

Methods

- Please write out "OBGYNs" in full words at first mention.
We wrote out the full words for OBGYN (obstetricians and gynecologists) in the method section.

- "Severe PE was characterized by OBGYNs at Kapiolani Medical Center as sustained pregnancy induced hypertension(systolic/diastolic blood pressure >= 140/90) with urine protein and/or organ dysfunction." - First, what do you mean by organ dysfunction? Which organ? Second, my read is that both elevated BP and proteinuria are needed for this diagnosis, but organ dysfunction is optional. Or is it that either "elevated BP-proteinuria" or "elevated BP-organ dysfunction" combinations are satisfactory? Please clarify.

We apologize for the confusion. The definition we originally included corresponds to preeclampsia, whereas for severe preeclampsia the criteria we used were: among patients with preeclampsia (blood pressure ≥140/90 mmHg with proteinuria), those who additionally present with either (i) severe-range hypertension (blood pressure ≥160/110 mmHg), (ii) severe proteinuria (≥5 g in a 24-hour urine specimen or ≥3+ on two random urine samples collected at least 4 hours apart), or (iii) evidence of organ dysfunction(see below). Since the samples were collected before the new ACOG guidelines for preeclampsia diagnosis was released, the diagnosis criteria followed the older ACOG guidelines[1].
By organ dysfunction, we refer to the standard ACOG severe features, including thrombocytopenia (low platelet count), impaired liver function (elevated liver transaminases), progressive renal insufficiency (elevated creatinine or reduced urine output), pulmonary edema, or new-onset cerebral or visual disturbances.
In conclusion, “blood pressure ≥140/90 mmHg with proteinuria” and one or more of the three additional severe features are needed to diagnose severe PE. We have revised the Methods section to accurately reflect these diagnostic criteria.
[1] ACOG Committee on Practice Bulletins--Obstetrics. ACOG practice bulletin. Diagnosis and management of preeclampsia and eclampsia. Number 33, January 2002. Obstet Gynecol. 2002 Jan;99(1):159-67. doi: 10.1016/s0029-7844(01)01747-1. PMID: 16175681.

- "…and had cord blood samples remaining in the HiBR" - So your inclusion criterion is that there are samples remaining. Can this selection process introduce selection bias? I.e., for what reasons are cord blood samples removed from HiBR? If this is related to certain outcomes or baseline characteristics of the participants, then

	there is a selection mechanism that should be acknowledged, and the process's impact should be considered.<br><br>Thank you for raising this. Our inclusion criterion “samples remaining in HiBR” refers only to residual aliquot availability (i.e., not yet exhausted). Aliquot depletion in HiBR occurs for operational reasons: prior use by other projects, limited initial volume, or QC issues (e.g., hemolysis/low yield), rather than participant outcomes.<br><br>To ensure the samples are unbiased, we matched the case and controls by age, ethnicity, and pre-pregnancy BMI. As you can see in Table 1, patient characteristics between the case and control samples are not statistically different except for gestational age, which is on average shorter in the case group by clinical guidelines.<br><br>- "We evaluated sample integrity, purity, and concentration on the Nanodrop and removed samples of low quality." - What is the Nanodrop? How was low quality defined, and according to what metrics?<br>We thank the reviewer for this question. The NanoDrop (Thermo Fisher Scientific) is a microvolume UV–Vis spectrophotometer commonly used to assess nucleic acid samples. It measures absorbance at 260 nm to estimate DNA concentration, and the 260/280 nm and 260/230 nm absorbance ratios are used as indicators of purity. According to the manufacturer’s guidelines, a 260/280 ratio of ~1.8 is generally accepted as “pure” for DNA, while lower ratios may indicate protein contamination. Similarly, a 260/230 ratio close to 2.0 is considered optimal, with lower values suggesting contamination by salts, phenol, or other organics. These criteria are consistent with the NanoDrop user manual recommendations.<br>We have added a sentence to explain the quality threshold in the method section under the “The Hawaii Biorepository (HiBR) cohort” subsection.<br><br>- It would be very useful to present a directed acyclic graph (DAG) or a causal loop diagram (CLD) at some point in the manuscript. This could already happen in the Introduction stage, where you present the problem statement and your scientific hypothesis. This could formalize your hypothesis and also help you ascertain the confounder structure around the investigated statistical hypotheses. For some of the variables you chose to adjust for, I cannot immediately see whether they are potential confounders or mediators/colliders (in which case you should not adjust), so I suggest that you visualize everything with the DAG, and that you actually write out the three criteria for being a confounder in the Methods for the reader. I know that this is basic information, but this is key information for your manuscript that should be well understood by every reader of your paper to be able to fully appreciate the results (and who knows, you might uncover some new information through the visualization that you have not considered?).<br><br>Thank you for this helpful suggestion. We have added a directed acyclic graph (DAG) to the manuscript to formalize our study assumptions (supplementary figure 3) and clarify the relationships among variables. We did not use a causal loop diagram (CLD), as our associations are not cyclic. As shown in the figure, preeclampsia is treated as the exposure and DNA methylation is the outcome. Clinical variables (age, BMI, ethnicity, smoking, parity) are associated with both exposure and outcome and are not on causal pathway, so they are considered as upstream confounders. GA and cell proportion are on the causal pathways in the graph, they may mediate the observed DNA methylation changes and thus need to be adjusted, in order to tease out the actual direct association of preeclampsia to DNA methylation changes.<br>In EWAS study, researchers look for the cell-intrinsic alterations in dna methylation profile, which is independent of cell proportion and clinical mediators (eg. GA) of the sample tissue. It is standard practice in EWAS to adjust for estimated cell-type proportions (and related factors such as gestational age) to reveal the direct effect of disease on DNA methylation, independent of cell-mixture variation (Gervin et al., Clin Epigenet 2019 [22]; Zheng et al., Nat Methods 2018 [53]; Qi & Teschendorff, Clin Epigenet 2022 [55]; Merid et al., Genome Med 2020 [57]).<br>We also describe in the Methods section the three standard criteria for identifying confounders, as suggested.

Powered by Editorial Manager® and ProduXion Manager® from Aries Systems Corporation

- "The demographic and clinical information of the patients was collected and analyzed to identify any potential confounding effects." - What do you mean by this sentence? How was this done?

We appreciate the request for clarification. We rephrased this to “The demographic and clinical information of the patients was collected and analyzed to identify any potential association with DNA methylation”.

We collected demographic and clinical variables that are commonly reported to influence DNA methylation in cord blood (including maternal age, pre-pregnancy BMI, parity, smoking status, ethnicity, and gestational age at delivery). To evaluate their potential association with DNA methylation, we first included them in a source of variance (SOV) analysis, where each CpG site was regressed on these variables and F-statistics were averaged across sites. Variables with an average F-statistic greater than 1 (i.e., explaining more variance than noise) were retained as covariates and included in the downstream linear models for both CpG-level and region-level methylation analyses.

However, as you and other reviewers pointed out, a statistical test like SOV shouldn’t be used to identify confounders, because confounders are determined by relationships with exposure and outcome. We now only use SOV as a way to illustrate the contributors of DNA methylation changes. We clarified this change in both Methods and Results section.

- "To validate the observations in the HiBR cohort, we did an exhaustive search among published work of cord blood (including PBMC) DNA methylation in association with PE (16,17,23)" - I suggest that you include a brief supplementary document on how you undertook this "exhaustive search". What does this term mean? Is this a systematic review? Did you establish a search term or involve a librarian? You refer to three papers, but it is unclear how these articles were identified.

We thank the reviewer for this comment and agree that our wording was imprecise. By “exhaustive search,” we meant that we searched PubMed for all available studies on preeclampsia and cord blood DNA methylation at the time of our analysis, and included all results we found.

To avoid confusion, we have revised the text: “We searched PubMed for published studies of preeclampsia and cord blood DNA methylation and included all datasets identified (Ching et al., Herzog et al., and Kashima et al.).”

- "baby gender, and baby birth weight" - While, of course, this is understandable, I am wondering if "baby" is scientifically appropriate. I suggest replacing this with "infant".
Thank you for pointing this out. We have changed all “baby” in the manuscript to “infant. We kept the ‘babysex’ term in figure

- "including 10 early-onsets and 13 late-onsets" - You previously introduced abbreviations for these: EOPE, LOPE. I suggest you either stick to the abbreviations or remove the abbreviations altogether.
Thank you for flagging this! We have changed “early-onsets” and “late-onsets” to “EOPE” and “LOPE” to maintain consistency.

- "For Herzog EM et al and Kashima K et al datasets, we directly used the beta matrix deposited to GEO." - Please explain what the "beta matrix" is (I am assuming these are the summary level effect estimates), and what GEO stands for.

The “beta matrix” is the processed DNA-methylation data: each entry is a β-value between 0 and 1, representing the proportion of methylation at a given CpG site in a given sample. Each row corresponds to a CpG probe and each column corresponds to an individual sample. It’s not summarised but transformed directly from the raw

Powered by Editorial Manager® and ProduXion Manager® from Aries Systems Corporation

intensities.

GEO refers to the Gene Expression Omnibus, the NCBI-hosted public repository where high-throughput functional genomics datasets (including methylation arrays) are deposited and freely accessible.

We have added an explanation of “beta-value matrix” to the manuscript and the full name of GEO.

- "To disentangle the effect of PE and small GA" - Please refer to this as associations, as effect implies causality, which cannot be demonstrated by these results. In general, please be mindful of this throughout the manuscript and refer to all observed results as associations, and not effects. Even for betas, I would prefer "magnitude of association", rather than "effect size".
Thank you, this is an important suggestion. We have replaced the word “effect” with association to avoid misinterpretation for causal effect, except for fixed terms like “batch effect” or “confounding effect”.

- "idiopathic preterm birth samples" - What does idiopathic mean? Please explain for the general readership.
Certainly, "idiopathic preterm birth samples" refers to samples from cases of preterm birth where the cause is not known. According to Fernando et al., idiopathic preterm delivery initiated by either spontaneous preterm labor (PtLb) with intact membranes or preterm premature rupture of membranes (PPROM)[1]. We have added the explanation in the abstract and method section when we introduce the Fernando dataset.

[1]Fernando F, Keijser R, Henneman P, van der Kevie-Kersemaekers AM, Mannens MM, van der Post JA, Afink GB, Ris-Stalpers C. The idiopathic preterm delivery methylation profile in umbilical cord blood DNA. BMC Genomics. 2015 Sep 29;16:736. doi: 10.1186/s12864-015-1915-4. PMID: 26419829; PMCID: PMC4588235.

- For Fernando et al, you report: "We directly used the normalized matrix deposited to GEO." - Above, for Herzog and Kashima, you just referred to this as the "beta matrix". Is there a difference between the two (i.e., one is normalized, the other one not)?
Yes, the uploaded data in Fernando et al. is the normalized version of the aforementioned beta matrix. Usually, normalization is the first step to analyze a DNA methylation beta matrix.

We have changed the phrase to “normalized beta matrix” to avoid confusion.

- At first mention, please write out the full names of CD4T and CD8T cells.
Thank you, we have changed the abbreviation of cd4t, cd8t and b cell to their full name at the first mention.

- For all potential confounders listed under "Clinical confounders and source of variance analysis", please list categories and precise definitions. E.g., is BMI pre-pregnancy BMI (and when was it measured)? How is smoking status defined (is it during pregnancy, before pregnancy, what are the categories)? What are the categories for ethnicity?

We thank the reviewer for this helpful comment. In the revised manuscript, we now provide precise definitions and categories for all clinical confounders. Specifically, BMI refers to pre-pregnancy BMI measured at the first prenatal visit. Smoking status was defined as maternal self-reported smoking during pregnancy (yes/no). Ethnicity categories included Asian, Pacific Islander, Caucasian, and Other, as recorded in the medical record.

We have updated the Methods to clarify these definitions.

- "We performed the source of variance (SOV) analysis on these clinical variables and previously estimated sample cell proportions to identify important confounding variables that need to be adjusted, as done before." - Despite the inclusion of three

	references at the end of this sentence, this needs clarification. You explain a statistical process after this sentence, implying that confounders were selected purely based on statistical relationships. This is not appropriate. Confounders should be selected based on a priori assumptions about whether something is a confounder or not. The three criteria for being a confounder are: 1) The variable must be statistically associated with the exposure. 2) The variable must cause the outcome. 3) The variable must not be on a causal pathway. As suggested above, I would much prefer that you visualize your investigated association using a DAG and determine confounders based on that exploration.<br><br>Thank you this valuable comment. We agree that a priori biological and epidemiological reasoning is more appropriate than relying solely on statistical associations. In the revised manuscript, we now clarify that we first collected demographic and clinical variables commonly reported to influence cord blood DNA methylation (maternal age, pre-pregnancy BMI, parity, smoking status, ethnicity, and gestational age at delivery). These were treated as potential confounders based on prior knowledge. We then applied SOV analysis only as a confirmation step to assess their relative contribution to variation in methylation data, but we did not exclude any potential confounders based on the SOV results.<br><br>Additionally, we have included a DAG in the supplementary materials to make our assumptions about confounder relationships explicit.<br><br>- "The p-value of PE was adjusted with Benjamini-Hochberg (BH) adjustment (threshold of 0.05)." - This is appropriate and established in the literature. I suggest that you clarify that the threshold is an alpha type 1 error threshold.<br>Thank you, we have added the clarification that 0.05 is the type I error threshold.<br><br>- "We included study participants' GA (GA)," - There is a double abbreviation here, maybe this is a typo?<br>Yes, we apologize for the typo. We have changed the first GA to its full name, gestational age.<br><br>- Please do not use "significant(ly)" as a standalone term. Rather, please use "statistical significance" or "clinical significance", depending on the context. The term has been massively overused in biomedical literature, with a generally poor understanding among the readership.<br>Thank you for this important suggestion. Indeed, it’s easy to misunderstand the term “significance”. We have changed all “significant/significance” that refers to a difference that exceeds the threshold (type I error) in the text to “statistically significant/ statistical significance.”<br><br>- "We did not include data from Kashimi et al. because there was no significant cpg before the confounder adjustment." - I do not understand this justification, and this is potentially a flawed approach. My assessment is that all samples, regardless of the statistical significance of original associations (with or without adjustment), should be included in the meta-analysis. Otherwise, you will obtain biased estimates.<br>We apologize for the confusion in our original wording. We agree with the reviewer that excluding a dataset based on statistical significance would be a flawed approach and could bias results.<br>Our actual reason for not including the Kashima et al. dataset was that it was generated from cord blood PBMCs, whereas all other included datasets (our in-house cohort, Ching et al., and Herzog et al.) were based on whole cord blood. Because PBMCs lack granulocytes and therefore have a substantially different cell composition, including them would not be appropriate.<br>We revised this sentence accordingly.<br>- "To harmonize the datasets, we applied the combat function to remove batch effects while preserving sample group information" - Please correct this to "ComBat". There are further mentions of this in the manuscript, please double-check consistent naming.<br>Thank you, we have changed all “combat” to “ComBat” throughout the manuscript.<br><br>- "resulting in an inevitable correlation of PE cases and smaller gestational age." - To make this more precise, I would say that there is a positive correlation between PE

status and smaller gestational age.
Changed the original sentences to "resulting in an inevitable positive correlation of PE cases and smaller gestational age." as suggested.

- As a very minor comment, sometimes you write out gestational age, sometimes you write GA. Please be consistent (I suggest writing it out).
Thank you, we have changed all GA in the manuscript back to gestational age, to ensure consistency.
- What is the reason for applying FWER correction in one part of your analytic pipeline and B-H FDR in another part? Is there a specific reason for correcting for false positive rates vs. false discovery rates in the two analytic processes?

FWER appears only in our DMR analysis because the bumphunter algorithm outputs permutation-based, FWER-controlled p-values by design; this is not user-configurable. For DNA methylation analysis, we control multiplicity with Benjamini–Hochberg FDR, which is standard practice in epigenetic-wide association study(EWAS) and used by prior cord-blood methylation studies [1, 2]. Using FDR at the probe level ensures fair comparison of p-values across datasets and with the literature.

[1] Ching T, Ha J, Song MA, et al. Genome-scale hypomethylation in the cord blood DNAs associated with early onset preeclampsia. Clinical Epigenetics. 2015;7:21. DOI: 10.1186/s13148-015-0052-x. PMID: 25806090; PMCID: PMC4371797.
[2] Knihtilä HM, Kachroo P, Shadid I, Raissadati A, Peng C, McElrath TF, Litonjua AA, Demeo DL, Loscalzo J, Weiss ST, Mirzakhani H. Cord blood DNA methylation signatures associated with preeclampsia are enriched for cardiovascular pathways: insights from the VDAART trial. EBioMedicine. 2023 Dec;98:104890. doi: 10.1016/j.ebiom.2023.104890. Epub 2023 Nov 22. PMID: 37995466; PMCID: PMC10709000.

- It is very commendable that the authors made their code publicly available, amazing!
Thank you for the supportive comment.

Results

- "There is no significant difference ($P > 0.05$) in maternal age, parity, BMI, ethnicity, and smoking status." - Difference between what groups? Please be explicit.
We are referring to the difference between PE cases and controls, the detailed statistics(mean, standard deviation, p-values) of these variables are included in table 1.
We added the clarification in the text "Maternal characteristics were similar between the preeclampsia and control groups, except cases had significantly earlier gestational age at delivery ($P = 3.66\times10^{-6}$) (Table 1)"

- There is quite a bit of repetition in the Results related to key methodologies that were already explained in detail under Methods. These could be removed to shorten the Results section (e.g., around 2/3 of the first paragraph of the Results is repetition, referring to the same supplemental material files that you already referred to in the Methods).
Thank you for the suggestion. We've shortened the first paragraph of the Results section and kept only the key details, so that readers who skip the Methods can still follow the results.

- Where you present cell proportion differences between PE and control, you indicate "t" for some cell types, and "beta" for other cell types. What motivates this inconsistency? What do these represent? Is the scale here % difference between the two groups (if so, please state the unit explicitly).
Apologies for the typo—these should all be "t," referring to the t-statistics from two-sided t-tests. We used two-sided t-tests to compare the distribution of each cell type's proportion between cases and controls. All values are on the same scale: cell proportions expressed as a value between 0 to 1. They don't have a unit because they are a proportion.
We have revised the typo in the manuscript.

- "Since previous studies reported that maternal smoking significantly affects

DNA methylation (43)." - This is not a full sentence, please combine it with the next one.
Thanks for flagging this. We have made changes as suggested.

- "Since previous studies reported that maternal smoking significantly affects DNA methylation. To consider this we included the variable "smoking" despite its smaller effect on data variance." - This is precisely what I was referring to above at confounder adjustment selection. This is a more appropriate way of selecting potential confounders for adjustment.

Thank you. In the revision, we now make explicit that covariate selection is a priori and driven by domain knowledge rather than statistical tests (SOV). SOV is only used as a confirmation of selected variables.

Specifically, we select potential confounders based on prior literature and biological rationale: maternal smoking, maternal age, ethnicity/ancestry, BMI, and parity. Smoking is retained regardless of its small share of variance because it is a well-established determinant of DNA methylation.

Also, we adjust for estimated cell-type proportions to address cell-mixture bias in bulk cord-blood EWAS (standard practice), while using the SOV analysis only as a descriptive confirmation that these variables capture major sources of heterogeneity—not as a criterion for inclusion/exclusion.

- "In conclusion, we found that the observed DNA methylation variation among the whole cord blood samples is primarily associated with cell type differences rather than severe PE." - According to your analysis, this is an incorrect interpretation. You have no proof of that, as you adjusted the model for a range of other variables as well. E.g., it is entirely plausible (and probable) that gestational age alone could have resulted in the same results. In fact, this would be the way to assess this. To run parallel models and see whether the GA adjustment impacts the results, first. If differentially methylated sites remain, then you could assess whether cell proportions impact these associations.
Thank you for the comment. We removed this sentence, given the updated results.

- Related: The analysis you undertook using the Herzog et al. data, which does not have GA information, shows that adjustment for cell proportions alone makes all statistically significant sites disappear. So this is an interesting finding, but unfortunately still doesn't exclude the possibility of GA being the driver of these results.

Indeed, since gestational age (GA) information was not available in the Herzog et al. dataset, we could not directly evaluate or adjust for GA in that cohort. Therefore, while the disappearance of significant CpGs after cell proportion adjustment suggests that cell heterogeneity is a strong driver of the observed associations, we agree that this does not exclude the possibility that GA may also underlie these results.

- Instead of adjusted P values only, it would also be important to make a note of the magnitude of association differences as well.
Thank you, we have added the magnitude of changes to the association between cell proportions and GA.

- In the Results, you use the term "insignificant" as an opposite to statistically significant. This is incorrect and should not be used. Please use "not statistically significant".
Thank you, we have revised as suggested.

- After reading your Results and the parts related to the meta-analysis, I would like to emphasize again that I think excluding Kashima et al from the meta-analysis is not appropriate, and will bias your findings (just because they have null results before cell proportion adjustments, this does not mean that they should not be added to the meta-analysis, this is selective selection based on results, which is big NO). Furthermore, it is also a statistical possibility that by adjusting for cell type proportions, some differentially methylated sites BECOME statistically significant, further illustrating the flawed logic here.

	We appreciate your thoughtful comment and agree with your comments. As mentioned above, we still decided to exclude Kashima et al.'s dataset in the meta-analysis because it was generated from cord blood mononuclear cells (CBMCs), whereas our in-house cohort, Ching et al., and Herzog et al. were all based on whole cord blood. Since CBMCs lack granulocytes and have a substantially different overall cell composition from whole blood, they are not appropriate for the whole-blood based meta-analysis<br>As suggested, we performed a differential methylation analysis on the Kashima et al. dataset, adjusting for estimated cell proportions, gestational age, maternal age, BMI, parity, fetal sex, and batch effects. Consistent with our previous observation, this analysis still did not yield any statistically significant CpGs. We have revised the manuscript to clarify this rationale.<br>- "The proportions of monocytes also significantly increase as gestation progresses, after adjusting for other variables (p=0.019)." - What are the adjustments here, in these analyses?<br>We apologize for the error. This p-value was obtained by directly regressing the cell proportion of monocytes on gestational age. We didn't do any adjustments. We have changed the sentence to:<br>"The proportions of monocytes also significantly increase as gestation progresses (p=0.019)"<br><br>- "These trends of cell proportions are mostly the same in the case and control groups except for monocytes, which show a potential interaction effect between PE and gestational age." - THIS is perhaps your most important finding, sort of hidden in Results; you should emphasize this more!! Please include the magnitude of interaction and the P value. This result and methodology allow you to disentangle GA, cell proportions, and PE status.<br><br>Thank you for highlighting this important point. We agree that the potential interaction between preeclampsia (PE) and gestational age (GA) on monocyte proportions is a key finding that deserves more emphasis. In our regression model including GA, PE, and their interaction term, the interaction effect for monocytes was β = 0.006 with p-value = 0.009.<br>Additionally, in the new revision, PE and GA also has significant interaction in granocyte and nRBCs(Figure 4b). We have revised the Results to report these values explicitly and emphasized in the Discussion that this interaction may indicate differential regulation of monocyte proportions in PE across gestation.<br><br>- "None of the interaction terms between datasets and GA turn out to be significant, as the p-values in Fig. 5." - Please check grammar in this sentence.<br>Thank you, we have revised the sentence to be "None of the gestational-age interaction terms were statistically significant (Fig. 5A). A non-significant interaction p-value indicates that the GA–cell-proportion trends do not differ statistically between case and control group."<br><br>Discussion<br><br>- "In this study, we showed there is a lack of association between severe 401 PE and DNA methylation level changes, in the cord blood samples of the offspring of these PE patients from multiple cohorts." - This is the first sentence of the Discussion section, but this statement is not exactly true, as this finding is only true when you explicitly mention the adjustments, which is THE key learning here.<br><br>Thank you, we revised this sentence to: "In this study, we showed there is a lack of association between severe PE and offspring's cord blood DNA methylation changes after adjusting to cord blood cell types and clinicial covariates, from multiple cohorts."<br><br>- "Instead, severe PE manifests itself impact on the offspring of the affected mother by altering the proportions of some immune cell types in the blood." - This is a misleading and flawed conclusion. First, you cannot conclude a causal relationship. Second, this association is very, very likely to be almost completely explained by gestational age. There is only very limited evidence that supports a statistical association between cell types and PE. One is that CD8T remains borderline statistically significantly associated with severe PE vs. control status after adjustment

for GA, and the second is the observed statistically significant interaction for monocytes between PE and gestational age. I would not consider these two results very robust; however, they are the key novel results that make your article stand out. I suggest some rewording to appropriately show these interesting findings, but in a way that rather emphasizes these as emerging novel hypotheses
to study in further studies (and tone down overstated conclusions in THIS study).

We thank the reviewer for this important comment and agree that our original wording overstated the conclusions. We have revised the manuscript to remove any causal language and to more accurately reflect the evidence. Specifically, we now emphasize that, after adjustment for gestational age and other covariates, the only signals that remain are (i) a borderline statistical association of CD8T cell proportions with severe PE, and (ii) a statistically significant interaction between PE and gestational age for monocyte proportions.

We have changed the sentences to "Our results suggest that severe preeclampsia may be linked to subtle shifts in offspring immune cell proportions."

- "This observation is now expanded to their offspring, demonstrating the impact of PE." - This is a causal interpretation, which you should avoid.

Thank you—we agree and have removed causal phrasing. We have rephrased this line.
This observation is now expanded to their offspring, indicating an association between maternal PE and neonatal DNA methylation.
We also replaced similar terms (e.g., "impact," "effect") with "association/differences" throughout and added a note in the Discussion that causal inference is not supported by our design.

- I completely agree with your conclusion related to the statistical considerations about proper adjustment for cell proportion in these kinds of analysis. This is backed by your findings.
Thank you for the supportive comments.

- Related to Kazmi et al.: " However, they did not adjust for GA due to the concern of its confounding effect on PE." - This sounds like a very strange reasoning, as "concern of confounding" is exactly the reason for an adjustment.

Apologies for the confusion. We have revised this sentence accordingly "However, they did not adjust for gestational age, which may also mediate DNA methylation changes in addition to preeclampsia, thus need to be adjusted".

- "We showed that GA affects the methylation pattern both directly and through cell proportion change, …" - I do not think you can conclude these causal interpretations.
Following the comment, we revised this sentence to "Additionally, we show that gestational age is associated with cell proportion change, as reported before".

- "Instead, severe PE is associated with significant changes in several cell proportions in the cord blood." - This conclusion overstates the results.
Thank you for flagging this. We removed this sentence given the new updated results.

- I lacked a more structured highlight of the CD8T association (independent from GA), and the monocyte interaction effect. These two key pieces of evidence should be emphasized a bit more, as the most important indications of potential cell proportion-related findings in terms of PE (the rest of the results reflect cell proportion changes according to GA, in my reading).

Thank you for the insightful observations. We have revised the preprocessing steps based on reviewer comments, and in the new result, CD8T is no longer significantly associated with PE after adjusting for clinical variables (Figure 2C).

Additionally, PE shows significant interactions with gestational age in more cells

Powered by Editorial Manager® and ProduXion Manager® from Aries Systems Corporation

(granulocyte, monocyte and nRBC; Figure 4B). These intriguing findings may point to immune cell involvement in preeclampsia. However, as these results are preliminary and derived from computational deconvolution, we prefer to present them as hypotheses for future validation rather than central conclusions of this study.

To clarify this, we have added brief highlighting sentences in the Results and Discussion sections to note these associations as potential biological signals warranting further experimental investigation, while keeping the main focus of the manuscript on the lack of methylation associations after rigorous confounder adjustment.

- Further limitations of your study are that there is no information on key lifestyle variables, such as diet and physical activity, which both can have an impact on methylation patterns.

Thank you for the important suggestion. We have added this to our discussion section. “Lifestyle factors such as diet and physical activity may also influence DNA methylation patterns and should be included in future works.”

Items

For all items (and supplemental items), please always include abbreviations in the item legends. These are missing from a lot of items (cell types, EWAS, RE, etc.). For all figures, please edit the axis names, grouping names, and category names from the raw output from R to human-understandable names. E.g., the Figure 2D y-axis name is "Cell_Type_Proportion_Residuals", which sounds like an R variable name; you can make these nicer. For each figure, please replace "ns" with the exact P values (e.g., an adjusted P value of 0.051 would be ns, but informative).
Thank you for the suggestions! Here are the changes we made accordingly:
Added missed abbreviations in item legends, including EWAS, PBMC, cell type to captions.
Fixed the texts on figures that don’t read naturally. (Figure 2)
Replaced ns with exact p-values in Figure 2B, 2D, 3J, 4 and 5
We have changed the GA(PE) in Figure 5A to GA(Disease) to be consistent with previous figures.

Table 1: This table shows the sample characteristics. Due to the low sample size, I would suggest that the authors use nonparametric tests for the comparisons.
We have replaced t-tests with Wilcoxon test (nonparametric) to compare numeric variables.

Figure 1: This is a neat figure that shows the analytical pipeline.
Thank you.

Figure 2: The entire figure ended up very small in the downloaded PDF. Panel A) is very difficult to interpret, and not really related to the results in the paper; you never mention hierarchical clustering in the paper, so I would remove this plot. Panel B) is informative; however, it does not explain how different numbers of stars reflect different levels of statistical significance, in the figure legend. Why not include exact P values? Panel C) is informative, but would remove the coloring as colors here do not include extra information. Panel D) is informative - are these confounder-adjusted means?

Thank you for the helpful suggestions regarding Figure 2. We agree that Panel A is not central to our results and have removed it. For Panel B, we used exact P values instead of stars as suggested. For Panel C, we removed unnecessary coloring. Finally, for Panel D, we clarified in the figure legend that the boxplots display cell-type proportions after adjustment for confounders, and we explicitly indicate the modeling approach used. We also removed dashed lines from figure texts and increased the resolution.

Figure 3. Panels A-D-G) Please remove the color. In the other panels, in the volcano plots, please remove the written-out CpG names; these are irrelevant for this study. Panel J) Are these confounder-adjusted means?

We have removed the colors from SOV plots (Figure 3A, 3D, 3G). The cell proportion

	and test results shown in Figure J is not adjusted for confounders, because Herzog et al. doesn't provide any clinical/demographic information for their samples, so we are unable to adjust for confounders on the pooled dataset.<br><br>Figure 4. These plots are great, but I would be ...
Additional Information:	
Question	Response
Are you submitting this manuscript to a special series or article collection?	No
**Experimental design and statistics**<br><br>Full details of the experimental design and statistical methods used should be given in the Methods section, as detailed in our Minimum Standards Reporting Checklist. Information essential to interpreting the data presented should be made available in the figure legends.<br><br>Have you included all the information requested in your manuscript?	Yes
**Resources**<br><br>A description of all resources used, including antibodies, cell lines, animals and software tools, with enough information to allow them to be uniquely identified, should be included in the Methods section. Authors are strongly encouraged to cite Research Resource Identifiers (RRIDs) for antibodies, model organisms and tools, where possible.<br><br>Have you included the information requested as detailed in our Minimum Standards Reporting Checklist?	Yes
**Availability of data and materials**<br><br>All datasets and code on which the conclusions of the paper rely must be either included in your submission or deposited in publicly available repositories (where available and ethically appropriate), referencing such data using	Yes

a unique identifier in the references and in the “Availability of Data and Materials” section of your manuscript.<br><br>Have you have met the above requirement as detailed in our Minimum Standards Reporting Checklist?	
GigaScience has policies and guidelines in place for the use of generative AI-writing tools such as ChatGPT. If you have used such writing tools to assist with writing the manuscript this must be declared and cited in the text. Authors should not list AI-writing tools and other AI-assisted technologies as an author or co-author and should acknowledge that they are fully responsible for text generated or refined by AI-writing tools.<p><br><br>A summary of use (particularly in the introduction or among methods) needs to be included at the end of the paper, and the outputs should also be included as a supplementary file hosted in GigaDB or other open repositories. Please read our guidelines for more information. <p><br><br>By submitting to GigaScience, you are aware of the journal's AI-writing tools policy, and if you have declared use of such tools below, you have acknowledged this where appropriate in your manuscript and have made a summary of use and outputs available. <p><br><b>AI-assisted writing tools have been used in the preparation of this manuscript?	No

Manuscript
Click here to access/download;Manuscript;manuscript0730_revision (1).docx

Cell type proportions rather than DNA methylation in the cord blood show statistically significant associations with severe preeclampsia

Xiaotong Yang[1†], Wenting Liu[1†], Zhixin Mao[1], Yuheng Du[1], Cameron Lassiter[2], Fadhl M. AlAkwaa[1], Paula A Benny[2], Lana X Garmire[1*]

1. Department of Computational Medicine and Bioinformatics, University of Michigan, Ann Arbor, MI

2. University of Hawaii Cancer Center, Epidemiology, Honolulu, HI

3. Department of Neurology, University of Michigan, Ann Arbor, MI

† These authors contributed equally to the work

* corresponding author

Abstract

Background

Preeclampsia is a severe pregnancy complication that threatens maternal and neonatal health and well-being. Previous studies on epigenome-wide association analysis (EWAS) of preeclampsia produced inconsistent results in cord blood tissues, and one possible explanation is their failure to rigorously adjust for cell proportions, gestational age, or other necessary variables.

Methods

Here, we calculated the DNA methylation change in cord blood from newborns affected by preeclampsia, using a multi-ethnic cohort from the Hawaii population (24 cases, 38 controls). We

comprehensively adjusted for variables such as maternal age, body mass index (BMI), parity, and estimated the cell proportions. We also re-analyzed two previous datasets with adjustments to estimated cell proportions and conducted a pooled analysis by merging all three datasets together to increase the statistical power (58 cases, 71 controls). Lastly, we include idiopathic preterm (preterm delivery with no known reasons) cord blood samples (n=11) to disentangle the effect of severe preeclampsia and small gestational age.

Results

We showed that after adjusting cell type proportions and patient clinical characteristics, most of the so-called statistically significant CpG methylation changes associated with severe preeclampsia disappeared in our own data, two public datasets, and the pooled analysis combining all three datasets. This result still holds after including idiopathic preterm samples in the control group. Rather, we found that gestation progression is accompanied by statistically significant proportion changes in several cell types, such as granulocytes, nRBCs, CD8Ts, and B cells, which contribute to most DNA methylation differences between case and control groups. Preeclampsia has interactions on cell proportion changes in granulocytes, monocytes, and nRBCs.

Conclusions

In summary, our study shows that the previously reported differentially methylated patterns in cord blood are actually artifacts due to not properly adjusting for cell type heterogeneity, gestational age, and clinical covariates. Severe preeclampsia is not associated with statistically significant DNA methylation changes but changes in cell proportion. This finding alerts to the scientific rigor needed in EWAS.

Keywords

epigenome-wide association study, DNA methylation, preeclampsia, cell-type deconvolution, cord blood, women's health

Introduction

Preeclampsia is characterized by new-onset hypertension with proteinuria or one/more adverse conditions after 20 weeks of gestation[1]. Preeclampsia is one of the leading causes of maternal and prenatal morbidities and mortalities, affecting 2-8% of pregnancies globally and around 3.1% in the US[2,3]. Preeclampsia manifests as a diverse syndrome with multiple subtypes: based on blood pressure, clinical findings, and degree of proteinuria, preeclampsia can also be classified into severe preeclampsia or mild preeclampsia. Severe preeclampsia poses a greater risk to maternal and fetal health and may involve different pathways than mild preeclampsia of similar onset time[4]. Based on the onset time, preeclampsia can be divided into early-onset preeclampsia (EOPE), which occurs before 34 weeks of gestation, or late-onset preeclampsia (LOPE), which occurs after 34 weeks. If left untreated, preeclampsia may also progress into rare but life-threatening eclampsia, which causes maternal seizures. The complexity of preeclampsia poses additional challenges in understanding its root causes.

Numerous studies have been conducted to investigate the molecular mechanisms of preeclampsia[5], exploring genetic[6], epigenetic[7,8], transcriptomic[9], lipidomic[10], and telomere length[11,12] changes in placentas or blood of preeclampsia patients. Placenta reflects maternal–fetal interface changes and maternal blood reveals systemic maternal physiology adaptation during pregnancy, both unable to address impacts on offspring. On the other hand, the theory of the utero origin of diseases proposes that many chronic diseases are deeply rooted in the fetal stage, the very early phase of human development[13,14]. As the epigenome is both inheritable and prone to alteration by diseases, it is a plausible link mediating the effect of preeclampsia on offspring. Towards this, epigenome-wide

association studies or EWAS, has been attempted by different studies to investigate if preeclampsia affects offspring [15–19]. However, these studies did not reach coherent conclusions on the association between preeclampsia and cord blood DNA methylation profiles.

Using an EWAS approach, Ching et al. were the first to report significant global hypomethylation in cord blood from infants affected by EOPE, based on a study of 12 preeclampsia cases and 8 controls[15]. However, in the same year, Herzog et al. reported global hypermethylation in cord blood affected by EOPE compared to those in other preterm births, based on 10 EOPE samples[16], opposite to Ching et al. Later, Gao et al. claimed statistically significant hypermethylation affecting the expression of *AVPR1a, OXTR,* and *PKCB* in preeclamptic umbilical veins, with 40 preeclampsia cases[17]. Knihtila et al.[19] found differentially methylated CpGs in preeclamptic cord blood associated with the cardiovascular pathway using 16 preeclampsia cases. Different from the above individual studies, Kazmi et al.[18] conducted a large-scale pooled analysis (135 preeclampsia cases) and reported 26 new statistically significant CpG sites not previously associated with preeclampsia after adjusting for estimated cell proportion and certain clinical factors. However, their results did not adjust for the gestational age, which is strongly associated with preeclampsia[18]. One possible reason for the inconsistency in previous studies may be that many of these earlier studies did not adjust for cell-type heterogeneity[15,17,19] or gestational age[18]. A new study with good experimental design and rigorous statistical analysis is thus necessary to assess if these variables truly contributed to the discrepancy, and detect the real association between preeclampsia and methylation patterns without such interference.

Cord blood consists of many diverse cell types, each with a distinct epigenome profile[20,21]. Thus, the varying cell types in each sample can affect the overall DNA methylation profile at the bulk level[18]. It is therefore essential to account for such heterogeneity, to improve the robustness and validity, and to avoid biased conclusions of EWAS biomarker detection. Particularly, the analysis needs to be adjusted for cell proportions. Moreover, if essential clinical data such as gestational

ages are available (as they should be), then the analysis needs to be adjusted for the important clinical variables as well. In this study, we specifically address these issues to seek potential epigenomic and cellular markers in cord blood associated with severe preeclampsia.

Materials and Methods

The Hawaii Biorepository (HiBR) cohort

The umbilical cord whole blood DNA samples were obtained from HiBR. HiBR collected placenta, maternal, and cord blood samples from deliveries at Kapiolani Women and Children's Hospital from 2006 to 2013. It is one of the largest research tissue repositories in the Pacific region, containing specimens from more than 9250 mother-child pairs at the time of sample collection. The repository obtains informed consent from women post-partum to donate their placenta, umbilical cord, and excess cord and maternal blood (routinely collected for care purposes). Umbilical cord samples were collected immediately after delivery. Severe preeclampsia was characterized by obstetricians and gynecologists(OBGYN) at Kapiolani Medical Center: among patients with preeclampsia (blood pressure ≥140/90 mmHg with proteinuria), those who additionally present with either (i) severe-range hypertension (blood pressure ≥160/110 mmHg), (ii) severe proteinuria (≥5 g in a 24-hour urine specimen or ≥3+ on two random urine samples collected at least 4 hours apart), or (iii) evidence of organ dysfunction[22].

This is part of a parental study to investigate the multi-omics biomarkers for severe preeclampsia and how the mothers and their female offspring are protected against breast cancers later in life, using the previously biobanked samples in the Hawaii Birth Repository (HiBR)[10,12]. The parental study includes women with severe preeclampsia who delivered singletons and were matched 1-to-

1 by healthy preeclampsia-free deliveries based on maternal age, ethnicity, and pre-pregnancy BMI. For this nested cord blood study, we included those who delivered female babies and had cord blood samples remaining in the HiBR. We evaluated sample integrity, purity and concentration on Nanodrop and removed samples of low quality, with 260/280 (~1.8) and 260/230 (~2.0) ratios serving as standard quality thresholds. Variables such as maternal age, ethnicity, pre-pregnancy BMI, gestational age, parity, and self-reported smoking status during pregnancy were recorded. Ethnicity includes European ancestry, Asian, and Pacific Islanders. Patients with unknown ethnicity were excluded. Smoking is the self-reported smoking status(yes/no) during pregnancy. This cord blood cohort contains 24 women with severe preeclampsia and 38 preeclampsia-free healthy controls. The demographic and clinical information of the patients was collected and analyzed to identify any potential association with DNA methylation. Data usage was approved by IRB #CHS23976.

Additional Cohorts

To validate the observations in the HiBR cohort, we searched PubMed for published studies of preeclampsia and cord blood (including cord blood mononuclear cells) DNA methylation and included all public datasets identified[15,16,23]. We were able to obtain DNA methylome Illumina 450k data from the following studies: (1) Ching et al.[15] with 12 preeclampsia cases and 8 controls, along with 6 clinical variables available, including maternal age, maternal BMI, maternal ethnicity, gestational age, infant gender, and infant birth weight. (2) Herzog EM et al.[16] with 23 severe preeclampsia samples (including 10 EOPEs and 13 LOPEs) and 25 control samples, but no clinical variables available (GSE103253). (3) cord blood leukocytes from Kashima K et al.[23] with 20 preeclampsia cases and 90 controls, as well as 7 clinical variables available, including maternal age, maternal BMI, maternal smoking before pregnancy, parity, gestational age, infant gender, and delivery (GSE110828). We were unable to obtain data from the study by Kazmi et al. [19] as the data

are not publicly available. We obtained the raw data from Ching et al. The data was filtered, normalized, and then corrected for batch effects by slide and array, using the R package ”ChAMP”. For Herzog EM et al and Kashima K et al datasets, we directly used the processed DNA methylation data (beta matrix) deposited to GEO.

We also included cord blood samples from Fernando et al. (GSE66459)[24]. This dataset includes 11 idiopathic preterm birth samples and 11 full-term samples. Idiopathic preterm births are preterm deliveries with no known reasons, triggered by spontaneous preterm labor (PtLb) with intact membranes or preterm premature rupture of membranes (PPROM). All samples from this cohort are preeclampsia-free. We directly used the normalized beta matrix deposited to GEO.

Sample preparation

Umbilical cord blood samples were collected immediately after delivery. To prepare for DNA extraction, we first added three volumes of RBC Lysis Solution to one volume of clotted blood, which was then vortexed and incubated on a shaker for 15 minutes at room temperature. The sample was then centrifuged to pellet white blood cells and clot particulates, and the supernatant was carefully poured into a waste bucket. The pellet was resuspended in an additional volume of RBC Lysis Solution and incubated again for 15 minutes. After another centrifugation step, the supernatant was carefully removed, leaving behind 200 µL of residual liquid. The pellet was then vigorously resuspended in the residual liquid before being combined with a master mix of Cell Lysis and Proteinase K Solution. The mixture was vortexed and incubated at 55°C until homogeneous, with intermittent vortexing to facilitate digestion. Once homogenous, the samples were subjected to DNA purification on the Autopure Machine following the manufacturer's instructions.

DNA extraction and methylation profiling

DNA was extracted from prepared cord blood samples by HiBR using AllPrep DNA/RNA/Protein

Mini Kit (Qiagen, USA) according to the manufacturer's instructions. We obtained pre-extracted genomic DNA of whole cord blood samples from the HiBR and conducted DNA Illumina EPIC Beadchip assays through the University of Hawaii Cancer Center Genomics Core. Case and control samples are interleaved on the plate to ensure the sample group is independent of batch effects. We used the EZ DNA Methylation kit for the bisulfite conversion step (Zymo Research).

DNA methylation data pre-processing and quality control

We used the R package "ChAMP" for data pre-processing (**Supple Fig. 1).** We first filtered probes using the following criteria sequentially: (1) removing probes with a detection p-value above 0.01 on any sample (7,941 probes); (2) removing probes with a bead count <3 in at least 5% of samples (27,731 probes); (3) removing non-CG(ch) labelled probes (2,673 probes); (4) removing probes that align to multiple locations (25,194 probes). We used the newer annotation list by Zhou et al. to identify these probes[25]. During the quality control step, we removed 1 control sample with a distinct beta density distribution (**Supple Fig. 2A, 2B**). We normalized the remaining samples using BMIQ methods[26]embedded in "ChAMP". The preprocessed data matrix contains 62 samples and 819,325 probes. We converted the original methylation intensity (beta) to M-values using "beta2m" function from "lumi" package to reduce heteroskedasticity[27], where M-values are defined as the log2 ratio of the beta value of each probe.

Cell-type deconvolution in umbilical cord whole blood (CB)

Bulk-level DNA in umbilical cord whole blood (CB) includes at least 7 most common blood cell types: granulocytes, B lymphocytes (B cells), CD4+ lymphocytes (CD4T), cytotoxic T lymphocytes (CD8T), monocytes, natural killer cells (NK), and nucleated red blood cells (nRBC). Each sample may have different compositions of the cell types above, thus needing deconvolution. We adopted the Houseman's constrained projection (CP) algorithm[28] and a combined cord blood

cell type reference as recommended by Gervin et al [21]. This combined reference includes 263 cord blood cell type signatures from 4 previous large studies[29–32] and is used in many previous cord blood cell type estimation studies[33,34]. We applied this deconvolution approach consistently across all datasets, both in-house and public. We adjusted the estimated cell type proportions in all the differential analyses.

Clinical variables and source of variance analysis

We retrieved 6 commonly adjusted clinical variables in cord blood EWAS study from the biobank, including maternal age, ethnicity (including Asian, European ancestry, and Pacific Islander), parity, pre-pregnancy BMI, delivery gestational age, and smoking status[18]. We imputed 3 samples (including 1 severe preeclampsia and 2 controls) with missing BMI using the mean values of each sample group. To assess their relative contribution to methylation variation, we performed the source of variance (SOV) analysis on these clinical variables and previously estimated sample cell proportions as done before[10,35,36]. Specifically, we regressed each CpG site on all clinical variables and applied two-way ANOVA to each regression. Next, we averaged the F-statistics for each variable across CpG sites. Importantly, we only use SOV to confirm the importance of these variables, not to select; all variables were retained in the downstream regression models. A directed acyclic graph (DAG) illustrating the assumed causal relationships among exposure, outcome, and covariates is provided in the **supplementary figure 3**. Note that although gestational age and cell proportion are mediators on the causal pathway between PE and DNA methylation, in EWAS studies, it's still common practice to adjust for them, because researchers are interested in the direct effect of a disease on DNA methylation, which is more biologically meaningful[18,19].

CpG-level epigenome-wide association analysis (EWAS)

We calculated the differentially methylated probes (DMP) between severe preeclampsia cases and controls by fitting linear regression with empirical Bayes moderated statistics on each probe. The p-value of preeclampsia was adjusted with Benjamini-Hochberg (BH) adjustment (type I error threshold of 0.05). We included study participants' gestational age, BMI, parity status, ethnicity, and methylation-derived cell compositions in the linear model, and compared the result with that without adjusting for these variables using "limma" package[37]. We include batch variables (Slide and Array) in the regression as dummy variables to avoid the batch effect. Because estimated cell-type proportions are compositional (sum to 1), we transformed the vector of proportions using the isometric log−ratio (ILR) transform with a sequential binary partition to obtain an orthonormal set of k−1 coordinates from R package "compositions". The resulting ILR coordinates were included as covariates in the EWAS regression to adjust for cell composition without inducing collinearity. We defined hypermethylated CpGs as statistically significant CpGs with positive log2-transformed fold change (logFC) and hypomethylated CpGs as statistically significant CpGs with negative logFC, respectively. We calculated the Empirical null inflation factor (λ) for each EWAS result as estimated by the "BACON" package[38], which measures how much test statistics deviate from the expected null, indicating possible bias or confounding. An empirical null-based bias and inflation adjustment ("BACON" method) was applied when λ exceeded 1.2, a commonly accepted threshold for substantial inflation. We used volcano plots to illustrate the global DNA methylation changes between the cases and controls.

We also explored the potential cell-specific DNA methylation change in preeclampsia using cellDMC function from the Bioconductor package "EpiDISH".

Pooled analysis using three datasets

We also combined the three cohorts (in-house, Ching et al., Herzog et al.) and conducted a pooled analysis to improve the test power and produce more robust results. We did not include data from Kashimi et al. because Kashimi et al's data is extracted from cord blood mononuclear cells (CBMC), instead of the whole blood.

To reduce non-biological variation introduced by technical factors such as different array runs, slides, and laboratories, we harmonized the datasets following this pipeline: 1) we filtered the raw in-house EPIC data and raw 450k data from Ching et al. individually, then merged them based on overlapping CpG sites and normalized the dataset; 2) we combined it with the normalized beta matrix from Herzog et al., downloaded directly from GEO; 3) we applied the ComBat function to harmonize the datasets while preserving sample group information. We then calculated the differentially methylated probes between severe preeclampsia cases and controls using limma and plotted the volcano plot on the harmonized data. We estimated the cell proportion of the merged data using Houseman's CP method and the cell-type reference by Gervin et al. Lastly, we calculated differentially methylated probes again, with adjustment to the estimated cell types and plotted another volcano plot.

Including idiopathic preterm samples to decouple preeclampsia and small gestational age

Many preeclampsia patients are delivered preterm to avoid severe maternal complications, resulting in an inevitable positive correlation of preeclampsia status and smaller gestational age. To decouple preeclampsia and gestational age, we include another study, Fernando et al. (GSE66459)[24]. The dataset contains DNA methylation data of 11 idiopathic preterm and 11 term samples processed with the Illumina 450K Human methylation bead chip array. We merged our in-house data with Fernando et al.'s data by common CpGs, then computed the differentially methylated probes with adjustment of gestational age, infant sex and estimated cell proportion again using the limma

package. Similarly, we examined and corrected for potential bias and inflation using the empirical null distribution from the "bacon" R package[38].

Differential methylated regions (DMR)

To identify the differentially methylated regions (DMR), we used the bumphunter method from R package "bumphunter". We used the "clustermaker" function to identify clusters, with default parameters; the "bootstrap" method to generate null candidate regions; 0.2 and -0.2 as the upper and lower bounds of the candidate regions (more details of parameter choice see "5.3 - DMR.R" in the code). The result is adjusted for the same clinical variables and cell type proportions as the CpG-level differential analysis. We used the FWER method to adjust the P-values of each DMR and used adjusted P-value <0.05 as the cutoff for statistically significant DMRs.

Gene-level EWAS

We further examined the methylation signal difference between severe preeclampsia and controls at gene and pathway levels. We annotated the CpGs[39], selected those located on the promoter region and aggregated the methylation signals of all CpGs within a gene promoter by taking the geometric mean. We then compared the aggregated methylation signals between severe preeclampsia cases and controls, using linear regression with empirical Bayes moderated statistics. Additionally, we looked for pathways associated with promoter region methylation differences using the R package pathifier[40]. The *Pathifier* algorithm calculates a pathway deregulation score (PDS) for each sample and each pathway. We compared the pathway PDS scores in case and control samples adjusted for the same variables as the probe-level analysis; the result p-values were adjusted with Benjamini-Hochberg FDR (threshold p-values 0.05).

Software Usage and Code Availability

All analysis was done using R 4.1.2[41]. Spreeclampsiacifically, we used "ChAMP" (version 2.24.0) for data preparation[42], "limma" for differential methylation analysis[37], "EpiDISH" (version 2.10.0) for Houseman's CP cell-type deconvolution algorithm[43], bioconductor package "b9.bioc.FlowSorted.CordBloodCombined.450k" for cord blood DNA methylation reference and "IlluminaHumanMethylationEPICanno.ilm10b4.hg19" for data annotation[39]. All codes are available at https://github.com/lanagarmire/CB_DNAm_preeclampsia.

Results

Overview of Study Design and Cohort Characteristics

The overview of the study design is illustrated in **Fig. 1**. We analyzed whole cord blood from 24 severe preeclampsia cases and 39 controls collected at the Hawaii Biorepository (2006–2013). Maternal characteristics were similar between the preeclampsia and control groups, except cases had significantly earlier ($P = 3.66\times10^{-6}$) gestational age at delivery (Table 1). Genomic DNA was assayed on the Illumina EPIC BeadChip, and data were preprocessed with the R package *ChAMP* (**Supplementary Figure 2**), leaving 62 samples and 819,325 probes for analysis.

Associations between cord blood cell types and severe preeclampsia

To learn the association between severe preeclampsia and cord blood cell composition, we performed cell-type deconvolution using the cord blood cell type reference as recommended by Gervin et al[21] and Houseman's constrained projection (CP) deconvolution algorithm[28]. We assessed the importance cell type compositions and the clinical variables using the source of variance (SOV) analysis and ranked them by F-statistics. As shown in **Fig. 2A,** in addition to severe preeclampsia, gestational age, maternal BMI, and ethnicity also have statistically significant contributions to cell type heterogeneity. gestational age (F-statistic = 9.30) and maternal BMI (F-statistic = 3.10) rank higher than severe preeclampsia (F-statistic = 2.41), with gestational age being

the predominant influencing factor. We then compared the cell proportions between case and control groups (**Fig. 2B)**: Granulocyte proportions are statistically significantly lower (t = -0.11, P = 1.20e-06) in the severe preeclampsia group compared to the control group, while B cell (t = 6.93e-3, P = 0.01), nRBC cell (t= 0.08, P=4.7e-7) and CD8T cell (t=0.02, P=2.3e-3) proportions seem statistically significantly higher in cases. However, the apparent differences in cell proportions in cases vs controls could very well be due to other reasons (eg. gestational age) rather than the severe preeclampsia directly. With such caution, we calculated the association between cell proportion and severe preeclampsia again with linear regression. This time, we include gestational age, maternal age, ethnicity, BMI, and smoking status as covariate factors. For comparison, we plot the cell proportions in severe preeclampsia versus controls and report the p-values of preeclampsia from linear regression with other clinical covariates (**Fig. 2C**). The previously observed differences in cell proportions now disappear. The detailed linear regression results of cell proportion on clinical data can be found in **Supplementary Table 1**. We confirmed this finding and showed that the effect of PE on cell proportion is mediated by gestational age using a mediation analysis of gestational age on the effect of PE on each cell type (**Supplementary Figure 4**). In all, these results show that certain cord blood cell proportions vary among newborns, and it is important to adjust for cell type heterogeneity before interrogating the association with severe preeclampsia to study the direct effect of preeclampsia on DNA methylation change.

Lack of association between cord blood DNA methylation and severe preeclampsia

Considering that most previous cord blood EWAS studies overlooked the adjustment of cell types or other clinical covariates (such as gestational age) within their samples, we further investigated the impact of these factors on the differential methylation analysis. We conducted the source of variance (SOV) analysis of the DNA methylation matrix on cell type proportion and clinical covariates. Strikingly, all cell-type composition variables show the strongest and most dominant

explanatory power of variation in the methylation data (**Fig. 3A**), ranking even higher than the severe preeclampsia condition itself. After the cell proportions, severe preeclampsia case/control, gestational age, maternal age, parity, and ethnicity also have larger F-statistics than the error term (F-statistics=1), in descending order. Therefore, in the downstream analysis of differentially methylated CpGs, we adjusted for these variables.

As a comparison, we first conducted differential methylation analysis on severe preeclampsia without adjusting for any clinical covariate or cell-type proportions. The analysis reveals a global hypomethylation pattern (**Fig. 3B**). We identified 229,730 differentially methylated CpGs with adjusted p-values less than 0.05. Among these CpGs, 184,102 exhibited hypomethylation, while 45,628 displayed hypermethylation. However, when we redid the differential methylation analysis after adjusting for cell type heterogeneity and patient characteristics, all the CpGs differentially methylated above were no longer statistically significant (**Fig. 3C**). We also examined how the directions and magnitude of significant CpGs identified before adjustment changed after adjustment. Specifically, we used *t*-statistics from the **limma** regression as a measure of effect size. The magnitude of the effects substantially decreased after adjusting for gestational age and estimated cell-type proportions, and approximately 37% of CpGs reversed their direction of association (**Supplementary Figure 5)**. This large direction change and magnitude reduction supports the notion that the apparent methylation differences observed before adjustment were largely attributable to variation in cell composition rather than true disease-related methylation changes.

We also explored cell-specific differential methylation with “cellDMC” algorithm, and found no significant association.

Additionally, we conducted differentially methylated region (DMR) analysis, which also suggests no statistically significant association with severe preeclampsia when adjusted for the same variables of cell types and clinical contributors as done in **Fig. 3C** (**Supplementary Table 2**). We extended the differential methylation analysis to the gene level by aggregating the CpGs located on

gene promoters as the representation of promoter-level methylation (see **Methods**). Before adjusting for clinical covariates, gestational age, and cell proportions, we detected 4,767 differentially methylated genes. However, upon adjusting for both clinical covariates and cell types, none of the genes exhibited statistical significance. Similarly, we conducted a differential methylation analysis at the pathway level, employing the Pathifier algorithm (see **Methods)**. Before the adjustment, we detected 200 statistically significant pathways; after the adjustment, none of the pathways remained statistically significant.

To confirm this surprising finding that contradicts all previous EWAS studies on cord blood samples associated with preeclampsia, we re-analyzed all other available public whole cord blood (or CBMC) DNA methylation datasets associated with preeclampsia samples, from Ching et al.[15], Herzog EM et al.[16], and Kashima K et al.[23]. We estimated the cell type proportions the same way, using Houseman's CP algorithm and the new combined cord blood reference recommended by Gervin et al[21]. We conducted the SOV analysis by considering cell proportions in both studies and clinical variables whenever available (for Ching et al). SOV shows cell type proportions, gestational age, maternal age and preeclampsia are important variables, as they explain more variance than residual noise (F-statistics > 1) (**Fig. 3D, 3G**). For the dataset of Ching et al, we used the original analysis pipeline that did not consider any adjustment and reproduced the differential methylation results earlier, which reported 68,458 statistically significant CpGs (**Fig. 3E**). However, once we adjust for the gestational age, cell proportions and other clinical covariates, there are no longer statistically significant CpGs (**Fig. 3F).** For the newborn umbilical cord blood dataset of Herzog EM et al., we conducted a differential methylation analysis as well. Without adjustment, we obtained 24,597 statistically significant CpGs (**Fig. 3H**). Again, once we adjusted the cell type proportions (all seven major whole cord blood cell types: monocytes, CD4T, natural killer, granulocytes, nRBC, B cell, CD8T), there is no statistically significant CpG remaining (**Fig. 3I**).

For Kashima K et al. data, we found no statistically significant CpGs even before cell type adjustment (**Supplementary Figure 6**). Thus, using all three other available cord blood datasets, we confirm that there indeed is a lack of association between cord blood DNA methylation and severe preeclampsia.

To increase the statistical power, we further conducted a pooled analysis by combining our in-house data, data from Ching et al. and Herzog et al.. The three datasets were processed and harmonized as described in the Method section. Again, the differential methylation result of combined data shows many statistically significant CpGs are associated with preeclampsia before adjustment (**Fig. 3I**), but are not statistically significant after adjusting for cell proportions (**Fig. 3L**). Additionally, to best decouple preeclampsia and early gestational age, we included the idiopathic preterm samples from Fernando et al.[24]. The dataset has 11 preterm and 11 full-term samples whose cord blood DNA was processed with Illumina 450k beadchips. We merged them with our HiBR data and computed the differentially methylated CpGs (see **Methods**). Consistent with previous conclusions, we found no statistically significant difference between preeclampsia cases and non-preeclampsia controls, after adjusting for cell proportion, infant sex, and gestational age (**Supplementary Figure 7**). We thus conclude that there is a lack of association between cord blood DNA methylation change and severe preeclampsia.

Association between cord blood cell type and gestational age

Our earlier analysis shows that estimated cell proportions in cord blood are mostly correlated with gestational age (**Fig. 2C**). We thus conducted a more in-depth analysis. The most noticeable correlation comes from granulocytes, whose proportions increase from around 25% in week 32 to over 50% in week 40, with p<2.11e-11 (**Fig. 4A**). The proportions of monocytes also statistically significantly increase as gestation progresses (beta = 0.004, p=5.70e-3). On the contrary, B cell, CD8T, and nRBC statistically significantly decrease along the gestation (beta = -0.002, p=1.34e-3; beta = -0.003, p=2.79e-2; -0.026, p =9.28e-9). We also plotted the trends of cell type proportions

related to gestational age per sample group, by merging our HiBR and Fernando et al.'s data (**Fig. 4B**). These trends of cell proportions are mostly the same in the case and the control group, except for monocytes, granulocytes and nRBCs, which show a potential interaction between preeclampsia and gestational age.

Furthermore, we validated the trends of cell type proportion through gestational age using another public CBMC Illumina HumanMethylation450 BeadChip methylation dataset (GSE110828), which comprises 20 preeclampsia cases and 90 non-preeclampsia controls[23]. Both the case and control groups include large percentages of preterm samples, with the delivery gestational ages ranging from 26.14 to 38.14 weeks in cases and 23.00 to 41.29 weeks in controls. We deconvoluted the CBMC cell types using the same combined cord blood reference. To ensure comparability of cell proportions between CBMC and whole blood, we eliminated granulocytes, which are unique to whole blood and recalibrated the weights of the remaining cell types to sum to one. In both preeclampsia (**Fig. 5A**) and control samples (**Fig. 5B**), we observed the same increasing trend for monocytes, and the same decreasing trend for CD8T, nRBC cells, and B cells. For NK cells, while both cohorts show consistent trends of decrease in the control samples with gestational age, the trend in preeclampsia samples is not conclusive, possibly related to the small sample size (n=20) in the other CBMC cohort. Further, to test if the cell proportion trends in relationship with gestational age are consistent between the two datasets, we performed linear regression of each cell proportion (y-variables), over gestational age, CBMC vs. whole blood dataset stratification, and the interaction terms between datasets and gestational age, with adjustment of other variables for preeclampsia cases and controls separately. None of the gestational-age interaction terms were statistically significant (Fig. 5A). A non-significant interaction p-value indicates that the GA–cell-proportion trends do not differ statistically between the case and control groups. This shows that the association between gestational age and cell proportions is consistent and independent of datasets.

Discussion

In this study, we show that there is a lack of association between severe preeclampsia and offspring's cord blood DNA methylation changes after adjusting for cord blood cell proportions and clinical variables, from multiple cohorts. Interestingly, aligning with our observation, recent work by Campbell KA et al also showed that substantial placental cellular heterogeneity in preeclampsia contributes to previously observed bulk gene expression differences[44]. Despite the absence of CpG changes associated with severe preeclampsia, we observed noticeable variations in the CD8 T-cell proportions between severe preeclampsia cases and controls. We conclude that the primary impact of maternal preeclampsia on the offspring's cord blood is not methylation alteration. Our results suggest that severe preeclampsia may be linked to subtle shifts in offspring immune cell proportions. In particular, CD8T cells are statistically significantly higher in the infant's cord blood from the cases, after adjusting for other clinical covariates and gestational age, possibly due to the activation of the innate immune system of the babies from preeclampsia patients. Previous studies have confirmed the activation of T cells in preeclamptic patients[45,46]. This observation is now expanded to their offspring, indicating an association between maternal PE and neonatal DNA methylation.. The mechanism for increasing CD8T cells in cord blood is of interest for future work.

Furthermore, we noticed generally consistent associations between cell type compositions in cord blood and gestational age in two independent cohorts, regardless of the existence of severe preeclampsia conditions. Granulocyte proportion showed the strongest quantitative changes along gestational age, agreeing with the previous findings that granulocyte in the fetus increases drastically in the last trimester of pregnancy[47,48]. On the contrary, the estimated proportion of nRBC in our study decreases drastically as gestational age increases, also consistent with previous

findings[49–51]. Previously elevated nRBC was also found in preterm infants and infants with lower birth weight[52], providing additional supporting evidence to our finding. Braid et al. also reported decreases in B cells in cord blood using methylation-derived cell proportion. One explanation is that the large increase in granulocytes in late gestation makes the proportions of other cell types smaller, not necessarily the absolute value. Another possibility is that preterm infants are experiencing higher inflammation levels, which leads to a higher B cell proportion[47]. Taken together, these findings probe into the dynamic nature of cell type composition in cord blood during gestation, confirming the importance of cell type adjustment in methylation analysis.

Cell-type heterogeneity is one of the most influential factors affecting EWAS results. Over the years, various cell-type estimation methods for bulk-level epigenetic data have been developed [28,53], making the assessment of cell proportions effects possible. In 2013, Liu et al. first reported a large reduction in differentially methylated probes related to rheumatoid arthritis after adjusting for cell type composition in whole blood[54]. Some later studies confirmed the issue with cell type heterogeneity on EWAS research in other tissues, such as breast tissue, saliva, and placenta tissue[33,44,55–57], emphasizing the importance of adjusting for cell type heterogeneity in the EWAS of preeclampsia studied here. Kazmi et al. conducted a large pooled analysis on the association of preeclampsia and newborn DNA methylation and detected a small number of 26 statistically significantly associated CpGs. However, they did not adjust for gestational age, which is strongly associated with both preeclampsia and DNA methylation, and therefore should be included as a key covariate[18]. Additionally, we show that gestational age is associated with cell proportion change, as reported before[58]. More importantly, although several previous studies aimed to identify preeclampsia-related epigenetic biomarkers using cord blood samples[15–18], the importance of adjusting for cell type heterogeneity was mostly (3 out of 4) overlooked. Using as many as four different cohorts, our investigation here shows that ignoring the cell type heterogeneity and

gestational age may have contributed to biased EWAS associations with preeclampsia, as done previously by multiple studies.

Some caveats are worth mentioning for this study. First, all the cell types in the blood are computationally inferred, rather than experimentally measured. In theory, technology such as flow cytometry is likely more powerful for direct applications in EWAS or preeclampsia. Now, cord blood references only contain 6-7 cell types; a reference with finer grid cell types can yield more insights. However, for retrospective studies such as this one, where only whole blood DNA was available, or for historically archived bulk DNA methylation data, computational deconvolution is the only viable option. Also, infections such as chorioamnionitis, which unfortunately is not part of the collected clinical variables here, may also influence the association between gestational age and cell proportions by triggering a surge of neutrophils. However, given the small proportions of chorioamnionitis, this might not be a major issue. In addition to the variables we already considered, lifestyle factors such as diet and physical activity may also influence DNA methylation patterns and should be included in future works. Genetic variation (e.g., polymorphisms, relatedness, structural variants) may also contribute to DNA methylation differences[59], though our attempt to remove SNP-overlapping CpGs should mitigate these issues to some degree. Future follow-up histopathological investigation into the maternal decidua may examine vascular malperfusion, but it is beyond the scope of this study. Lastly, the cord blood samples studied here are modest in size (n = 62). The subsequent pooled analysis in Figure 3L has a total of 129 (preeclampsia = 58) cord blood samples, smaller than those in Kazmi et al.. We could not exploit the data in Kazmi et al. due to the lack of open access[18]. It will be highly interesting to combine these data together for re-analysis with statistical rigor in the future. We also aim to emphasize the importance of variable adjustment in EWAS studies of pregnancy-related diseases, as drastic changes in cell proportions during pregnancy can strongly influence DNA methylation patterns.

Conclusion

In summary, we could not find the evidence for statistically significant CpG methylation changes in EWAS analysis in association with severe preeclampsia, after adjusting for cell type heterogeneity and clinical variables such as gestational age. Additionally, many cell type proportions change drastically as pregnancy progresses.

List of abbreviations

EWAS: epigenome-wide association study

GA: gestational age

CBMC: cord blood mononuclear cell

NK: natural killer

nRBC: nucleated red blood cell

GEO: Gene Expression Omnibus

Data Availability Statement

All supporting DNA methylation data have been deposited in Gene Expression Omnibus(GEO). The associated accession number will be added upon approval.

Author Contributions

LXG conceived this project and supervised the study. XY and WL contributed equally to data analysis, result generation, and manuscript writing. ZM and YD assisted in the data processing. CL, FMA, and PAB contributed to sample collection, coordination and the experimental design of the DNA methylation. All authors have read, revised and approved the manuscript.

Funding

This research was supported by grants by NIH/NIGMS, R01 LM012373 and R01 LM012907 awarded by NLM, and R01 HD084633 awarded by NICHD to L.X. Garmire, and T32GM141746 by NIH to X.T Yang.

Acknowledgment

We thank the Genomics Shared Resources of the University of Hawaii Cancer Center for performing the methylation assays.

Compreeclampsiating Interests

LXG is a member of the Scientific Advisory Boards of Simulations Plus.

Materials & Correspondence

Correspondence to Lana X Garmire

Reference

1. Magee, L. A. *et al.* Guideline No. 426: Hypertensive Disorders of Pregnancy: Diagnosis, Prediction, Prevention, and Management. *Journal of Obstetrics and Gynaecology Canada* **44**, 547-571.e1 (2022).
2. Rana, S., Lemoine, E., Granger, J. P. & Karumanchi, S. A. Preeclampsia: Pathophysiology, Challenges, and Perspectives. *Circ Res* **124**, 1094–1112 (2019).
3. Gestational Hypertension and Preeclampsia: ACOG Practice Bulletin, Number 222. *Obstetrics & Gynecology* **135**, e237–e260 (2020).
4. Roberts, J. M. *et al.* Subtypes of Preeclampsia: Recognition and Determining Clinical Usefulness. *Hypertension* **77**, 1430–1441 (2021).
5. Benny, P. A., Alakwaa, F. M., Schlueter, R. J., Lassiter, C. B. & Garmire, L. X. A review of

omics approaches to study preeclampsia. *Placenta* **92**, 17–27 (2020).

6. Zhao, L., Bracken, M. B. & DeWan, A. T. Genome-Wide Association Study of Pre-Eclampsia Detects Novel Maternal Single Nucleotide Polymorphisms and Copy-Number Variants in Subsets of the Hyperglycemia and Adverse Pregnancy Outcome (HAPO) Study Cohort. *Annals of Human Genetics* **77**, 277–287 (2013).
7. Anton, L., Brown, A. G., Bartolomei, M. S. & Elovitz, M. A. Differential Methylation of Genes Associated with Cell Adhesion in Preeclamptic Placentas. *PLoS ONE* **9**, e100148 (2014).
8. Yeung, K. R. *et al.* DNA methylation profiles in preeclampsia and healthy control placentas. *American Journal of Physiology-Heart and Circulatory Physiology* **310**, H1295–H1303 (2016).
9. Gormley, M. *et al.* Preeclampsia: novel insights from global RNA profiling of trophoblast subpopulations. *American Journal of Obstetrics and Gynecology* **217**, 200.e1-200.e17 (2017).
10. He, B. *et al.* The maternal blood lipidome is indicative of the pathogenesis of severe preeclampsia. *Journal of Lipid Research* **62**, 100118 (2021).
11. Biron-Shental, T. *et al.* Short telomeres may play a role in placental dysfunction in preeclampsia and intrauterine growth restriction. *American Journal of Obstetrics and Gynecology* **202**, 381.e1-381.e7 (2010).
12. Yang, X. *et al.* Placental telomere length shortening is not associated with severe preeclampsia but the gestational age. *Aging* **15**, 353–370 (2022).
13. Barker, D. J. P. The origins of the developmental origins theory. *Journal of Internal Medicine* **261**, 412–417 (2007).
14. Barker, D. J. P. In utero programming of cardiovascular disease. *Theriogenology* **53**, 555–574 (2000).
15. Ching, T. *et al.* Genome-scale hypomethylation in the cord blood DNAs associated with

early onset preeclampsia. *Clin Epigenet* **7**, 21 (2015).

16. Herzog, E. M. *et al.* The impact of early- and late-onset preeclampsia on umbilical cord blood cell populations. *Journal of Reproductive Immunology* **116**, 81–85 (2016).
17. Gao, Q. *et al.* Promoter methylation changes and vascular dysfunction in pre-eclamptic umbilical vein. *Clin Epigenet* **11**, 84 (2019).
18. Kazmi, N. *et al.* Hypertensive Disorders of Pregnancy and DNA Methylation in Newborns: Findings From the Pregnancy and Childhood Epigenetics Consortium. *Hypertension* **74**, 375–383 (2019).
19. Knihtilä, H. M. *et al.* Cord blood DNA methylation signatures associated with preeclampsia are enriched for cardiovascular pathways: insights from the VDAART trial. *eBioMedicine* **98**, 104890 (2023).
20. Yuan, V. *et al.* Cell-specific characterization of the placental methylome. *BMC Genomics* **22**, 6 (2021).
21. Gervin, K. *et al.* Systematic evaluation and validation of reference and library selection methods for deconvolution of cord blood DNA methylation data. *Clin Epigenet* **11**, 125 (2019).
22. ACOG Committee on Practice Bulletins--Obstetrics. ACOG practice bulletin. Diagnosis and management of preeclampsia and eclampsia. Number 33, January 2002. *Obstet Gynecol* **99**, 159–167 (2002).
23. Kashima, K. *et al.* Identification of epigenetic memory candidates associated with gestational age at birth through analysis of methylome and transcriptional data. *Sci Rep* **11**, 3381 (2021).
24. Fernando, F. *et al.* The idiopathic preterm delivery methylation profile in umbilical cord blood DNA. *BMC Genomics* **16**, 736 (2015).
25. Zhou, W., Laird, P. W. & Shen, H. Comprehensive characterization, annotation and innovative use of Infinium DNA methylation BeadChip probes. *Nucleic Acids Res* **45**, e22 (2017).

26. Teschendorff, A. E. *et al.* A beta-mixture quantile normalization method for correcting probe design bias in Illumina Infinium 450 k DNA methylation data. *Bioinformatics* **29**, 189–196 (2013).

27. Du, P. *et al.* Comparison of Beta-value and M-value methods for quantifying methylation levels by microarray analysis. *BMC Bioinformatics* **11**, 587 (2010).

28. Houseman, E. A. *et al.* DNA methylation arrays as surrogate measures of cell mixture distribution. *BMC Bioinformatics* **13**, 86 (2012).

29. Bakulski, K. M. *et al.* DNA methylation of cord blood cell types: Applications for mixed cell birth studies. *Epigenetics* **11**, 354–362 (2016).

30. Lin, X. *et al.* Cell type-specific DNA methylation in neonatal cord tissue and cord blood: a 850K-reference panel and comparison of cell types. *Epigenetics* **13**, 941–958 (2018).

31. Gervin, K. *et al.* Cell type specific DNA methylation in cord blood: A 450K-reference data set and cell count-based validation of estimated cell type composition. *Epigenetics* **11**, 690–698 (2016).

32. De Goede, O. M., Lavoie, P. M. & Robinson, W. P. Cord blood hematopoietic cells from preterm infants display altered DNA methylation patterns. *Clin Epigenet* **9**, 39 (2017).

33. Alfano, R. *et al.* Cord blood epigenome-wide meta-analysis in six European-based child cohorts identifies signatures linked to rapid weight growth. *BMC Med* **21**, 17 (2023).

34. Kotsakis Ruehlmann, A. *et al.* Epigenome-wide meta-analysis of prenatal maternal stressful life events and newborn DNA methylation. *Mol Psychiatry* **28**, 5090–5100 (2023).

35. Chen, Y. *et al.* Maternal plasma lipids are involved in the pathogenesis of preterm birth. *GigaScience* **11**, giac004 (2022).

36. Dickson, D. W. *et al.* Extensive transcriptomic study emphasizes importance of vesicular transport in C9orf72 expansion carriers. *acta neuropathol commun* **7**, 150 (2019).

37. Ritchie, M. E. *et al.* limma powers differential expression analyses for RNA-sequencing and microarray studies. *Nucleic Acids Research* **43**, e47–e47 (2015).

38. the BIOS Consortium, Van Iterson, M., Van Zwet, E. W. & Heijmans, B. T. Controlling bias and inflation in epigenome- and transcriptome-wide association studies using the empirical null distribution. *Genome Biol* **18**, 19 (2017).

39. Kasper Daniel Hansen [Cre, A. IlluminaHumanMethylationEPICanno.ilm10b4.hg19. Bioconductor https://doi.org/10.18129/B9.BIOC.ILLUMINAHUMANMETHYLATIONEPICANNO.ILM10B4.HG19 (2017).

40. Drier, Y., Sheffer, M. & Domany, E. Pathway-based personalized analysis of cancer. *Proc. Natl. Acad. Sci. U.S.A.* **110**, 6388–6393 (2013).

41. R Core Team. R: A language and environment for statistical computing. R Foundation for Statistical Computing (2021).

42. Tian, Y. *et al.* ChAMP: updated methylation analysis pipeline for Illumina BeadChips. *Bioinformatics* **33**, 3982–3984 (2017).

43. Andrew E. Teschendorff <A. Teschendorff@Ucl.Ac.Uk>, S. C. Z. C. EpiDISH. Bioconductor https://doi.org/10.18129/B9.BIOC.EPIDISH (2017).

44. Campbell, K. A. *et al.* Placental cell type deconvolution reveals that cell proportions drive preeclampsia gene expression differences. *Commun Biol* **6**, 264 (2023).

45. Faas, M. M., Spaans, F. & De Vos, P. Monocytes and Macrophages in Pregnancy and Pre-Eclampsia. *Front. Immunol.* **5**, (2014).

46. Lager, S. *et al.* Abnormal placental CD8 $^{+}$ T-cell infiltration is a feature of fetal growth restriction and pre-eclampsia. *The Journal of Physiology* **598**, 5555–5571 (2020).

47. Braid, S. M., Okrah, K., Shetty, A. & Corrada Bravo, H. DNA Methylation Patterns in Cord Blood of Neonates Across Gestational Age: Association With Cell-Type Proportions. *Nursing Research* **66**, 115–122 (2017).

48. Rolim, A. C. B. *et al.* BLOOD CELLS PROFILE IN UMBILICAL CORD OF LATE PRETERM AND TERM NEWBORNS. *Rev. paul. pediatr.* **37**, 264–274 (2019).

49. Hebbar, S., Misha, M. & Rai, L. Significance of Maternal and Cord Blood Nucleated Red Blood Cell Count in Pregnancies Complicated by Preeclampsia. *Journal of Pregnancy* **2014**, 1–7 (2014).
50. Perrone, S. Nucleated red blood cell count in term and preterm newborns: reference values at birth. *Archives of Disease in Childhood - Fetal and Neonatal Edition* **90**, F174–F175 (2005).
51. Hermansen, M. C. Nucleated red blood cells in the fetus and newborn. *Arch Dis Child Fetal Neonatal Ed* **84**, F211–F215 (2001).
52. Wang, X. *et al.* Epigenome-wide association study of bronchopulmonary dysplasia in preterm infants: results from the discovery-BPD program. *Clin Epigenet* **14**, 57 (2022).
53. Zheng, S. C., Breeze, C. E., Beck, S. & Teschendorff, A. E. Identification of differentially methylated cell types in epigenome-wide association studies. *Nat Methods* **15**, 1059–1066 (2018).
54. Liu, Y. *et al.* Epigenome-wide association data implicate DNA methylation as an intermediary of genetic risk in Rheumatoid Arthritis. *Nat Biotechnol* **31**, 142–147 (2013).
55. Qi, L. & Teschendorff, A. E. Cell-type heterogeneity: Why we should adjust for it in epigenome and biomarker studies. *Clin Epigenet* **14**, 31 (2022).
56. Middleton, L. Y. M. *et al.* Saliva cell type DNA methylation reference panel for epidemiological studies in children. *Epigenetics* **17**, 161–177 (2022).
57. Merid, S. K. *et al.* Epigenome-wide meta-analysis of blood DNA methylation in newborns and children identifies numerous loci related to gestational age. *Genome Med* **12**, 25 (2020).
58. Haftorn, K. L. *et al.* Nucleated red blood cells explain most of the association between DNA methylation and gestational age. *Commun Biol* **6**, 224 (2023).
59. Zhou, W., Triche, T. J., Laird, P. W. & Shen, H. SeSAMe: reducing artifactual detection of DNA methylation by Infinium BeadChips in genomic deletions. *Nucleic Acids Research* https://doi.org/10.1093/nar/gky691 (2018) doi:10.1093/nar/gky691.

Table 1: Patient Characteristics

Variables	*preeclampsia Cases (n = 24) mean (sd)*	*Controls (n = 38) mean (sd)*	*P-value*
Maternal Age (Years)	*28.75 (5.88)*	*27.24 (6.35)*	*0.40*
Parity	*1.54 (1.41)*	*1.57 (1.78)*	*0.77*
BMI	*32.24 (9.38)*	*27.75 (9.20)*	*0.07*
Smoker (n)	*6*	*8*	*0.96*
Gestational Age (Weeks)	*35.58 (2.90)*	*39.16 (0.92)*	*2.485e-07*
Ethnicity (n)			*0.60*
Asian	*12*	*21*	-
European Ancestry	*3*	*7*	-
Pacific Islander	*9*	*10*	-

* *Numeric variables are compared with the Wilcoxon test;*
* *Categorical variables are compared with the Chi-square test.*

Figure 1: Study Overview and Experiment Design. The entire data analysis procedure is outlined in this workflow, which incorporates methods that account for clinical variables and cell-type heterogeneity. EWAS: Epigenetic-wide association study.

Figure 2: Cell types in samples. (A) The Source of Variance (SOV) analysis of cell type composition from patient characteristics. Major contributors of DNA methylation variation were identified as the factors (x-axis) with an F-mean value greater than 1.(B) Side-by-side boxplots displaying cell-type proportions in preeclampsia cases vs. controls before adjusting for clinical variables. An asterisk (*) is used to indicate a statistically significant difference by using Multiple Linear Regression (MLR) between the case and control groups (p-value < 0.05), while "ns" is used to indicate a non-statistically significant difference. (C) Side-by-side boxplots displaying cell-type proportions in preeclampsia cases vs. controls with p-values from multiple linear regression of cell proportion on preeclampsia and all major factors for methylation-variation in (A).

Figure 3: Preeclampsia is not associated with statistically significant changes in cord blood DNA methylation, after clinical variables, gestational age and cell proportions adjustment. Result generated using data from HiBR cohort (A-C), Ching T et. al (D-F), Herzog EM et al (G-I) and pooled analysis combining the three cohorts (J-L). (A, D, G) The Source of Variance (SOV) analyses were conducted on cell types and clinical variables whenever available, to verify the proportion of DNA methylation variance attributable to these factors. (B, E, H, K) The volcano

plot of the differential methylation analysis results without adjustment. The x-axis represents log fold change between severe preeclampsia and controls; the y-axis is negative log-transformed p-values after Benjamini-Hochberg (BH) adjustment. The red dots are differentially methylated probes (DMP) associated with severe preeclampsia after BH adjustment, whereas the black dots represent non-statistically significant probes. (C, F, I, L) The volcano plot after adjusting for clinical variables, gestational age and estimated cell proportions. (J) The cell proportion distribution in the combined 3 datasets, separated by sample groups. Gran: granulocyte, NK: natural killer cell, B cell: B-lymphocyte, Mono: monocytes, nRBC: nucleated red blood cell, CD4T:

Figure 4: Cell type proportions in relationship to the gestational age. Scatter plots (A) depict the proportions of each cell type in cord blood from all samples, along with gestational age. The reported beta and p-value measures the relationship between gestational age and each cell type, with a threshold of p-value < 0.05. (B) Scatter plot of estimated cell proportions from merged HiBR data and those from Fernando et al., by gestational age. Beta and p-value refer to the coefficient and p-value of the interaction between preeclampsia and gestational age in each cell type.

Figure 5: Cell proportions in relation to gestational age are coherent in two different datasets. The scatter plots compare the cell-type proportions with gestational age in our data and Kashima et al. Plots (A) display the comparisons within preeclampsia case samples for both studies, whereas plots (B) display the comparisons within control samples for both studies. The purple line in each plot represents the fitted cell proportions in our whole cord blood samples, while the orange line represents the fitted cell proportions in cord blood mononuclear cell (CBMC) cord blood samples from Kashima K et al., another study. We test whether the cell proportion changes in the two datasets, using CBMC vs. the whole blood (WB), are statistically significantly different by linearly regressing cell proportions over gestational age, datasets, and the interaction between the datasets and gestational age. We report the p-values of such interaction terms. A non-statistically significant p-value indicates the cell proportion trends are not statistically different in the two datasets.

Figure1

Click here to access/download;Figure;Copy of CB_DNAm_PE_Workflow_V4 (1).png

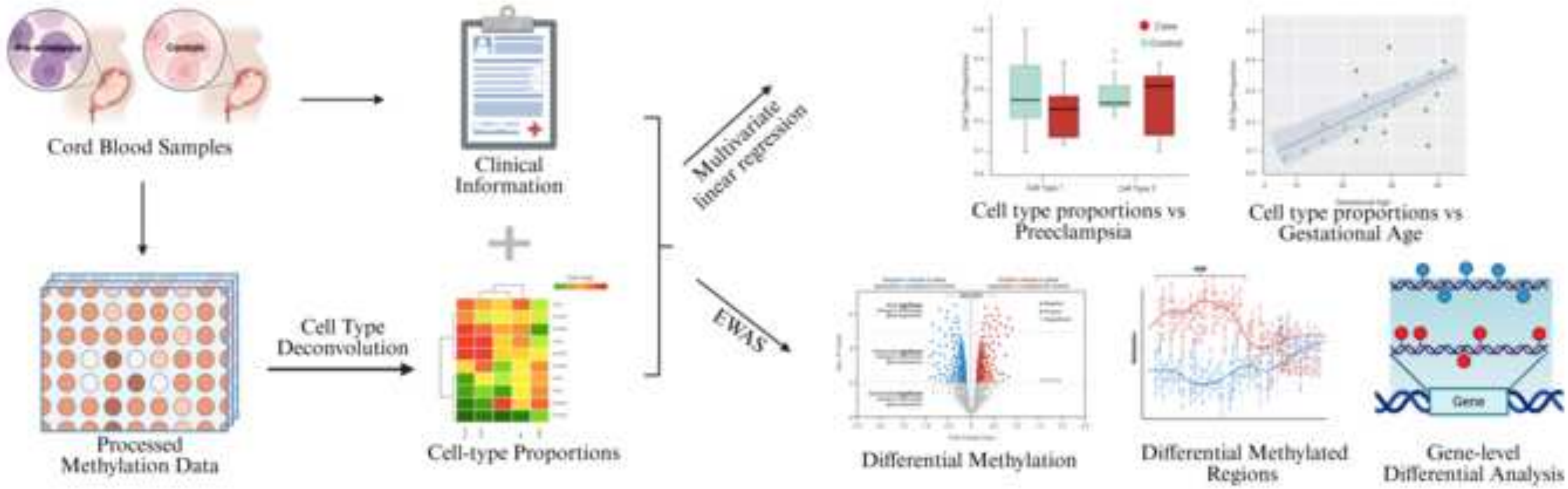

Click here to access/download;Figure;figure2.tif

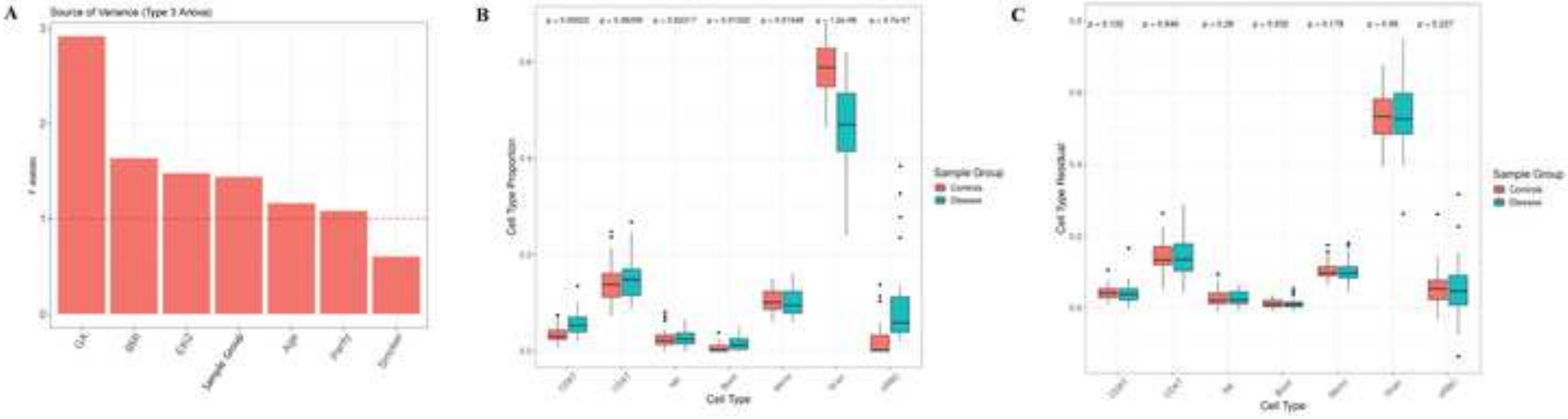

Figure3

Click here to access/download;Figure;figure3.png

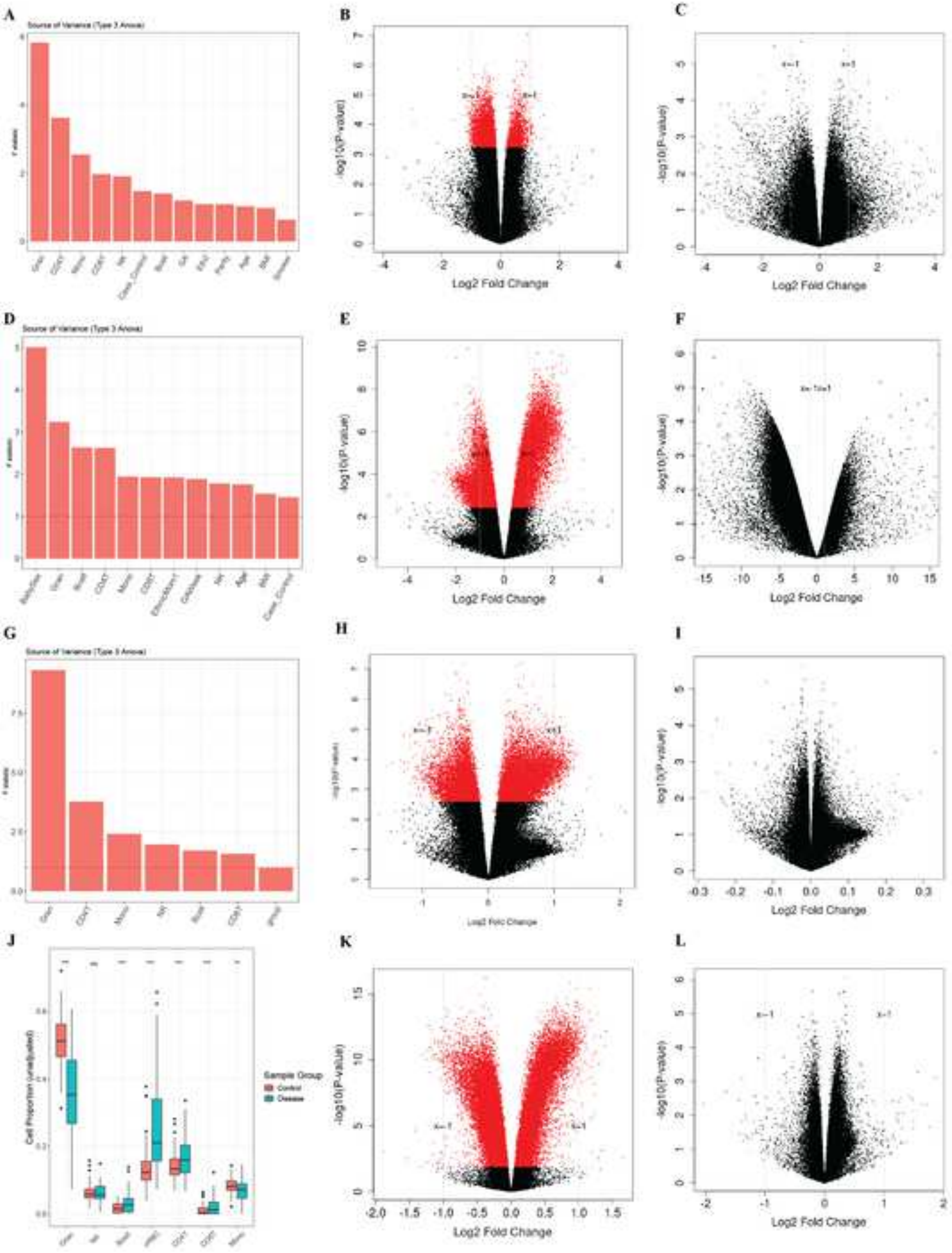

Click here to access/download;Figure;figure4.png

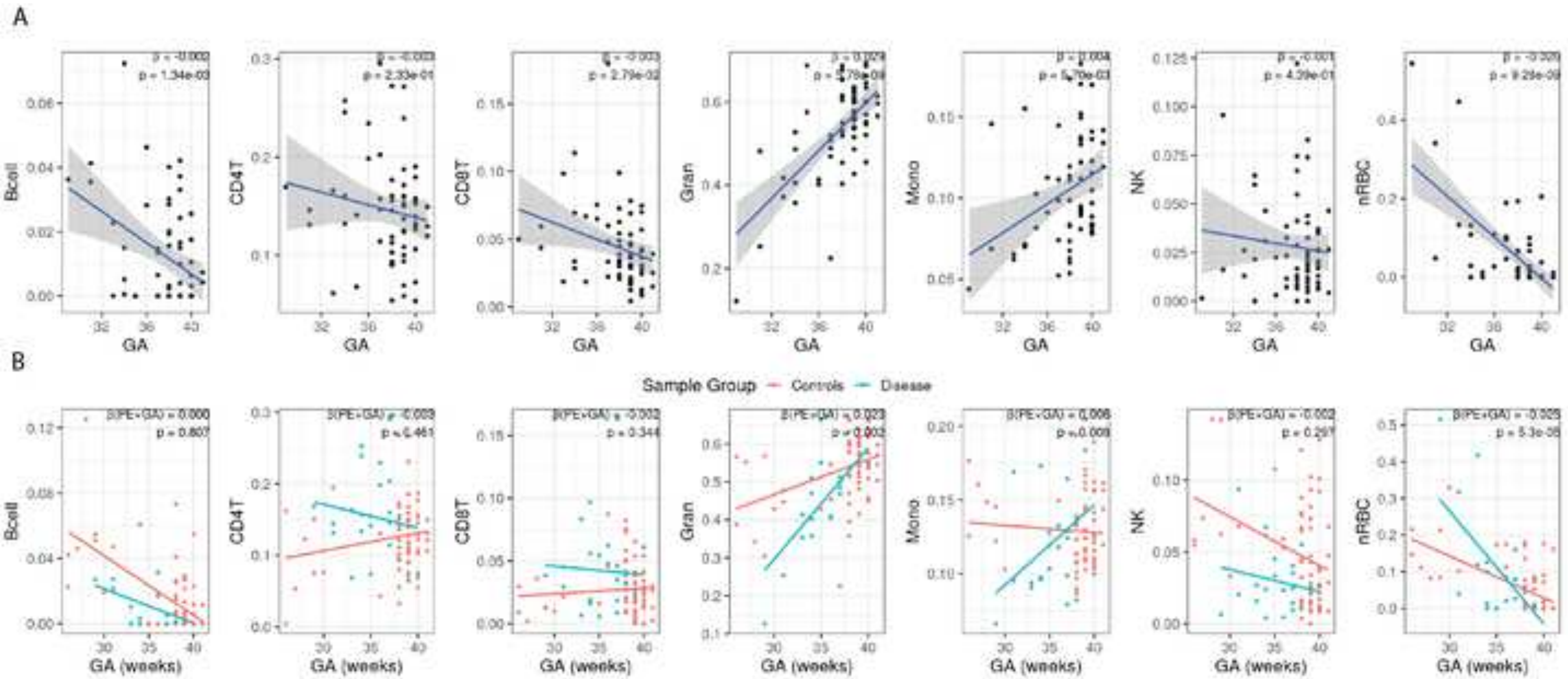

Figure5

Click here to access/download;Figure;figure5.png

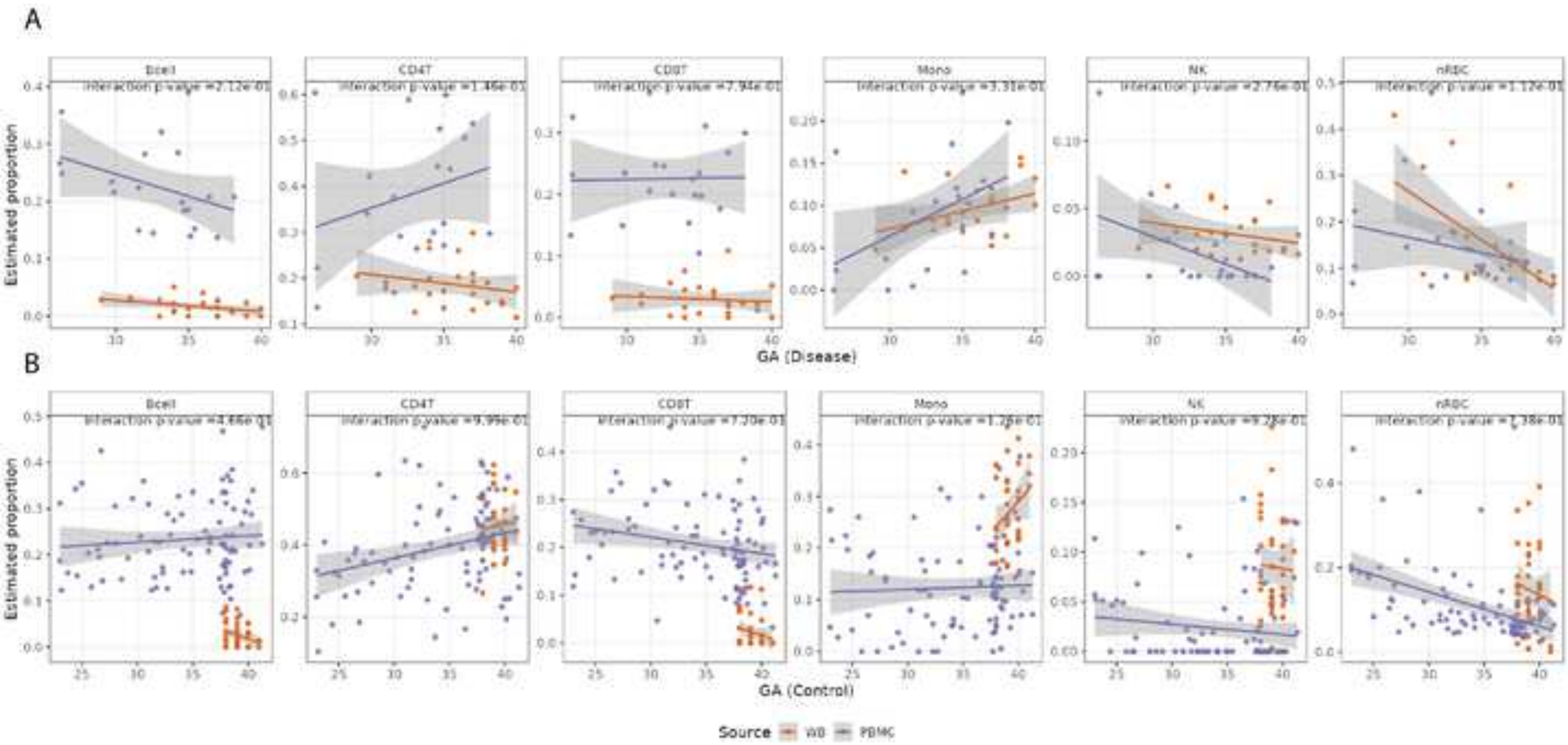

Click here to access/download
Supplementary Material
Supplementary_material.docx

Personal Cover
Click here to access/download;Personal Cover;PE_CordBlood_EWAS_CoverLetter.docx

MICHIGAN MEDICINE
UNIVERSITY OF MICHIGAN

University of Michigan Medical School
Department of Computational Medicine & Bioinformatics

11/11/2025

Dear Editors,

We are pleased to submit the revised version of our manuscript, titled *"Cell type proportions rather than DNA methylation in the cord blood show significant associations with severe preeclampsia,"* for consideration by **GigaScience**.

We are deeply grateful to the reviewers and editors for their thoughtful and constructive feedback, which has greatly improved the clarity and rigor of our work. We have carefully revised the manuscript in response to all comments and invested substantial effort in reanalyzing data, refining interpretations, and strengthening the presentation.

In this work, we showed that cell-type proportions estimated through deconvolution vary greatly among subjects and are the most significant confounders. After adjusting cell type proportions and patient clinical characteristics, there is a lack of significant CpG methylation changes in the EWAS study associated with severe preeclampsia in all datasets available publicly, either individually or combined all together. We therefore conclude therefore that severe maternal preeclampsia does not affect DNA methylation in the cord blood cells significantly.

We believe this work aligns well with **GigaScience's** mission to promote rigorous, transparent, and reproducible data science research.

Please feel free to email me if you have any questions regarding this submission. lgarmire@umich.edu

Sincerely,

Lana Garmire, PhD
Associate Professor
Department of Computational Medicine and Bioinformatics
Medical School, University of Michigan, Ann Arbor, 48105, USA

Reviewer #1:

Credentials
I am an Associate Professor of Epidemiology and Health Equity at the Department of Public Health at the University of Copenhagen. I have been reviewing articles for over a decade for >30 biomedical journals, and I have been a key reviewer and statistical reviewer at the journal Diabetologia every year since 2020, with around 10 reviewed manuscripts each year for the past couple of years, with excellent editorial feedback. I obtained Clarivate Web of Science Academy certificates in Scientific Peer Review. My expertise is in cardiometabolic disease epidemiology, genetic epidemiology, biostatistics, machine learning, artificial intelligence, algorithmic fairness, and health inequalities. I have significant experience authoring and reviewing papers related to various omics studies, including genomics, epigenomics, and lipidomics. I am familiar with epigenetic association analysis pipelines and have reviewed, e.g., several papers related to epigenomics clocks. I also have the
necessary expertise to assess the study design and the statistical methodology of this paper. In fact, I have analyzed omics data using similar pipelines, and am familiar with some of the bioinformatics toolkits utilized in this paper (e.g., limma, pathway analysis tools). No AI technologies were used in the writing of this review other than a standard spell checker to correct typos.

Overall

I read the study by Yang X. et al. with interest. I was particularly interested in this study due to its dual aims: I really liked that the study had a strong biological hypothesis, but also aimed to highlight the importance of methodological considerations related to confounder adjustment. Based on my assessment, I see the second aim (statistical) fully delivered, but the first aim (biological/causal) potentially misleading or overstated. I fully agree with the authors in their final message that all studies should pay attention to proper confounder adjustment, including clinical variables and also cell proportions, when it comes to epigenomics analysis. However, the authors themselves fell into a trap of getting a bit lost in adjustments and not properly considering confounders. First, it appears that confounder selection was automated based on statistical relationships, which shows the lack of thought put into appropriately selecting these based on a priori hypotheses
(e.g., via drawing up a DAG). Second, the results are often misinterpreted. The authors claim a causal relationship (perhaps via poorly chosen wording) in the Discussion, and seem to conclude that cell proportions are key drivers in this study, whereas to me it appears, based on the results, that gestational age is the strongest correlate with DNA methylation patterns, rather than cell proportions. In my opinion, the following key findings emerged here, and this would be a logical order for presentation:1

1) Preeclampsia strongly associates with gestational age (known);

2) Cell proportions strongly correlate with gestational age (known);

3) CD8T cell proportions associate with preeclampsia even after gestational age adjustment (main effect, key novel finding);

4) Monocyte cell proportions show differential association with gestational age according to preeclampsia status (interaction, key novel finding).

Thank you for your suggestions. Our initial main motivation to submit this work, is to alert the readers the importance of adjusting for different cell types and clinical covariates (eg. GA) in the case study of preeclampsia. It was the surprising negative finding of lacking sufficient DNA methylation changes in association with severe preeclampsia after adjust these factors, which prompted us to share this story to the community. We figured that if we as bioinformaticians, had done the analysis inappropriately before, we should correct ourselves and let the community know how to do it properly. Thus, we think we should present the most significant negative results regarding the lack of association between DNA methylation changes and preeclampsia first. We then report other less striking but still important findings on cell proportions in association with gestational age next.

Thanks to the reminder of multiple hypothesis test, the CD8T cell proportion association with preeclampsia is not significant anymore, after gestational age adjustment.

Then, I would suggest the authors dive deeper into a "causal ideation": yes, DNA methylation associations disappear after adjustment for gestational age (and perhaps cell proportions), but this does not mean that they are not important. In fact, they can still be causal drivers of outcomes! But a more in-depth exploration into what comes first: the disease, the methylation, altered cell proportions, and what impacts what, would be a real added value to the Discussion of this paper. Please see my detailed comments below, hope they are helpful.

Introduction

- I suggest that you do not abbreviate preeclampsia as PE. It does not change the word count, and it is easier to read the full word out and avoid an unnecessary abbreviation.

Thank you for the suggestion. We have replaced all “PE” with “preeclampsia” in the manuscript.

- You mention that the Kazmi et al. article reported certain findings "after adjusting for cell types". Does this mean cell proportions, or how does this adjustment normally happen?

Yes, “adjusting for cell types” in Kazmi et al. means adjusting for estimated cell proportions. We included estimated cell proportions as variables in the linear regression model for differential DNA methylation analysis.

To avoid confusion, we changed “adjusting for cell type” to “adjusting for estimated cell proportion”.

- "(Kazmi et al) However, their results did not adjust for the gestational age, the major confounder of preeclampsia" - This sentence needs expansion. A confounder between what two factors? Can you explain how gestational age is a confounder in this setting (with respect to the key statistical criteria for being a confounder)?

Thank you for pointing this out. Our original wording was incorrect. Gestational age is a mediator not a confounder for DNA methylation outcome. We rephrased it to: “However, they did not adjust for gestational age, which is strongly associated with both preeclampsia and DNA methylation, and therefore should be included as a key covariate.”.

In this setting, gestational age (GA) at delivery is strongly associated with the exposure (preeclampsia) and also influences the outcome (cord-blood DNA methylation). The assumed causal pathway should look like:

PE → GA→ DNA methtylkation

Thus, GA is better described as a mediator rather than a confounder.

Still EWAS studies adjust for GA, in order to isolate methylation changes that are directly attributable to preeclampsia and independent of gestational age at the time of delivery [1,2]. In other words, we should understand in the epigenome changes due to preeclampsia itself, rather than these changes accompanying gestational progression.

We added clarifications and explanations for this claim.

[1] Herzog EM, Eggink AJ, van der Zee M, Lagendijk J, Willemsen SP, de Jonge R, Steegers EA, Steegers-Theunissen RP. The impact of early- and late-onset preeclampsia on umbilical cord blood cell populations. J Reprod Immunol. 2016 Aug;116:81-5. doi: 10.1016/j.jri.2016.05.002. Epub 2016 May 11. PMID: 27239988.
[2] Knihtilä HM, Kachroo P, Shadid I, Raissadati A, Peng C, McElrath TF, Litonjua AA, Demeo DL, Loscalzo J, Weiss ST, Mirzakhani H. Cord blood DNA methylation signatures associated with preeclampsia are enriched for cardiovascular pathways: insights from the VDAART trial. EBioMedicine. 2023 Dec;98:104890. doi: 10.1016/j.ebiom.2023.104890. Epub 2023 Nov 22. PMID: 37995466; PMCID: PMC10709000.

- The Introduction is excellent in clearly outlining a problem statement of poor confounder adjustment in epidemiological studies. I fully agree with the authors that this is often ignored in these studies. Key references are listed, showing a pattern of ignoring key confounders.

Thank you for your agreement

- Perhaps some more information in the Introduction on source tissues would be helpful to the reader. Are all the referred studies analyzed cord blood or rather maternal blood? What are the expected differences in results given tissue sources?

Thanks for your insight.

In the introduction section, we first mentioned studies [6-12], conducted on placentas [7-9, 11,12] and maternal blood[6,10]. After introducing the intergenerational effect of preeclampsia, we moved on overviewing studies on cord blood tissues[16, 18-20] and cord tissues [17].

We have specified the tissue origin of mentioned studies in the introduction. We also added a sentence to explain the expected difference between tissue types.

- "It is therefore essential to account for such heterogeneity, to improve the accuracy and sensitivity, and avoid biased conclusions" - Accuracy and sensitivity of what? From your problem statement and Introduction, my understanding is that this study relates to causal/biological discovery and highlighting methodological limitations (re: confounder adjustment). Accuracy and sensitivity are terms related to predictive statistics that have not been mentioned before, so these terms feel out of place. Besides, I do not think your study design is helpful for prediction.

Thank you for flagging this error. Indeed, “accuracy” and “sensitivity” are metrics used to evaluate prediction problems, and our study(EWAS) does not involve prediction tasks. Our intention was to emphasize robustness and validity. We will replace “accuracy” and sensitivity with “robustness and validity” to avoid confusion.

- "Particularly, to ensure that any differences in DNA methylation are due to confounding factors, the analysis needs to be adjusted for cell proportions" - I think you meant to say that "to ensure that differences in DNA methylation are NOT due to confounding factors", right?

Thank you for pointing this out. We have fixed it in the manuscript.

- "In this study, we pay special attention to these issues to seek a plausible epigenomic association between severe PE and cord blood of offspring from PE patients." - This reads a bit clumsily, and wording should be changed to more accurately describe the goals of your study. Associations are not sought after "between PE and cord blood", but PE and various epigenetic and cellular markers IN cord blood.

We have rewritten this sentence following your suggestion: "In this study, we specifically address these issues to seek potential epigenomic and cellular markers in cord blood associated with severe preeclampsia."

- I am surprised that eclampsia is not mentioned in the Introduction as an even more severe condition. Is there any evidence related to the associations between epigenetic markers and cellular markers and eclampsia?

We thank the reviewer for this thoughtful comment. We now mention eclampsia as one of the most severe manifestations on the preeclampsia spectrum. However, the prevalence is very low (0.1–0.3% vs. ~3% for preeclampsia). Possibly due to this reason, in PubMed we did not find epigenome-wide association studies specifically on eclampsia.

Methods

- Please write out "OBGYNs" in full words at first mention.

We wrote out the full words for OBGYN (obstetricians and gynecologists) in the method section.

- "Severe PE was characterized by OBGYNs at Kapiolani Medical Center as sustained pregnancy induced hypertension(systolic/diastolic blood pressure >= 140/90) with urine protein and/or organ dysfunction." - First, what do you mean by organ dysfunction? Which organ? Second, my read is that both elevated BP and proteinuria are needed for this diagnosis, but organ dysfunction is optional. Or is it that either "elevated BP-proteinuria" or "elevated BP-organ dysfunction" combinations are satisfactory? Please clarify.

We apologize for the confusion. The definition we originally included corresponds to preeclampsia, whereas for severe preeclampsia the criteria we used were: among patients with preeclampsia (blood pressure ≥140/90 mmHg with proteinuria), those who additionally present with either (i) severe-range hypertension (blood pressure ≥160/110 mmHg), (ii) severe proteinuria (≥5 g in a 24-hour urine specimen or ≥3+ on two random urine samples collected at least 4 hours apart), or (iii) evidence of organ dysfunction(see below). Since the samples were collected before the new ACOG guidelines for preeclampsia diagnosis was released, the diagnosis criteria followed the older ACOG guidelines[1].

By organ dysfunction, we refer to the standard ACOG severe features, including thrombocytopenia (low platelet count), impaired liver function (elevated liver transaminases), progressive renal insufficiency (elevated creatinine or reduced urine output), pulmonary edema, or new-onset cerebral or visual disturbances.

In conclusion, “blood pressure ≥140/90 mmHg with proteinuria” and one or more of the three additional severe features are needed to diagnose severe PE. We have revised the Methods section to accurately reflect these diagnostic criteria.

[1] ACOG Committee on Practice Bulletins--Obstetrics. ACOG practice bulletin. Diagnosis and management of preeclampsia and eclampsia. Number 33, January 2002. Obstet Gynecol. 2002 Jan;99(1):159-67. doi: 10.1016/s0029-7844(01)01747-1. PMID: 16175681.

- "…and had cord blood samples remaining in the HiBR" - So your inclusion criterion is that there are samples remaining. Can this selection process introduce selection bias? I.e., for what reasons are cord blood samples removed from HiBR? If this is related to certain outcomes or baseline characteristics of the participants, then there is a selection mechanism that should be acknowledged, and the process's impact should be considered.

Thank you for raising this. Our inclusion criterion “samples remaining in HiBR” refers only to residual aliquot availability (i.e., not yet exhausted). Aliquot depletion in HiBR occurs for operational reasons: prior use by other projects, limited initial volume, or QC issues (e.g., hemolysis/low yield), rather than participant outcomes.

To ensure the samples are unbiased, we matched the case and controls by age, ethnicity, and pre-pregnancy BMI. As you can see in Table 1, patient characteristics between the case and control samples are not statistically different except for gestational age, which is on average shorter in the case group by clinical guidelines.

- "We evaluated sample integrity, purity, and concentration on the Nanodrop and removed samples of low quality." - What is the Nanodrop? How was low quality defined, and according to what metrics?

We thank the reviewer for this question. The NanoDrop (Thermo Fisher Scientific) is a microvolume UV–Vis spectrophotometer commonly used to assess nucleic acid samples. It measures absorbance at 260 nm to estimate DNA concentration, and the 260/280 nm and 260/230 nm absorbance ratios are used as indicators of purity. According to the manufacturer’s guidelines, a 260/280 ratio of ~1.8 is generally accepted as “pure” for DNA, while lower ratios may indicate protein contamination. Similarly, a 260/230 ratio close to 2.0 is considered optimal, with lower values suggesting contamination by salts, phenol, or other organics. These criteria are consistent with the NanoDrop user manual recommendations.

We have added a sentence to explain the quality threshold in the method section under the “The Hawaii Biorepository (HiBR) cohort” subsection.

- It would be very useful to present a directed acyclic graph (DAG) or a causal loop diagram (CLD) at some point in the manuscript. This could already happen in the Introduction stage, where you present the problem statement and your scientific hypothesis. This could formalize your hypothesis and also help you ascertain the confounder structure around the investigated statistical hypotheses. For some of the variables you chose to adjust for, I cannot immediately see whether they are potential confounders or mediators/colliders (in which case you should not adjust), so I suggest that you visualize everything with the DAG, and that you actually write out the three criteria for being a confounder in the Methods for the reader. I know that this is basic information, but this is key information for your manuscript that should be well understood by every reader of your paper to be able to fully appreciate the results (and who knows, you might uncover some new information through the visualization that you have not considered?).

Thank you for this helpful suggestion. We have added a directed acyclic graph (DAG) to the manuscript to formalize our study assumptions (supplementary figure 3) and clarify the relationships among variables. We did not use a causal loop diagram (CLD), as our associations are not cyclic. As shown in the figure, preeclampsia is treated as the exposure and DNA methylation is the outcome. Clinical variables (age, BMI, ethnicity, smoking, parity) are associated with both exposure and outcome and are not on causal pathway, so they are considered as upstream confounders. GA and cell proportion are on the causal pathways in the graph, they may mediate the observed DNA methylation changes and thus need to be adjusted, in order to tease out the actual direct association of preeclampsia to DNA methylation changes.

In EWAS study, researchers look for the cell-intrinsic alterations in dna methylation profile, which is independent of cell proportion and clinical mediators (eg. GA) of the sample tissue. It is standard practice in EWAS to adjust for estimated cell-type proportions (and related factors such as gestational age) to reveal the direct effect of disease on DNA methylation, independent of cell-mixture variation (Gervin et al., Clin Epigenet 2019 [22]; Zheng et al., Nat Methods 2018 [53]; Qi & Teschendorff, Clin Epigenet 2022 [55]; Merid et al., Genome Med 2020 [57]).

We also describe in the Methods section the three standard criteria for identifying confounders, as suggested.

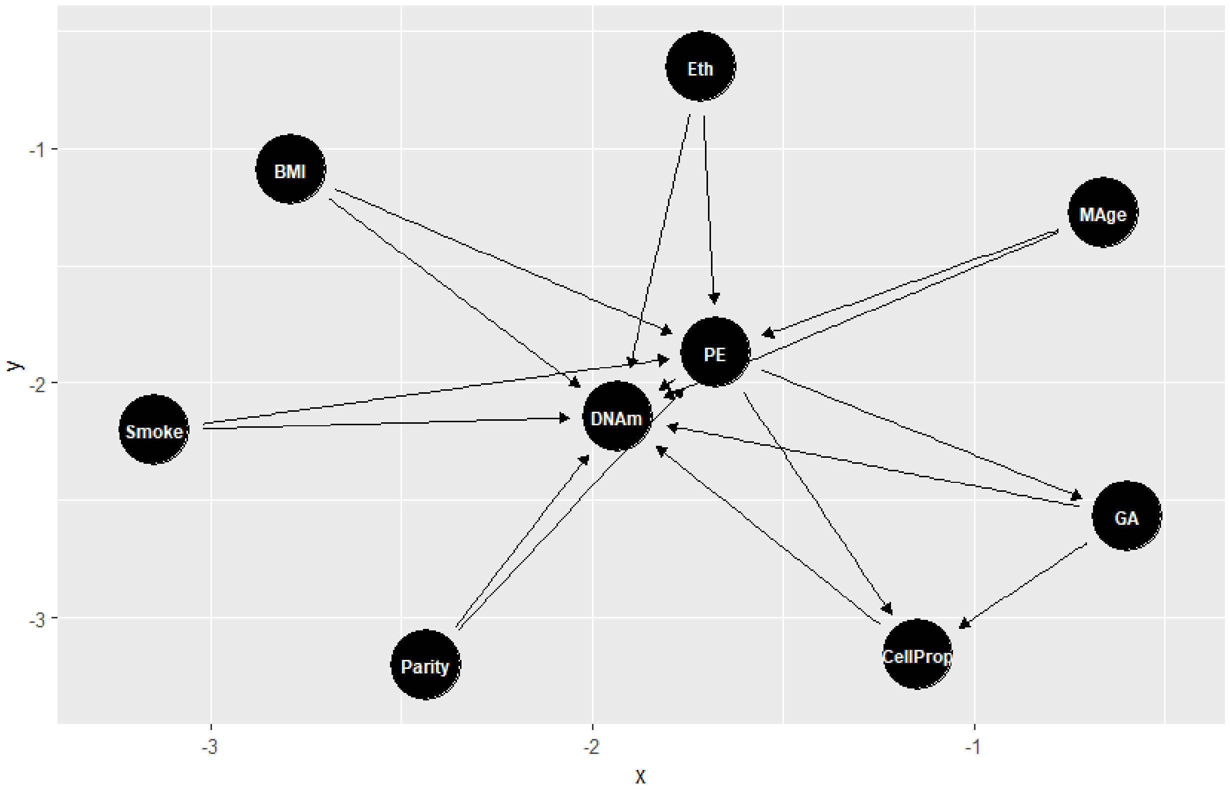

- "The demographic and clinical information of the patients was collected and analyzed to identify any potential confounding effects." - What do you mean by this sentence? How was this done?

We appreciate the request for clarification. We rephrased this to “The demographic and clinical information of the patients was collected and analyzed to identify any potential association with DNA methylation”.

We collected demographic and clinical variables that are commonly reported to influence DNA methylation in cord blood (including maternal age, pre-pregnancy BMI, parity, smoking status, ethnicity, and gestational age at delivery). To evaluate their potential association with DNA methylation, we first included them in a source of variance (SOV) analysis, where each CpG site was regressed on these variables and F-statistics were averaged across sites. Variables with an average F-statistic greater than 1 (i.e., explaining more variance than noise) were retained as covariates and included in the downstream linear models for both CpG-level and region-level methylation analyses.

However, as you and other reviewers pointed out, a statistical test like SOV shouldn’t be used to identify confounders, because confounders are determined by relationships with exposure and outcome. We now only use SOV as a way to illustrate the contributors of DNA methylation changes. We clarified this change in both Methods and Results section.

- "To validate the observations in the HiBR cohort, we did an exhaustive search among published work of cord blood (including PBMC) DNA methylation in association with PE (16,17,23)" - I suggest that you include a brief supplementary document on how you undertook this "exhaustive search". What does this term mean? Is this a systematic review? Did you establish a search term or involve a librarian? You refer to three papers, but it is unclear how these articles were identified.

We thank the reviewer for this comment and agree that our wording was imprecise. By “exhaustive search,” we meant that we searched PubMed for all available studies on preeclampsia and cord blood DNA methylation at the time of our analysis, and included all results we found.

To avoid confusion, we have revised the text: *“We searched PubMed for published studies of preeclampsia and cord blood DNA methylation and included all datasets identified (Ching et al., Herzog et al., and Kashima et al.).”*

- "baby gender, and baby birth weight" - While, of course, this is understandable, I am wondering if "baby" is scientifically appropriate. I suggest replacing this with "infant".
Thank you for pointing this out. We have changed all “baby” in the manuscript to “infant. We kept the ‘babysex’ term in figure

- "including 10 early-onsets and 13 late-onsets" - You previously introduced abbreviations for these: EOPE, LOPE. I suggest you either stick to the abbreviations or remove the abbreviations altogether.

Thank you for flagging this! We have changed “early-onsets” and “late-onsets” to “EOPE” and “LOPE” to maintain consistency.

- "For Herzog EM et al and Kashima K et al datasets, we directly used the beta matrix deposited to GEO." - Please explain what the "beta matrix" is (I am assuming these are the summary level effect estimates), and what GEO stands for.

The “beta matrix” is the processed DNA-methylation data: each entry is a β-value between 0 and 1, representing the proportion of methylation at a given CpG site in a given sample. Each row corresponds to a CpG probe and each column corresponds to an individual sample. It’s not summarised but transformed directly from the raw intensities.

GEO refers to the Gene Expression Omnibus, the NCBI-hosted public repository where high-throughput functional genomics datasets (including methylation arrays) are deposited and freely accessible.

We have added an explanation of “beta-value matrix” to the manuscript and the full name of GEO.

- "To disentangle the effect of PE and small GA" - Please refer to this as associations, as effect implies causality, which cannot be demonstrated by these results. In general, please be mindful of this throughout the manuscript and refer to all observed results as associations, and not effects. Even for betas, I would prefer "magnitude of association", rather than "effect size".

Thank you, this is an important suggestion. We have replaced the word “effect” with association to avoid misinterpretation for causal effect, except for fixed terms like “batch effect” or “confounding effect”.

- "idiopathic preterm birth samples" - What does idiopathic mean? Please explain for the general readership.

Certainly, "idiopathic preterm birth samples" refers to samples from cases of preterm birth where the cause is not known. According to Fernando et al., idiopathic preterm delivery initiated by either spontaneous preterm labor (PtLb) with intact membranes or preterm premature rupture of membranes (PPROM)[1]. We have added the explanation in the abstract and method section when we introduce the Fernando dataset.

[1]Fernando F, Keijser R, Henneman P, van der Kevie-Kersemaekers AM, Mannens MM, van der Post JA, Afink GB, Ris-Stalpers C. The idiopathic preterm delivery methylation profile in umbilical cord blood DNA. BMC Genomics. 2015 Sep 29;16:736. doi: 10.1186/s12864-015-1915-4. PMID: 26419829; PMCID: PMC4588235.

- For Fernando et al, you report: "We directly used the normalized matrix deposited to GEO." - Above, for Herzog and Kashima, you just referred to this as the "beta matrix". Is there a difference between the two (i.e., one is normalized, the other one not)?

Yes, the uploaded data in Fernando et al. is the normalized version of the aforementioned beta matrix. Usually, normalization is the first step to analyze a DNA methylation beta matrix.

We have changed the phrase to “normalized beta matrix” to avoid confusion.

- At first mention, please write out the full names of CD4T and CD8T cells.

Thank you, we have changed the abbreviation of cd4t, cd8t and b cell to their full name at the first mention.

- For all potential confounders listed under "Clinical confounders and source of variance analysis", please list categories and precise definitions. E.g., is BMI pre-pregnancy BMI (and when was it measured)? How is smoking status defined (is it during pregnancy, before pregnancy, what are the categories)? What are the categories for ethnicity?

We thank the reviewer for this helpful comment. In the revised manuscript, we now provide precise definitions and categories for all clinical confounders. Specifically, BMI refers to pre-pregnancy BMI measured at the first prenatal visit. Smoking status was defined as maternal self-reported smoking during pregnancy (yes/no). Ethnicity categories included Asian, Pacific Islander, Caucasian, and Other, as recorded in the medical record.

We have updated the Methods to clarify these definitions.

- "We performed the source of variance (SOV) analysis on these clinical variables and previously estimated sample cell proportions to identify important confounding variables that need to be adjusted, as done before." - Despite the inclusion of three references at the end of this sentence, this needs clarification. You explain a statistical process after this sentence, implying that confounders were selected purely based on statistical relationships. This is not appropriate. Confounders should be selected based on a priori assumptions about whether something is a confounder or not. The three criteria for being a confounder are: 1) The variable must be statistically associated with the exposure. 2) The variable must cause the outcome. 3) The variable must not be on a causal pathway. As suggested above, I would much prefer that you visualize your investigated association using a DAG and determine confounders based on that exploration.

Thank you this valuable comment. We agree that a priori biological and epidemiological reasoning is more appropriate than relying solely on statistical associations. In the revised manuscript, we now clarify that we first collected demographic and clinical variables commonly reported to influence cord blood DNA methylation (maternal age, pre-pregnancy BMI, parity, smoking status, ethnicity, and gestational age at delivery). These were treated as potential confounders based on prior knowledge. We then applied SOV analysis only as a confirmation

step to assess their relative contribution to variation in methylation data, but we did not exclude any potential confounders based on the SOV results.

Additionally, we have included a DAG in the supplementary materials to make our assumptions about confounder relationships explicit.

- "The p-value of PE was adjusted with Benjamini-Hochberg (BH) adjustment (threshold of 0.05)." - This is appropriate and established in the literature. I suggest that you clarify that the threshold is an alpha type 1 error threshold.

Thank you, we have added the clarification that 0.05 is the type I error threshold.

- "We included study participants' GA (GA)," - There is a double abbreviation here, maybe this is a typo?

Yes, we apologize for the typo. We have changed the first GA to its full name, gestational age.

- Please do not use "significant(ly)" as a standalone term. Rather, please use "statistical significance" or "clinical significance", depending on the context. The term has been massively overused in biomedical literature, with a generally poor understanding among the readership.

Thank you for this important suggestion. Indeed, it’s easy to misunderstand the term “significance”. We have changed all “significant/significance” that refers to a difference that exceeds the threshold (type I error) in the text to “statistically significant/ statistical significance.”

- "We did not include data from Kashimi et al. because there was no significant cpg before the confounder adjustment." - I do not understand this justification, and this is potentially a flawed approach. My assessment is that all samples, regardless of the statistical significance of original associations (with or without adjustment), should be included in the meta-analysis. Otherwise, you will obtain biased estimates.

We apologize for the confusion in our original wording. We agree with the reviewer that excluding a dataset based on statistical significance would be a flawed approach and could bias results.

Our actual reason for not including the Kashima et al. dataset was that it was generated from cord blood PBMCs, whereas all other included datasets (our in-house cohort, Ching et al., and Herzog et al.) were based on whole cord blood. Because PBMCs lack granulocytes and therefore have a substantially different cell composition, including them would not be appropriate.

We revised this sentence accordingly.

- "To harmonize the datasets, we applied the combat function to remove batch effects while preserving sample group information" - Please correct this to "ComBat". There are further mentions of this in the manuscript, please double-check consistent naming.

Thank you, we have changed all “combat” to “ComBat” throughout the manuscript.

- "resulting in an inevitable correlation of PE cases and smaller gestational age." - To make this more precise, I would say that there is a positive correlation between PE status and smaller gestational age.

Changed the original sentences to "resulting in an inevitable positive correlation of PE cases and smaller gestational age." as suggested.

- As a very minor comment, sometimes you write out gestational age, sometimes you write GA. Please be consistent (I suggest writing it out).

Thank you, we have changed all GA in the manuscript back to gestational age, to ensure consistency.

- What is the reason for applying FWER correction in one part of your analytic pipeline and B-H FDR in another part? Is there a specific reason for correcting for false positive rates vs. false discovery rates in the two analytic processes?

FWER appears only in our DMR analysis because the bumphunter algorithm outputs permutation-based, FWER-controlled p-values by design; this is not user-configurable. For DNA methylation analysis, we control multiplicity with Benjamini–Hochberg FDR, which is standard practice in epigenetic-wide association study(EWAS) and used by prior cord-blood methylation studies [1, 2]. Using FDR at the probe level ensures fair comparison of p-values across datasets and with the literature.

[1] Ching T, Ha J, Song MA, et al. Genome-scale hypomethylation in the cord blood DNAs associated with early onset preeclampsia. Clinical Epigenetics. 2015;7:21. DOI: 10.1186/s13148-015-0052-x. PMID: 25806090; PMCID: PMC4371797.

[2] Knihtilä HM, Kachroo P, Shadid I, Raissadati A, Peng C, McElrath TF, Litonjua AA, Demeo DL, Loscalzo J, Weiss ST, Mirzakhani H. Cord blood DNA methylation signatures associated with preeclampsia are enriched for cardiovascular pathways: insights from the VDAART trial. EBioMedicine. 2023 Dec;98:104890. doi: 10.1016/j.ebiom.2023.104890. Epub 2023 Nov 22. PMID: 37995466; PMCID: PMC10709000.

- It is very commendable that the authors made their code publicly available, amazing!

Thank you for the supportive comment.

Results

- "There is no significant difference ($P > 0.05$) in maternal age, parity, BMI, ethnicity, and smoking status." - Difference between what groups? Please be explicit.

We are referring to the difference between PE cases and controls, the detailed statistics(mean, standard deviation, p-values) of these variables are included in table 1.

We added the clarification in the text "Maternal characteristics were similar between the preeclampsia and control groups, except cases had significantly earlier gestational age at delivery ($P = 3.66\times10^{-6}$) (Table 1)"

- There is quite a bit of repetition in the Results related to key methodologies that were already explained in detail under Methods. These could be removed to shorten the Results section (e.g., around 2/3 of the first paragraph of the Results is repetition, referring to the same supplemental material files that you already referred to in the Methods).
Thank you for the suggestion. We've shortened the first paragraph of the Results section and kept only the key details, so that readers who skip the Methods can still follow the results.

- Where you present cell proportion differences between PE and control, you indicate "t" for some cell types, and "beta" for other cell types. What motivates this inconsistency? What do these represent? Is the scale here % difference between the two groups (if so, please state the unit explicitly).
Apologies for the typo—these should all be "t," referring to the t-statistics from two-sided t-tests. We used two-sided t-tests to compare the distribution of each cell type's proportion between cases and controls. All values are on the same scale: cell proportions expressed as a value between 0 to 1. They don't have a unit because they are a proportion.
We have revised the typo in the manuscript.

- "Since previous studies reported that maternal smoking significantly affects DNA methylation (43)." - This is not a full sentence, please combine it with the next one.
Thanks for flagging this. We have made changes as suggested.

- "Since previous studies reported that maternal smoking significantly affects DNA methylation. To consider this we included the variable "smoking" despite its smaller effect on data variance." - This is precisely what I was referring to above at confounder adjustment selection. This is a more appropriate way of selecting potential confounders for adjustment.

Thank you. In the revision, we now make explicit that covariate selection is a priori and driven by domain knowledge rather than statistical tests (SOV). SOV is only used as a confirmation of selected variables.

Specifically, we select potential confounders based on prior literature and biological rationale: maternal smoking, maternal age, ethnicity/ancestry, BMI, and parity. Smoking is retained regardless of its small share of variance because it is a well-established determinant of DNA methylation.

Also, we adjust for estimated cell-type proportions to address cell-mixture bias in bulk cord-blood EWAS (standard practice), while using the SOV analysis only as a descriptive confirmation that these variables capture major sources of heterogeneity—not as a criterion for inclusion/exclusion.

- "In conclusion, we found that the observed DNA methylation variation among the whole cord blood samples is primarily associated with cell type differences rather than severe PE." - According to your analysis, this is an incorrect interpretation. You have no proof of that, as you adjusted the model for a range of other variables as well. E.g., it is entirely plausible (and

probable) that gestational age alone could have resulted in the same results. In fact, this would be the way to assess this. To run parallel models and see whether the GA adjustment impacts the results, first. If differentially methylated sites remain, then you could assess whether cell proportions impact these associations.
Thank you for the comment. We removed this sentence, given the updated results.

- Related: The analysis you undertook using the Herzog et al. data, which does not have GA information, shows that adjustment for cell proportions alone makes all statistically significant sites disappear. So this is an interesting finding, but unfortunately still doesn't exclude the possibility of GA being the driver of these results.

Indeed, since gestational age (GA) information was not available in the Herzog et al. dataset, we could not directly evaluate or adjust for GA in that cohort. Therefore, while the disappearance of significant CpGs after cell proportion adjustment suggests that cell heterogeneity is a strong driver of the observed associations, we agree that this does not exclude the possibility that GA may also underlie these results.

- Instead of adjusted P values only, it would also be important to make a note of the magnitude of association differences as well.
Thank you, we have added the magnitude of changes to the association between cell proportions and GA.

- In the Results, you use the term "insignificant" as an opposite to statistically significant. This is incorrect and should not be used. Please use "not statistically significant".
Thank you, we have revised as suggested.

- After reading your Results and the parts related to the meta-analysis, I would like to emphasize again that I think excluding Kashima et al from the meta-analysis is not appropriate, and will bias your findings (just because they have null results before cell proportion adjustments, this does not mean that they should not be added to the meta-analysis, this is selective selection based on results, which is big NO). Furthermore, it is also a statistical possibility that by adjusting for cell type proportions, some differentially methylated sites BECOME statistically significant, further illustrating the flawed logic here.

We appreciate your thoughtful comment and agree with your comments. As mentioned above, we still decided to exclude Kashima et al.'s dataset in the meta-analysis because it was generated from cord blood mononuclear cells (CBMCs), whereas our in-house cohort, Ching et al., and Herzog et al. were all based on whole cord blood. Since CBMCs lack granulocytes and have a substantially different overall cell composition from whole blood, they are not appropriate for the whole-blood based meta-analysis

As suggested, we performed a differential methylation analysis on the Kashima et al. dataset, adjusting for estimated cell proportions, gestational age, maternal age, BMI, parity, fetal sex, and batch effects. Consistent with our previous observation, this analysis still did not yield any statistically significant CpGs. We have revised the manuscript to clarify this rationale.

- "The proportions of monocytes also significantly increase as gestation progresses, after adjusting for other variables (p=0.019)." - What are the adjustments here, in these analyses?
We apologize for the error. This p-value was obtained by directly regressing the cell proportion of monocytes on gestational age. We didn't do any adjustments. We have changed the sentence to:
"The proportions of monocytes also significantly increase as gestation progresses (p=0.019)"

- "These trends of cell proportions are mostly the same in the case and control groups except for monocytes, which show a potential interaction effect between PE and gestational age." - THIS is perhaps your most important finding, sort of hidden in Results; you should emphasize this more!! Please include the magnitude of interaction and the P value. This result and methodology allow you to disentangle GA, cell proportions, and PE status.

Thank you for highlighting this important point. We agree that the potential interaction between preeclampsia (PE) and gestational age (GA) on monocyte proportions is a key finding that deserves more emphasis. In our regression model including GA, PE, and their interaction term, the interaction effect for monocytes was $\beta = 0.006$ with p-value = 0.009.
Additionally, in the new revision, PE and GA also has significant interaction in granocyte and nRBCs(Figure 4b). We have revised the Results to report these values explicitly and emphasized in the Discussion that this interaction may indicate differential regulation of monocyte proportions in PE across gestation.

- "None of the interaction terms between datasets and GA turn out to be significant, as the p-values in Fig. 5." - Please check grammar in this sentence.
Thank you, we have revised the sentence to be "None of the gestational-age interaction terms were statistically significant (Fig. 5A). A non-significant interaction p-value indicates that the GA–cell-proportion trends do not differ statistically between case and control group."

Discussion

- "In this study, we showed there is a lack of association between severe 401 PE and DNA methylation level changes, in the cord blood samples of the offspring of these PE patients from multiple cohorts." - This is the first sentence of the Discussion section, but this statement is not exactly true, as this finding is only true when you explicitly mention the adjustments, which is THE key learning here.

Thank you, we revised this sentence to: "In this study, we showed there is a lack of association between severe PE and offspring's cord blood DNA methylation changes after adjusting to cord blood cell types and clinicial covariates, from multiple cohorts."

- "Instead, severe PE manifests itself impact on the offspring of the affected mother by altering the proportions of some immune cell types in the blood." - This is a misleading and flawed conclusion. First, you cannot conclude a causal relationship. Second, this association is very, very likely to be almost completely explained by gestational age. There is only very limited

evidence that supports a statistical association between cell types and PE. One is that CD8T remains borderline statistically significantly associated with severe PE vs. control status after adjustment for GA, and the second is the observed statistically significant interaction for monocytes between PE and gestational age. I would not consider these two results very robust; however, they are the key novel results that make your article stand out. I suggest some rewording to appropriately show these interesting findings, but in a way that rather emphasizes these as emerging novel hypotheses
to study in further studies (and tone down overstated conclusions in THIS study).

We thank the reviewer for this important comment and agree that our original wording overstated the conclusions. We have revised the manuscript to remove any causal language and to more accurately reflect the evidence. Specifically, we now emphasize that, after adjustment for gestational age and other covariates, the only signals that remain are (i) a borderline statistical association of CD8T cell proportions with severe PE, and (ii) a statistically significant interaction between PE and gestational age for monocyte proportions.

We have changed the sentences to “Our results suggest that severe preeclampsia may be linked to subtle shifts in offspring immune cell proportions.”

- "This observation is now expanded to their offspring, demonstrating the impact of PE." - This is a causal interpretation, which you should avoid.

Thank you—we agree and have removed causal phrasing. We have rephrased this line.
This observation is now expanded to their offspring, indicating an association between maternal PE and neonatal DNA methylation.
We also replaced similar terms (e.g., “impact,” “effect”) with “association/differences” throughout and added a note in the Discussion that causal inference is not supported by our design.

- I completely agree with your conclusion related to the statistical considerations about proper adjustment for cell proportion in these kinds of analysis. This is backed by your findings.
Thank you for the supportive comments.

- Related to Kazmi et al.: " However, they did not adjust for GA due to the concern of its confounding effect on PE." - This sounds like a very strange reasoning, as "concern of confounding" is exactly the reason for an adjustment.

Apologies for the confusion. We have revised this sentence accordingly “However, they did not adjust for gestational age, which may also mediate DNA methylation changes in addition to preeclampsia, thus need to be adjusted”.

- "We showed that GA affects the methylation pattern both directly and through cell proportion change, …" - I do not think you can conclude these causal interpretations.

Following the comment, we revised this sentence to "Additionally, we show that gestational age is associated with cell proportion change, as reported before".

- "Instead, severe PE is associated with significant changes in several cell proportions in the cord blood." - This conclusion overstates the results.
Thank you for flagging this. We removed this sentence given the new updated results.

- I lacked a more structured highlight of the CD8T association (independent from GA), and the monocyte interaction effect. These two key pieces of evidence should be emphasized a bit more, as the most important indications of potential cell proportion-related findings in terms of PE (the rest of the results reflect cell proportion changes according to GA, in my reading).

Thank you for the insightful observations. We have revised the preprocessing steps based on reviewer comments, and in the new result, CD8T is no longer significantly associated with PE after adjusting for clinical variables (**Figure 2C**).

Additionally, PE shows significant interactions with gestational age in more cells (granulocyte, monocyte and nRBC; **Figure 4B**). These intriguing findings may point to immune cell involvement in preeclampsia. However, as these results are preliminary and derived from computational deconvolution, we prefer to present them as hypotheses for future validation rather than central conclusions of this study.

To clarify this, we have added brief highlighting sentences in the Results and Discussion sections to note these associations as potential biological signals warranting further experimental investigation, while keeping the main focus of the manuscript on the lack of methylation associations after rigorous confounder adjustment.

- Further limitations of your study are that there is no information on key lifestyle variables, such as diet and physical activity, which both can have an impact on methylation patterns.
Thank you for the important suggestion. We have added this to our discussion section. "Lifestyle factors such as diet and physical activity may also influence DNA methylation patterns and should be included in future works."

Items

For all items (and supplemental items), please always include abbreviations in the item legends. These are missing from a lot of items (cell types, EWAS, RE, etc.). For all figures, please edit the axis names, grouping names, and category names from the raw output from R to human-understandable names. E.g., the Figure 2D y-axis name is "Cell_Type_Proportion_Residuals", which sounds like an R variable name; you can make these nicer. For each figure, please replace "ns" with the exact P values (e.g., an adjusted P value of 0.051 would be ns, but informative).
Thank you for the suggestions! Here are the changes we made accordingly:

1. Added missed abbreviations in item legends, including EWAS, PBMC, cell type to captions.
2. Fixed the texts on figures that don't read naturally. (Figure 2）
3. Replaced ns with exact p-values in Figure 2B, 2D, 3J, 4 and 5
4. We have changed the GA(PE) in Figure 5A to GA(Disease) to be consistent with previous figures.

Table 1: This table shows the sample characteristics. Due to the low sample size, I would suggest that the authors use nonparametric tests for the comparisons.
We have replaced t-tests with Wilcoxon test (nonparametric) to compare numeric variables.

Figure 1: This is a neat figure that shows the analytical pipeline.
Thank you.

Figure 2: The entire figure ended up very small in the downloaded PDF. Panel A) is very difficult to interpret, and not really related to the results in the paper; you never mention hierarchical clustering in the paper, so I would remove this plot. Panel B) is informative; however, it does not explain how different numbers of stars reflect different levels of statistical significance, in the figure legend. Why not include exact P values? Panel C) is informative, but would remove the coloring as colors here do not include extra information. Panel D) is informative - are these confounder-adjusted means?

Thank you for the helpful suggestions regarding Figure 2. We agree that Panel A is not central to our results and have removed it. For Panel B, we used exact P values instead of stars as suggested. For Panel C, we removed unnecessary coloring. Finally, for Panel D, we clarified in the figure legend that the boxplots display cell-type proportions after adjustment for confounders, and we explicitly indicate the modeling approach used. We also removed dashed lines from figure texts and increased the resolution.

Figure 3. Panels A-D-G) Please remove the color. In the other panels, in the volcano plots, please remove the written-out CpG names; these are irrelevant for this study. Panel J) Are these confounder-adjusted means?

We have removed the colors from SOV plots (Figure 3A, 3D, 3G). The cell proportion and test results shown in Figure J is not adjusted for confounders, because Herzog et al. doesn't provide any clinical/demographic information for their samples, so we are unable to adjust for confounders on the pooled dataset.

Figure 4. These plots are great, but I would be more interested in the interaction magnitudes (GA x PE) and interaction P values than the main associations between GA and cell proportions. Again, these are your key results in my opinion.

Thank you, we have added the magnitudes and p-values for the interaction term between GA and PE.

Figure 5. I don't really understand the utility of these analyses. Why is it so relevant whether there is an interaction between PBMC and WB? The previous interaction plot (Figure 4B) was great, but I do not get the utility of this one.

We apologize for any confusion. The objective of Figure 5 is to demonstrate that the association between cell proportions and gestational age observed in our in-house whole cord blood data can be replicated in an external dataset from cord blood mononuclear cells (Kashima et al. 2021) To illustrate consistency, we plotted our whole blood results alongside Kashima's CBMC results, stratified by sample group (preeclampsia vs. control). The interaction term tests whether the relationship between gestational age and cell proportions differs by dataset. A non-significant interaction indicates that the gestational age trends are consistent across datasets. The PBMC label in the figure should be CBMC instead. We have fixed the typo.

Supplemental Items

Supplemental Figure 1. This is a very nice flowchart showing the epigenetic processing pipeline. This is a very technical description of the flow, which goes beyond my expertise, so I am unable to ascertain whether this is fully appropriate or not.
Thank you for the positive feedback on Supplemental Figure 1. This flowchart was designed to clearly illustrate the DNA methylation processing and analysis pipeline, following the widely used *ChAMP* pipeline and other established tools (e.g., ComBat for batch correction, limma for EWAS). These are standard and well-validated methods in epigenetic studies.

Supplemental Figure 2. This is related to the data quality control. This is a very technical description of the quality control process, which goes beyond my expertise, so I am unable to ascertain whether this is fully appropriate or not.

We appreciate the reviewer's feedback on Supplemental Figure 2.These are standard steps implemented in the *ChAMP* R package and recommended in prior literature, and we included the figure to transparently document each step and its impact on the data.

Supplemental Figure 3. Impact of smoking adjustment visualized using volcano plots, showing little to no impact. The only comment I have here is to include in the legend that the red color indicates statistical significance (and looking at this plot, the reader can see that there are no statistically significant CpGs in either panel).

Thank you for the feedback. Since multiple reviewers suggested not to use the SOV method to select confounders, we have revised our analysis to include all clinical confounders (including smoking) directly in the *limma* regression, as shown in the updated main Figure 3. To avoid redundancy, we have therefore removed Supplemental Figure 3 from the revised manuscript.

Supplemental Figure 4. Volcano plot showing differentially methylated sites in the Kashima data. All clear.

Thank you.

Supplemental Figure 5. I have the same comments for this figure as I already had for the main figure items.

Thank you for your comments. We have revised supplementary figure 5 following the same instruction you provided for the main figures.

Supplemental Table 1. For each variable in the table (column 1), please include units and/or reference values (e.g., for ethnicity, what is the reference category). Please include units for the cell proportions as well. Are these %?\
We have added the units for maternal age (years) and gestational age (weeks), as well as the reference category for ethnicity, as suggested. Cell proportions are presented as fractions of the total, without units, and by definition sum to 1.

Supplemental Table 2. These are the top 20 differentially methylated CpGs, but I am not sure what comparison this relates to, and what the adjustments are. The table has no legend, so it is unclear what statistical tests were used and what each column means. This is likely a raw output from a statistical software, which needs to be edited further for "human consumption". Please clean up this table so that all parts are understandable.

Thank you for the feedback. We are sorry for the confusion. Supplementary Table 2 includes the results of top 20 differentially methylated regions (DMRs). The result was from comparing severe preeclampsia and controls. They are adjusted to the same confounders as the limma regression model (cell proportion, gestational age, maternal age, parity, BMI, ethnicity and smoking). We added a description of each column in the table caption to improve readability.

"I previously reviewed this paper previously in Research Hub and you can read these comments via the Research Hub review page here:
https://nam02.safelinks.protection.outlook.com/?url=https%3A%2F%2Fwww.researchhub.com%2Fpaper%2F9416337%2Fcell-type-proportions-rather-than-dna-methylation-in-the-cord-blood-show-significant-associations-with-severe-preeclampsia%2Freviews&data=05%7C02%7Clgarmire%40med.umich.edu%7C612fd2770dc44658fa8208ddc463354f%7C1f41d613d3a14ead918d2a25b10de330%7C0%7C0%7C638882652585374779%7CUnknown%7CTWFpbGZsb3d8eyJFbXB0eU1hcGkiOnRydWUsIlYiOiIwLjAuMDAwMCIsIlAiOiJXaW4zMiIsIkFOIjoiTWFpbCIsIldUIjoyfQ%3D%3D%7C0%7C%7C%7C&sdata=CMBybQ%2B%2BXX17tXBMHuVPMBTff65eCRCYC6bOoTvJmDM%3D&reserved=0

The original preprint linked to the Research Hub review is here:
https://nam02.safelinks.protection.outlook.com/?url=https%3A%2F%2Fwww.medrxiv.org%2Fcontent%2F10.1101%2F2025.06.09.25329270v1&data=05%7C02%7Clgarmire%40med.umich.edu%7C612fd2770dc44658fa8208ddc463354f%7C1f41d613d3a14ead918d2a25b10de330%7C0%7C0%7C638882652585391149%7CUnknown%7CTWFpbGZsb3d8eyJFbXB0eU1hcGkiOnRy

dWUsIlYiOiIwLjAuMDAwMCIsIlAiOiJXaW4zMiIsIkFOIjoiTWFpbCIsIldUIjoyfQ%3D%3D%7C0%7C%7C%7C&sdata=9ht5uhlaT4rGTcEDek%2FQJQgoxiUQKLvaNxDC%2F18GR9s%3D&reserved=0"

Reviewer #2: The authors investigate DNA methylation differences between preeclampsia cases and controls. The main finding of the study is that previously reported differences were likely caused by cell type heterogeneity and gestational age mismatch. Given what is known about the issues of cellular heterogeneity the finding is not surprising and can be considered to fall into the category of a "negative result". However, in my opinion findings like this provide a step forward in understanding the real relationships between epigenetics, cell type differences, and disease. As such this result should be relevant to anyone interested in DNA modification dynamics in preeclampsia and possibly the investigation of epigenetic differences in birth and pregnancy studies more generally.
Thank you for your comments, we appreciate that the reviewer concurs with us on the importance of reporting replicable "negative results".

I have some comments and proposed additions to the methods and results sections. However, in most cases these will be suggestions for improvement rather than hard flaws with the study.
Thank you!

Introduction:

The introduction seems clear and succinct. It introduces the problem, shows what has been done before, and highlights major finding disagreements between the results of the studies conducted so far, which clearly indicates that there is a problem. Then the authors propose a potential solution to this problem - performing EWAS while adjusting for cell type proportions and other necessary covariates, such as gestational age.

The only suggestion I have for introduction is to consider mentioning the (exact or approximate) sample sizes of each previous study. I was able to find this information in the next section - "materials and methods", and after seeing how small the sample sizes were it was less surprising that their results disagreed. Even the previous reported meta analysis (Kazmi et. al.) only had 135 cases, which would not be considered "large scale". This should be turned into another item highlighting the need of the present study - to integrate together all the previous studies conducted so far in order to boost sample size further.

Thank you for the suggestion! We have added the number fo preeclampsia cases to each mentioned study in the introduction section.

Methods:

The methods section is written very clearly, it depicts what has been done both in terms of data preparation and subsequent data analysis. In addition there is a link to a GitHub repository containing the code. The data-preparation file contained in the GitHub repository follows the steps mentioned in the methods - even the removal of one outlier and imputation of missing BMI values. My only suggestion for the git repository would be to include a README file with some guidelines describing how to run the scripts on some actual datasets.

Thank you, we have added a README file to the github repository as suggested. The file includes the version of R and each package, the orders of each script as they appeared, and a quick guide to replicate our results.

The section describing cohorts, sample preparation, and DNA methylation profiling are all excellent. It lists the sample sizes as well as GEO repository IDs for each dataset that is used in the study. The methods for DNA extraction seem standard and DNA methylation profiling with arrays was performed by a dedicated facility. The only thing potentially missing here is to mention how the step of bisulphite conversion was done.
We used EZ DNA Methylation kit for the bisulfite conversion step (Zymo Research) as recommended by the Infinium Methylation BeadChips manual. We have added this information to the method section.

The data preparation steps can be improved.

First, it is not fully clear how the probes were filtered by detection p-values. The manuscript says that probes with detection p-value below 0.01 were removed. But the list of such probes will be different for each sample. Was the probe removed even if a single sample had a poor detection p-value? Or was the strategy the same as for low bead count (5% of samples had to have a low detection p-value)? In addition consider an alternative and more robust method for obtaining detection p-values (PMID 30678737)
Thank you for the comment. We followed the *ChAMP* pipeline for filtering probes and samples based on detection p-values. Specifically, we first computed the detection p-value matrix (probe × sample) and identified measurements with $p > 0.01$ as failed. We then calculated the proportion of failed probes per sample and removed samples with more than 10% failed probes. After that, probes that failed in any sample were also removed.

We have clarified these details in the revised Methods section under “DNA methylation data pre-processing and quality control”

We also examined the recommended non-specific fluorescence (NSP) method proposed by Heiss & Just (PMID 30678737), which uses an alternative background model and applies a highly stringent cutoff, equivalent to set pvalue < 1e-40 in traditional negative control probes (used in ChAMP) . While conceptually attractive, this method identifies a substantially larger number of undetected probes—approximately 69,517 in our dataset compared with 7,941 under the ChAMP criterion. Also, this approach is not compatible with limma, which automatically excludes probes containing any missing values—functionally equivalent to removing all probes

with any failed detection. For this reason, we retained the standard *ChAMP/minfi* filtering strategy, which is widely used and more compatible with our analysis framework.

It is unclear what "removing probes with no match CpG site" mean. Are these non-CG (CH) probes as well as control (rs) probes? If yes, in my opinion, that would be a clearer wording.

Thanks for pointing this out. Yes, by “removing probes with no matched CpG site” we referred to non-cg labelled probes(ch probes). The non-CpG (ch) probes on the Illumina MethylationEPIC array are carried over from the older 450K array and target methylation at cytosines not followed by a guanine. This step to remove these probes is in the standard processing pipeline of the “ChAMP” package. We did not remove the rs probes (SNPs) from our analysis.

We agree that our original wording was unclear and have revised the Methods to specify that we removed ch probes.

Removing cross-hybridizing probes (that map to multiple locations in the genome) is clear. But based on the reported number I think the annotation for which probes have cross-hybridization issues is probably outdated. Here I would recommend to use a modern and comprehensive annotation of probe issues compiled by Zhou.et.al (PMID 27924034, https://nam02.safelinks.protection.outlook.com/?url=https%3A%2F%2Fzwdzwd.github.io%2FInfiniumAnnotation&data=05%7C02%7Clgarmire%40med.umich.edu%7C612fd2770dc44658fa8208ddc463354f%7C1f41d613d3a14ead918d2a25b10de330%7C0%7C0%7C638882652585407400%7CUnknown%7CTWFpbGZsb3d8eyJFbXB0eU1hcGkiOnRydWUsIlYiOiIwLjAuMDAwMCIsIlAiOiJXaW4zMiIsIkFOIjoiTWFpbCIsIldUIjoyfQ%3D%3D%7C0%7C%7C%7C&sdata=%2BsS112HQBhChMSHuKr84NagY%2Fc5M61s9IRzQcPnd3hE%3D&reserved=0).

Thank you for the suggestion. We have extracted the suggested new list of cross-hybridization probes and updated our preprocessing pipeline accordingly. The old reference used by ChAMP is Nordlund’s Genome Biology Paper in 2013, which includes around 8,000 cross-hybridization probes. The provided reference by Zhou et al. includes 25,194 probes. The final filtered dataset has 803,359 probes in total (see updated supplementary figure 1). This change doesn’t affect our conclusions.

The normalization was done with BMIQ, which adjusts the intensities between Type I and Type II probes. I think this is a good strategy but probably will not be that relevant for the results of the paper, as the analysis will not involve comparison between probes, but between samples within the same probe.

Thanks for the comment. While it is true that our downstream analyses primarily compare samples within the same probe, normalization remains a critical preprocessing step for Illumina methylation arrays. Without normalization, systematic differences between Type I and Type II probes can distort β-value distributions, introduce technical bias, and reduce comparability

across samples. The BMIQ method specifically corrects for probe-type bias while preserving biological variation, which improves data quality and ensures that spurious probe-type effects do not confound downstream analyses[1, 2]. For this reason, normalization is considered a standard and necessary step in DNA methylation data processing pipelines[3], and we believe it cannot be omitted even if our primary comparisons are sample-level rather than probe-type level.

[1] Andrew E. Teschendorff, Francesco Marabita, Matthias Lechner, Thomas Bartlett, Jesper Tegner, David Gomez-Cabrero, Stephan Beck, A beta-mixture quantile normalization method for correcting probe design bias in Illumina Infinium 450 k DNA methylation data, Bioinformatics, Volume 29, Issue 2, January 2013, Pages 189–196, https://doi-org.proxy.lib.umich.edu/10.1093/bioinformatics/bts680
[2] Wang, Z., Wu, X. & Wang, Y. A framework for analyzing DNA methylation data from Illumina Infinium HumanMethylation450 BeadChip. BMC Bioinformatics 19 (Suppl 5), 115 (2018). https://doi.org/10.1186/s12859-018-2096-3
[3] Welsh, H., Batalha, C.M.P.F., Li, W. et al. A systematic evaluation of normalization methods and probe replicability using infinium EPIC methylation data. Clin Epigenet 15, 41 (2023). https://doi.org/10.1186/s13148-023-01459-z

ComBat was used in order to deal with potential batch effects. I would recommend against using ComBat as it has been repeatedly reported to cause complex issues and should not be used without strict supervision (PMIDs 29616078 and 32605541, among others). Since batch effects are expected to manifest as mean-intensity differences between experiments and sentrix plates they can be controlled in the same way as cell type proportion effects - by including them in the model. Furthermore ComBat might interfere with cell type deconvolution, since the reference datasets used for deconvolution were likely not treated with ComBat.

Thank you for the insightful suggestion. We have updated the data processing pipeline by removing the ComBat batch correction step and instead including the batch variables (slide and array) as dummy variables in the regression model, as recommended. Corresponding revisions have been made in the Methods section, and the Results have been updated accordingly.

Cell type deconvolution step is a bit unclear. The manuscript states that it used a reference by Gervin et. al. and in the software section it states that the R package EpiDISH was used to perform the deconvolution. However, as fas as I am aware, EpiDISH does not contain a reference from Gervin et. al. Here a clearer specification about how deconvolution was performed should be provided.

We only used the cell type deconvolution functions from EpiDISH package. By the time we wrote this manuscript, there were only 3 adult blood references in the package, no cord blood reference. We get the cord blood cell type reference from Gervin et al.,the current state-of-the-art cord blood reference, which combined 4 previous published cord blood references.

We have explained the deconvolution pipeline in the method section under “cell type deconvolution” and the “software usage and code availability” subsection.

In addition, the newer version of EpiDISH has a "uni life" reference (centUniLife.m object) which contains 19 cell types (cord blood + 12 extended cell types, such as memory/naive stratification for T lymphocytes, DOI https://nam02.safelinks.protection.outlook.com/?url=https%3A%2F%2Fdoi.org%2F10.1186%2Fs13073-025-01489-7&data=05%7C02%7Clgarmire%40med.umich.edu%7C612fd2770dc44658fa8208ddc463354f%7C1f41d613d3a14ead918d2a25b10de330%7C0%7C0%7C638882652585423205%7CUnknown%7CTWFpbGZsb3d8eyJFbXB0eU1hcGkiOnRydWUsIlYiOiIwLjAuMDAwMCIsIlAiOiJXaW4zMiIsIkFOIjoiTWFpbCIsIldUIjoyfQ%3D%3D%7C0%7C%7C%7C&sdata=ioJoHEuXj0MJ0xhK3Kr8nECffm8fxmMBsvG5RKds11A%3D&reserved=0). It would be interesting to test if this reference would allow a more granular specification of cell type differences between PE and controls.

Thank you for the suggestion. The study by Guo et al. developed the “UniLife” reference by combining 7 cord blood cell types with 12 adult blood cell types, aiming to create a unified reference applicable across all age groups, especially toddlers. Notably, the 7 cord blood cell types in UniLife were derived from Gervin et al., the same source we used in our manuscript. Even though Guo et al.’s reference includes more total cell types, the additional adult cell types are less relevant for cord blood–only analyses.

We applied the UniLife reference to our own cord blood data. As expected, most adult cell types were not detected. **However, cCD8Tnv, aEos, and aNeu which are supposedly adult-only cell types, show up with significant proportions in our cord blood samples** which have no adult cell types. This result shows that UniLife is not optimal for cord blood studies, so we keep Gervin et al.–based reference.

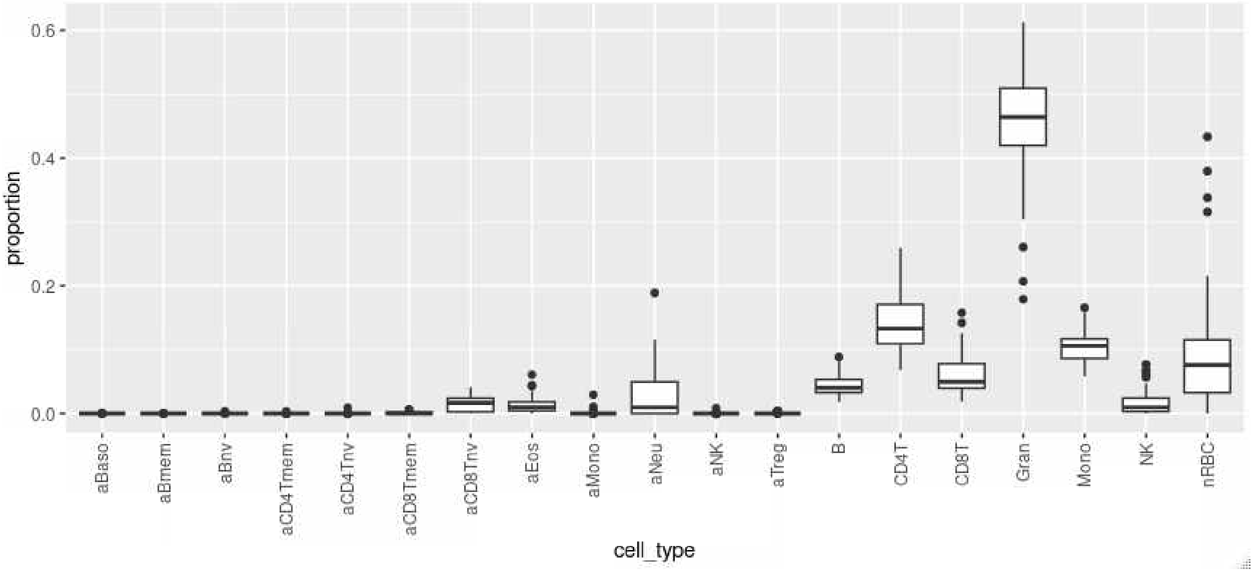

The strategy for selecting clinical confoudners raises a small concern. The effects were averaged across all investigated CpGs. However, if a confounder (such as BMI) has an influence on a small set of cytosines it will not be detected by such a global analysis and yet it would still confound those specific CpGs in site-by-site comparisons.

Thank you, this is a very important suggestion. We have changed the analysis so that all confounders (clinical variables, cell proportions) are adjusted in limma regression. SOV analysis is only used as a confirmation of confounder impact, not as a way to select confounders.

The volcano plots in figure 3 have been updated to reflect this change. The change doesn't affect previous results.

For EWAS the limma package is used in order to perform a modulated analysis which pools the variance estimates across many CpGs in order to increase statistical power. The corretion for multiple testing is also performed. I can see no issues with the EWAS step.
Thank you for your supportive comment.

The meta analysis and combination of cohorts again uses ComBat in order to remove batch effect. As mentioned previously even a single application of ComBat can cause issues, and if it is used again on top of datasets already pre-processed using ComBat it can only make things worse. Instead, I would simply inlcude a dummy variable for cohort in a regression model supplied to limma.

Thank you for raising this important point. We have carefully addressed this concern by excluding ComBat in each individual cohort analysis, ensuring that no dataset underwent ComBat correction more than once. In the pooled dataset, we applied ComBat solely to harmonize the data across cohorts rather than to remove within-cohort batch effects. We chose ComBat because the differences among cohorts are likely non-linear and may not be fully captured by including cohort as a dummy variable in the regression model.

Including additional preterm samples as controls seems like a good idea. But it is uncler why there is an additional correction for null-distribution bias and inflation ("bacon") here, which was not used in any of the previous analyses.

Thank you for the comment. We applied *bacon* only to the analysis that included idiopathic preterm samples because it showed noticeable inflation in the test statistics ($\lambda = 1.383$), indicating potential residual bias. The *bacon* method (van Iterson et al., *Genome Biol*, 2017)

estimates and corrects both bias and inflation in EWAS summary statistics. The community widely agrees that an inflation score > 1.2 is problematic and needs to be adjusted.

All other analyses have inflation score within normal range: for in-house data, the final model has □ = 0.922; for Ching et al. data, the full model has □ = 1.125; for the Herzog et al. data, the model has □ = 0.957; for the pooled dataset, the full model has □ = 0.971. All these are acceptable.

However, for the analysis with idiopathic preterm samples, the □ = 1.383. It's recommended that a bacon adjustment be made to reduce the bias. Below is a figure showing the observed p-values vs the expected p-values before and after Bacon correction on this dataset. Therefore, we only applied the Bacon adjustment to this dataset.

For the detection of differentially methylated regions the authors used "bumphunter" which is a well known algorithm. The selected method has a lot of parameters, but none of them are listed here. It would be good to expand the description a bit and mention what parameters were used in order to detect DMR's. And if default settings were used, that could be specified as well.

Thank you for the suggestion! We have added the detailed choice of parameters for "bumphunter" function in the method section: "We used the "clustermaker" function to identify clusters, with default parameters; the "bootstrap" method to generate null candidate regions; 0.2 and -0.2 as the upper and lower bounds of the candidate regions (more details of parameter choice see "5.3 - DMR.R" in the code). "

Results:

The section about cell proportion variability across clinical variables is clear and well examined. One comment here is that it would be really interesting to check if UniLife reference might give additional insights.

We apologize for not being able to incorporate this new information. We tried to use UniLife to deconvolute our data, but couldn't detect any additional cell type because the extended cell types in UniLife are from adult blood.

We have added the need of more fine grid cord blood reference in the discussion section. "Now, cord blood references only contain 6-7 cell types; a reference with finer grid cell types can yield more insights."

It also seems relevant to check whether the same patterns of cell type proportion difference could be reproduced in other cohorts. Seems like this comparison was performed and described later (in the section talking about GA and cell type association). But I would consider merging it with this section for better structure and flow.

Thank you for this helpful suggestion. We did examine the cell type proportion difference in the pooled cohort and presented it in Figure 3J, which shows a trend consistent with our in-house results in Figure 2B.

However, we were unable to generate fully confounder-adjusted boxplots for the public datasets, as they al have limitations: Ching et al.'s cohort includes only early-onset PE, leading to a gestational age distribution that is not comparable with our data. Herzog et al. did not provide gestational age information. Kashima et al. did include gestational age, but the samples were PBMCs (thus lacking granulocytes). For this dataset, we compared PE and controls without adjustment for other confounders, and the results are shown alongside our in-house cohort in Figure 5.

I would also recommend one additional step to make sure that cell type heterogeneity is the sole cause of PE DNA methylation differences. Currently a case can be made that a model with all the additional adjustments has 10+ more covariates when compared with a model that does not adjust for cell types and additional clinical variates. Given low cohort sample sizes a more complex model will be severely under-powered. Hence, it could be suspected that the lack of significant differences (after adjustment for multiple testing) can also be a side effect of low statistical power. In order to test this posibility I would recommend following a similar strategy that was done in a meta analysis of schizophrenia (PMID 33646943). Briefly, I suggest investigating the cytosines reported as significantly associated with PE (when no adjustment for confounders is performed) and to compare their effect sizes (DNA methylation differences) and effect directions (hypo vs hyper methylated) before and after correction for cell type proportions. If the results were indeed caused by cell type heterogeneity this analysis will reveal that the effect sizes became a lot smaller and the directions of effects are no longer consistent with the original findings. However, if the effect direction (hypo vs hyper) after adjustments still correlates with original results then the problem of low power could be suspected as a culprit.

Thank you for the suggestion! We plotted the effect sizes of CpGs before and after confounder adjustment. We used t-statistics from limma regression as the effect size. As shown in the figure below, the magnitude of effective size shrank after adjustment, and around 37% cpgs changed directions, consistent with your prediction that significant cpgs before adjustment are indeed caused by cell type heterogeneity.

We have added this new result to result section and supplementary figure 5.

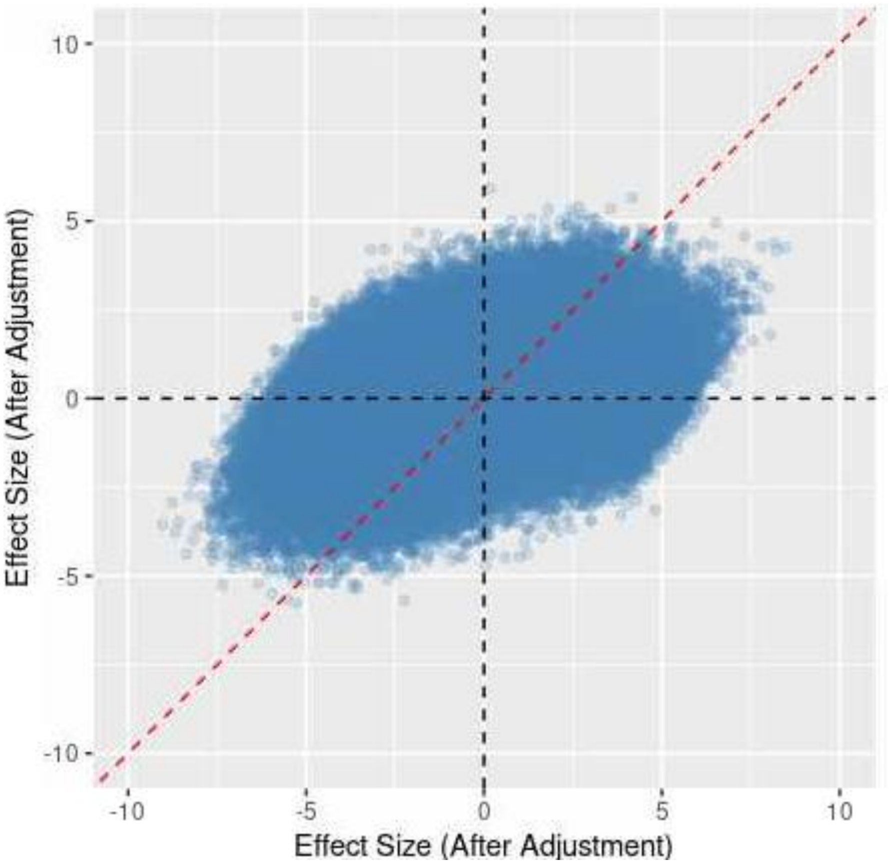

An additional analysis that would be really interesting to see is whether the original EWAS hits were not caused by a mismatch in GA. Since there is a dataset with pre-term and full-term samples it is possible to look for differentially methylated cytosines between pre- and full- without any adjustment for cell types. And then to compare whether those cytosines overlap with the cytosines reported to be different between PE and controls. This would strongly indicate that one of the causes of previous results is GA mismatch and make a strong case for taking GA into account when doing EWAS in any study related to pregnancy and birth.

Thank you for this insightful suggestion. We performed the proposed analysis by computing differentially methylated probes between preterm and full-term controls without any cell-type adjustment using the merged idiopathic preterm dataset. Out of 450,000 probes, 220,000 were significant. When we compared these to the unadjusted results from Ching et al.'s dataset (analyzed in the same manner, without confounder adjustment), 19,297 of the 35,936 significant probes overlapped with the preterm versus full-term results. We also compared the result with Herzog et al. data, 14,169 out of 2,4368 probes overlapped.

Both datasets have slightly over 50% overlaps with the preterm vs fullterm probes, which supports the interpretation that gestational age mismatch likely contributed to the previously reported EWAS findings, emphasizing the importance of GA adjustment in pregnancy-related methylation studies. However, other confounders such as patient clinical variables, batch effect may also contributes to the biases in the previous studies.

Discussion:

The discussion is very professional and clear. It highlights the issue of cellular heterogeneity and lists examples of this issue in previous EWAS studies. It spends considerable amount of time talking about potential limitations, which include small sample sizes and using computationally estimated cell type proportions.

One thing I would like to see added here is a discussion about more granular cell type decomposition and how that could play a role. For example, B lymphocytes might not show an effect between PE and controls, but the effect could still be present in the proportion of naive and mature B cells.

We apologize for not being able to incorporate this new information. The naïve and memory B-cell signatures in the UniLife reference are derived from adult blood, and when we applied UniLife to our cord blood data, the algorithm did not detect any adult naïve or memory B-cells.

We have acknowledged in the Discussion that investigating finer-grained cell proportion changes remains an important direction for future work.

Another thing I would like to see added is a point about the broader context of the findings. The issues investigated by the authors are likely relevant not only to preeclampsia cases but to all epigenetic research conducted on cohorts related to pregnancy outcomes. I would like to see a cautionary note aiming at this broader target.

Thank you, this is a very valuable suggestion. We have added a sentence in the discussion section to expand our conclusion to a broader scale. “We also aim to emphasize the importance of variable adjustment in EWAS studies of pregnancy-related diseases, as drastic changes in cell proportions during pregnancy can strongly influence DNA methylation patterns.”

Other:

Some wording and sentences

1. "We validated this lack of CpG changes using multiple published cord blood methylation datasets."

Should be more precise. These are not "CpG" changes but cytosine methylation changes.

Thank you for pointing this out. We agree that ‘CpG methylation change’ is not precise, because methyl groups are located on cytosines. At the same time, ‘cytosine methylation’ may also be misleading, since not all cytosine sites are CpGs, and only CpGs are measured by the probes. To avoid confusion, we have revised the wording from ‘CpG changes’ to ‘DNA methylation changes’.

2. "Cord blood consists of many diverse cell types, each with a distinct epigenome profile as defined."

Unclear what the last two words "as defined" are trying to say. I think they should be removed?

We have removed the redundant “as defined” from the sentence.

3. SVD stands for "Singular Value Decomposition", not "Singular Vector Decomposition".
Thank you, we have corrected this.

Some comments about the figures:

Figure 1. Study overview and experiment design.

All clear and nicely presented.

Thank you for your supportive comments.

Figure 2. Cell types in samples.

The samples in heatmap are currently clustered by granulocyte count, which is not that interesting. Consider arranging the samples by diseases status instead.
We removed figure 2A as suggested by another reviewer, because it’s less informative.

Figure labels (in boxplots) have underscores in them.
We have removed the underscores in the labels.

Figure 3.

The title of this figure is a bit long, I would move the part about cohorts and figure letters into the caption text instead.
We have moved the cohorts into the caption as suggested.

Some figure annotations contain underscores.
We have removed the underscores in annotations.

The "p-value" should not be capitalized (in volcano plots).
Thank you, we have revised to use lower-case p in “p-value”.

Figure 4. Cell type proportions in relationship with gestational age.

I would recommend showing the actual p-values instead of writing NS. Some readers might want to draw their own conclusions and be able to choose their own p-value cut-offs.

Thank you, we have added actual p-values for all cell types.

Figure 5. Cell proportions in relationship with gestational age are coherent in two different datasets

The term "P-interaction" is a bit awkward, consider using "interaction p-value" instead.
Thank you, we have changed “P-interaction” to “interaction p-value”

Supplementary Figure 1.

All clear.

Supplementary Figure 2.

Density plot B is said to be a result of density plot (A) after removing a single sample. However, for some reason, seems like more than one sample (density line) disappeared from A. Maybe more than one outlier was removed here?

We apologize for the earlier mistake. The difference between plots A and B is due to data normalization and the removal of one low-quality sample. In plot B, the DNA methylation signals were normalized using the BMIQ algorithm, which reduces technical variation and makes the lines appear more consistent.

We have revised the caption for supplementary figure 2 to include this revision.

Supplementary Figure 4.

"p-value" should not be capitalized.
Revised.

Supplementary Figure 5.

Some underscores in figure labels, and CamelCase ("CellType") for boxplot.
Revised.

Table 1.

All clear.

Supplementary Table 1.

All clear.

Supplementary Table 2.

All clear.

Good study, I had a good time while reading your manuscript and read it with interest. Hope this is helpful.

P.S. I previously reviewed this paper previously in Research Hub and you can read these comments via the Research Hub review page here:
https://nam02.safelinks.protection.outlook.com/?url=https%3A%2F%2Fwww.researchhub.com%2Fpaper%2F9416337%2Fcell-type-proportions-rather-than-dna-methylation-in-the-cord-blood-show-significant-associations-with-severe-preeclampsia%2Freviews&data=05%7C02%7Clgarmire%40med.umich.edu%7C612fd2770dc44658fa8208ddc463354f%7C1f41d613d3a14ead918d2a25b10de330%7C0%7C0%7C638882652585440652%7CUnknown%7CTWFpbGZsb3d8eyJFbXB0eU1hcGkiOnRydWUsIlYiOiIwLjAuMDAwMCIsIlAiOiJXaW4zMiIsIkFOIjoiTWFpbCIsIldUIjoyfQ%3D%3D%7C0%7C%7C%7C&sdata=1o%2FJPsR%2F9A8ORU16VtYXj5bV9QKQNkrhQ5IGK61z8HY%3D&reserved=0

Reviewer #3:

Summary
This study investigates the association between cord blood DNA methylation and pre-eclampsia using both original data and several public datasets. The findings suggest that differences in DNA methylation between cases and controls are largely attributable to variations in cell type proportions, which are, in part, explained by the shorter gestational age observed in pre-eclampsia cases.

Comments

1. Main comments
- The authors tested associations between estimated cell type proportions and case/control status or gestational age using linear regression. However, since cell type proportions are compositional in nature, I recommend reanalyzing these associations using statistical methods specifically designed for compositional data (e.g., log-ratio transformations or compositional regression models).

Thank you for the important advice. Indeed, including all cell types in one regression model would be a violation of the assumptions. We employed the Isometric Log-Ratio (ILR) transformation to transform the cell proportion data when doing the differential methylation analysis, using a function from the “compositions” R package. We use ILR instead of CLR (centered log-ratio transformation) or dropping one column because ILR gave more stable and statistically valid result.

We updated all the affected analyses. See method section under "CpG-level epigenome-wide association analysis (EWAS)"

- To explore DNA methylation changes within specific cell types in relation to pre-eclampsia, it would be valuable to perform an epigenome-wide association study (EWAS) that includes an interaction term between case/control status and cell type proportions, as demonstrated in this study: https://nam02.safelinks.protection.outlook.com/?url=https%3A%2F%2Fwww.nature.com%2Farticles%2Fs41467-020-18618-y&data=05%7C02%7Clgarmire%40med.umich.edu%7C612fd2770dc44658fa8208ddc463354f%7C1f41d613d3a14ead918d2a25b10de330%7C0%7C0%7C638882652585457349%7CUnknown%7CTWFpbGZsb3d8eyJFbXB0eU1hcGkiOnRydWUsIlYiOiIwLjAuMDAwMCIsIlAiOiJXaW4zMiIsIkFOIjoiTWFpbCIsIldUIjoyfQ%3D%3D%7C0%7C%7C%7C&sdata=fzrwLeVratuq05gUjfWB%2B0k2fpJQk3aBfVIBInzzuAM%3D&reserved=0.

We did attempt to apply the cellDMC algorithm, as described in the referenced study, to test for interactions between PE status and cell type proportions. However, cellDMC did not identify any statistically significant interactions, likely reflecting the overall lack of association between PE and DNA methylation in our data. We have added a sentence in the Merthod and Results section to document this attempt and its outcome.

- Could the authors estimate the proportion of the association between pre-eclampsia and cell type proportions that is mediated by gestational age versus the proportion that is directly attributable to pre-eclampsia? A formal mediation analysis could help disentangle these effects and clarify the underlying mechanisms.

Thank you for your suggestion. We have included a mediation analysis as suggested.

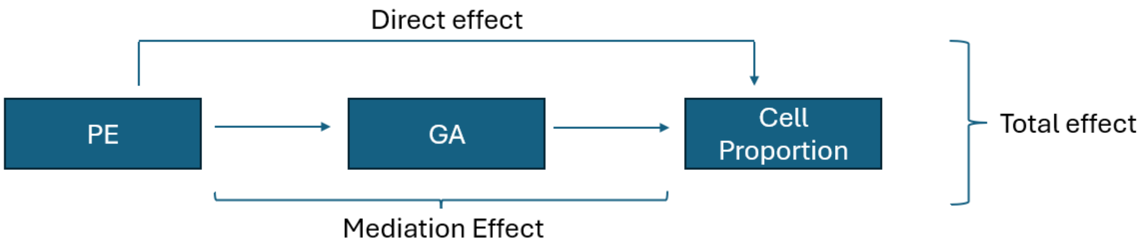

Below is the effect size of direct, indirect and total effect of this mediation analysis:

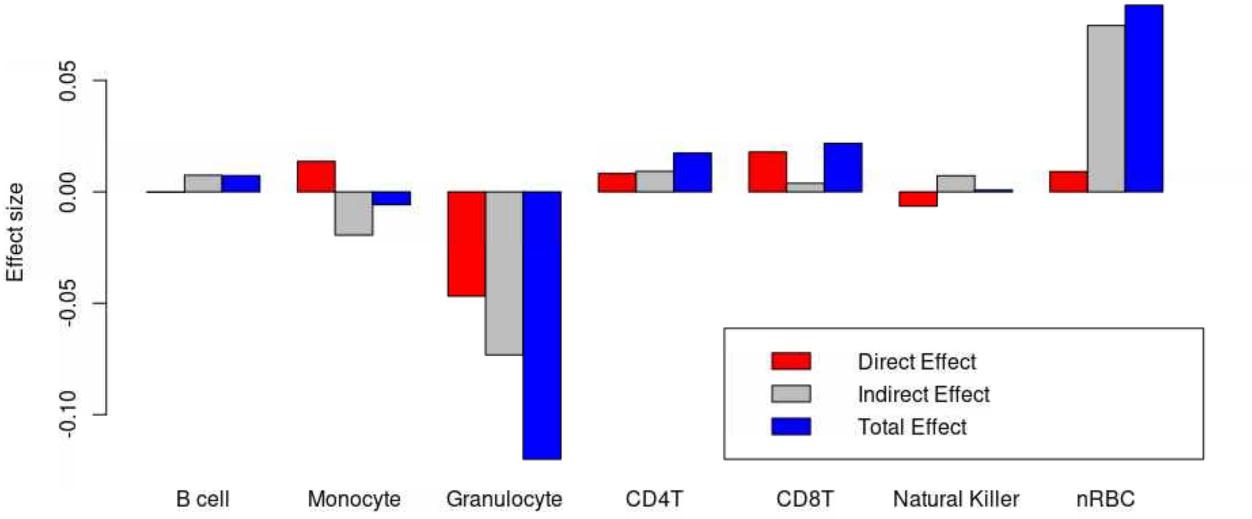

From the figure, the indirect effect(GA--cell proportion change) is the main drivers for most cell types, the only exception being CD8T, which aligns with our previous result that after adjusting to GA, only CD8T is still significantly associated with PE.

We added this analysis to the result section under "Associations between cord blood cell types and severe preeclampsia" and added these figures to Supplementary Figure 4.

2. Abstract

- "Previous epigenome-wide association studies (EWAS) on PE have produced inconsistent results, possibly due to inadequate adjustment for confounders." In which tissue? Cord blood? Placenta?

The inconsistency is observed in cord blood samples. We have added the information to the abstract.

- Authors should add sample size of the own and public data analyzed in the study.

Thank you for the suggestion. We have added the sample size for our in-house data and other mentioned public datasets in the abstract.

- "We validated this lack of CpG changes using multiple published cord blood methylation datasets." Authors should change to "CpG DNA methylation changes". Moreover, in previous sentences authors already say this "disappeared across our data, the two public datasets, and the meta-analysis.". Isn't it redundant?

Thank you. We have removed the redundant sentence "We validated this lack of CpG changes using multiple published cord blood methylation datasets".

- In the summary, authors should state clear that they talk about cord blood and not other tissues.

We added the specification of the cord blood tissue type in the summary part.

3. Methods

- The datasets analysed in the manuscript use different pre-processing pipelines. While this is fine for cases and controls processed within the study, it could introduce technical noise when using cases and controls from different studies. In particular when using the idiopathic preterm babies from one study as controls in another study. I would suggest to pre-process these controls following the same pipeline as for the cases.

Thanks for pointing this out. For the idiopathic preterm cohort, Fernando et al. mentioned in the Sentrix file that they used ChAMP pipeline for data preprocessing, the same pipeline as ours. In addition, their uploaded data doesn't include the batch information, such as slide or array or sentrix id, which is essential for preprocessing. So we have no choice but to use the processed beta matrix directly, merge it with our data and normalize the merged dataset.

- In the section "Cell-type deconvolution in umbilical cord whole blood (CB)", authors should clarify if this was done for all the datasets, including public datasets, or just for their samples.

Thank you, we have added a clarification that the same cell type deconvolution process was done to all datasets(in house and public), in the same section.

- Authors should indicate the type of meta-analysis done (fixed effects? Weighted-variance?).

We apologize for the earlier mislabeling. Our approach integrates the public datasets at the raw data level (beta matrices) so it's a pooled (mega-)analysis rather than a traditional meta-analysis. Pooled analysis is a well-recognized approach in EWAS and GWAS when raw data are available, as it enables consistent confounder adjustment across cohorts and maximizes statistical power. Given that we had access to individual-level data from each cohort, we considered pooled analysis the most appropriate method in this context. We have revised the manuscript accordingly, replacing all instances of "meta-analysis" with "pooled analysis."

We have changed all "meta-analysis" in the manuscript to "pooled analysis".

- Authors should give more details about this point "To harmonize the datasets, we applied the combat function to remove batch effects while preserving sample group information."

By “harmonize the datasets,” we mean that the raw methylation data from our in-house cohort and the public datasets were combined into a single matrix of overlapping CpGs. To reduce non-biological variation introduced by technical factors such as different array runs, slides, and laboratories, we applied the ComBat function from the sva R package. ComBat uses an empirical Bayes framework to adjust for additive and multiplicative batch effects while preserving biological variation related to the variable of interest (in this case, PE vs. control status). Specifically, we set the sample group (case/control) as a protected variable, so that ComBat corrected only for technical batch variation and not for the biological differences under study.
We have revised the Methods to include these details under the “Pooled-analysis using three datasets” subsection.

- "Lastly, we calculated differentially methylated probes again, with adjustment to the sample group and estimated cell types and plotted another volcano plot." What does "adjustment to sample group" mean?

We apologize for the confusing wording. It should be “with adjustment to estimated cell types and plotted another volcano plot.” The sample group is the predictor in the model, not a confounder to be adjusted.

We have revised the sentence.

- "We did not include data from Kashimi et al. because there was no significant cpg before the confounder adjustment." I think lack of cohort individual associations is not a reason to exclude the study from the meta-analysis and I would suggest the authors to include it.

Thank you for pointing this out. Another reason we did not include Kashima’s dataset is that, unlike the other three cohorts, it was derived from cord blood mononuclear cells (CBMCs) rather than whole cord blood. CBMC samples lack granulocytes and therefore represent a reduced set of cell types. Since cell composition is an important confounder in DNA methylation analyses, this difference would likely introduce substantial bias if the datasets were simply pooled. We have revised the manuscript to clarify this additional reason for exclusion.

- Why is the model of idiopathic preterm controls corrected using "bacon" but not the others? It sounds inconsistent. I would suggest to apply bacon or not to all the models.

Thank you for the comment. We applied *bacon* only to the analysis that included idiopathic preterm samples because it showed noticeable inflation in the test statistics ($\lambda = 1.383$), indicating potential residual bias. The *bacon* method (van Iterson et al., *Genome Biol*, 2017) estimates and corrects both bias and inflation in EWAS summary statistics. The community widely agree on an inflation score > 1.2 to be problematic and needs to be adjusted.

All other analysis have inflation score within normal range: for in-house data, the final model has □ = 0.922; for Ching et al. data, the full model has □ = 1.125; for the Herzog et al. data, the

model has □ = 0.957; for the pooled dataset, the full model has □ = 0.971. All these are acceptable.

However, for the analysis with idiopathic preterm samples, the □ = 1.383. It’s recommended that a bacon adjustment to reduce the bias. Below is a figure showing the observed p-values vs expected p-values before and after bacon correction on this dataset. Therefore, we only applied bacon adjustment on this dataset. We have the BACON inflation score in the method section.

- For the DMR I would suggest to use more than one tool and keep the overlap as final results as there are considerable differences between methods.

Thank you for the suggestion. We used bumphunter and dmrff algorithms to detect significant DMR. Unfortunately, although dmrff found one significant region on chromosome 2 (chr2 157237316 157237316), Bumphunter identified no significant region (as shown in the supplementary table 2). We also explored the options available for DMR analysis, including DMRcate, probeLasso, or comb-p. Unfortunately, all these methods require a significant CPG list as an input. Since we don’t have any significant cpg, we are unable to use these methods.

4. Results
- I would delete some of the text referring to methods from the results section. (ie. I would delete lines 263-270, and 273-275, etc…).
Thank you. We have removed some redundant text in the first paragraph of the result section.

- In Figure 1 add "gene-based EWAS" and "DMR" analyses
Fixed

5. Discussion
- "We did not detect significant differences in the inferred proportion of endothelial cells between the control and PE groups using the DNA methylation data from paired placental samples." This sentence requires a reference.

The study using paired placental samples is still ongoing. We unfortunately don’t have a preprint or paper out yet. We will remove this sentence to ensure all our statements are rigorous.

Minor comments
1. Define variables the first time they appear (ie. BMI in the abstract)
Revised.
2. Correct typos (ie. Preeclampsia(PE) - space missing)
We have ensured all parentheses have space in front of them.
3. Human gene names in cursive (ie. AVPR1a, OXTR, and .PKCB)
All gene symbols have been reformatted.
4. Change "didn't" to "did not".
Fixed.

5. Correct: "... (GSE103253). and (3)..."

We removed “and”.

6. Correct: "...cpg..."

Fixed

7. Keep naming of "combat" consistent along the text.

We have changed all “combat” to “ComBat”.

8. In the sentence "... related to Rheumatoid Arthritis after...", change disease name to non-capital letters.

Fixed

9. Change "Causcasian" for "European origin / ancestry".

Revised
