# Supplementary material for: Cord blood DNA methylation and cell-type composition are not significantly associated with severe preeclampsia after cell-type and clinical covariate adjustment
Source: Gigascience. 2026 Jan 16;15:giag002. doi: 10.1093/gigascience/giag002 (PMC13014471; doi:10.1093/gigascience/giag002)
Supplement: giag002_Authors_Response_To_Reviewer_Comments_original_submission [file giag002_authors_response_to_reviewer_comments_original_submission.pdf]

Author's Response To Reviewer Comments

Reviewer #1:

Credentials
I am an Associate Professor of Epidemiology and Health Equity at the Department of Public Health at the University of Copenl
for the past couple of years, with excellent editorial feedback. I obtained Clarivate Web of Science Academy certificates in Sci
reviewing papers related to various omics studies, including genomics, epigenomics, and lipidomics. I am familiar with epiger
necessary expertise to assess the study design and the statistical methodology of this paper. In fact, I have analyzed omics d
correct typos.

Overall

I read the study by Yang X. et al. with interest. I was particularly interested in this study due to its dual aims: I really liked th
the first aim (biological/causal) potentially misleading or overstated. I fully agree with the authors in their final message that
adjustments and not properly considering confounders. First, it appears that confounder selection was automated based on st
(e.g., via drawing up a DAG). Second, the results are often misinterpreted. The authors claim a causal relationship (perhaps
patterns, rather than cell proportions. In my opinion, the following key findings emerged here, and this would be a logical orc

1) Preeclampsia strongly associates with gestational age (known);

2) Cell proportions strongly correlate with gestational age (known);

3) CD8T cell proportions associate with preeclampsia even after gestational age adjustment (main effect, key novel finding);

4) Monocyte cell proportions show differential association with gestational age according to preeclampsia status (interaction,

Thank you for your suggestions. Our initial main motivation to submit this work, is to alert the readers the importance of adju
factors, which prompted us to share this story to the community. We figured that if we as bioinformaticians, had done the an
changes and preeclampsia first. We then report other less striking but still important findings on cell proportions in associatio

Thanks to the reminder of multiple hypothesis test, the CD8T cell proportion association with preeclampsia is not significant a

Then, I would suggest the authors dive deeper into a "causal ideation": yes, DNA methylation associations disappear after ad
methylation, altered cell proportions, and what impacts what, would be a real added value to the Discussion of this paper. Ple

Introduction

- I suggest that you do not abbreviate preeclampsia as PE. It does not change the word count, and it is easier to read the full

Thank you for the suggestion. We have replaced all "PE" with "preeclampsia" in the manuscript.

- You mention that the Kazmi et al. article reported certain findings "after adjusting for cell types". Does this mean cell propo

Yes, "adjusting for cell types" in Kazmi et al. means adjusting for estimated cell proportions. We included estimated cell propo

To avoid confusion, we changed "adjusting for cell type" to "adjusting for estimated cell proportion".

- "(Kazmi et al) However, their results did not adjust for the gestational age, the major confounder of preeclampsia" - This se

Thank you for pointing this out. Our original wording was incorrect. Gestational age is a mediator not a confounder for DNA m

In this setting, gestational age (GA) at delivery is strongly associated with the exposure (preeclampsia) and also influences th
PE → GA→ DNA methtylkation
Thus, GA is better described as a mediator rather than a confounder.

Still EWAS studies adjust for GA, in order to isolate methylation changes that are directly attributable to preeclampsia and inc

We added clarifications and explanations for this claim.

[1] Herzog EM, Eggink AJ, van der Zee M, Lagendijk J, Willemsen SP, de Jonge R, Steegers EA, Steegers-Theunissen RP. The
[2] Knihtilä HM, Kachroo P, Shadid I, Raissadati A, Peng C, McElrath TF, Litonjua AA, Demeo DL, Loscalzo J, Weiss ST, Mirzak
22. PMID: 37995466; PMCID: PMC10709000.

- The Introduction is excellent in clearly outlining a problem statement of poor confounder adjustment in epidemiological stuc
Thank you for your agreement

- Perhaps some more information in the Introduction on source tissues would be helpful to the reader. Are all the referred stu

Thanks for your insight.

In the introduction section, we first mentioned studies [6-12], conducted on placentas [7-9, 11,12] and maternal blood[6,10]

We have specified the tissue origin of mentioned studies in the introduction. We also added a sentence to explain the expecte

- "It is therefore essential to account for such heterogeneity, to improve the accuracy and sensitivity, and avoid biased conclu
Accuracy and sensitivity are terms related to predictive statistics that have not been mentioned before, so these terms feel ou

Thank you for flagging this error. Indeed, "accuracy" and "sensitivity" are metrics used to evaluate prediction problems, and

- "Particularly, to ensure that any differences in DNA methylation are due to confounding factors, the analysis needs to be ad

Thank you for pointing this out. We have fixed it in the manuscript.

- "In this study, we pay special attention to these issues to seek a plausible epigenomic association between severe PE and co
epigenetic and cellular markers IN cord blood.

We have rewritten this sentence following your suggestion: "In this study, we specifically address these issues to seek potent

- I am surprised that eclampsia is not mentioned in the Introduction as an even more severe condition. Is there any evidence

We thank the reviewer for this thoughtful comment. We now mention eclampsia as one of the most severe manifestations on

Methods

- Please write out "OBGYNs" in full words at first mention.
We wrote out the full words for OBGYN (obstetricians and gynecologists) in the method section.

- "Severe PE was characterized by OBGYNs at Kapiolani Medical Center as sustained pregnancy induced hypertension(systolic
organ dysfunction is optional. Or is it that either "elevated BP-proteinuria" or "elevated BP-organ dysfunction" combinations a

We apologize for the confusion. The definition we originally included corresponds to preeclampsia, whereas for severe preecla
proteinuria (≥5 g in a 24-hour urine specimen or ≥3+ on two random urine samples collected at least 4 hours apart), or (iii)
By organ dysfunction, we refer to the standard ACOG severe features, including thrombocytopenia (low platelet count), impa
In conclusion, "blood pressure ≥140/90 mmHg with proteinuria" and one or more of the three additional severe features are
[1] ACOG Committee on Practice Bulletins--Obstetrics. ACOG practice bulletin. Diagnosis and management of preeclampsia a

- "…and had cord blood samples remaining in the HiBR" - So your inclusion criterion is that there are samples remaining. Can
should be acknowledged, and the process's impact should be considered.

Thank you for raising this. Our inclusion criterion "samples remaining in HiBR" refers only to residual aliquot availability (i.e.,

To ensure the samples are unbiased, we matched the case and controls by age, ethnicity, and pre-pregnancy BMI. As you ca

- "We evaluated sample integrity, purity, and concentration on the Nanodrop and removed samples of low quality." - What is
We thank the reviewer for this question. The NanoDrop (Thermo Fisher Scientific) is a microvolume UV–Vis spectrophotomete
guidelines, a 260/280 ratio of ~1.8 is generally accepted as "pure" for DNA, while lower ratios may indicate protein contamin
We have added a sentence to explain the quality threshold in the method section under the "The Hawaii Biorepository (HiBR)

- It would be very useful to present a directed acyclic graph (DAG) or a causal loop diagram (CLD) at some point in the manu
investigated statistical hypotheses. For some of the variables you chose to adjust for, I cannot immediately see whether they
reader. I know that this is basic information, but this is key information for your manuscript that should be well understood b

Thank you for this helpful suggestion. We have added a directed acyclic graph (DAG) to the manuscript to formalize our stud
methylation is the outcome. Clinical variables (age, BMI, ethnicity, smoking, parity) are associated with both exposure and ou
adjusted, in order to tease out the actual direct association of preeclampsia to DNA methylation changes.
In EWAS study, researchers look for the cell-intrinsic alterations in dna methylation profile, which is independent of cell propo
independent of cell-mixture variation (Gervin et al., Clin Epigenet 2019 [22]; Zheng et al., Nat Methods 2018 [53]; Qi & Tesc
We also describe in the Methods section the three standard criteria for identifying confounders, as suggested.

- "The demographic and clinical information of the patients was collected and analyzed to identify any potential confounding e

We appreciate the request for clarification. We rephrased this to "The demographic and clinical information of the patients wa

We collected demographic and clinical variables that are commonly reported to influence DNA methylation in cord blood (inclu
each CpG site was regressed on these variables and F-statistics were averaged across sites. Variables with an average F-stat

However, as you and other reviewers pointed out, a statistical test like SOV shouldn't be used to identify confounders, becau

- "To validate the observations in the HiBR cohort, we did an exhaustive search among published work of cord blood (includin
establish a search term or involve a librarian? You refer to three papers, but it is unclear how these articles were identified.

We thank the reviewer for this comment and agree that our wording was imprecise. By "exhaustive search," we meant that w

To avoid confusion, we have revised the text: "We searched PubMed for published studies of preeclampsia and cord blood DN

- "baby gender, and baby birth weight" - While, of course, this is understandable, I am wondering if "baby" is scientifically ap
Thank you for pointing this out. We have changed all "baby" in the manuscript to "infant. We kept the 'babysex' term in figur

- "including 10 early-onsets and 13 late-onsets" - You previously introduced abbreviations for these: EOPE, LOPE. I suggest y
Thank you for flagging this! We have changed "early-onsets" and "late-onsets" to "EOPE" and "LOPE" to maintain consistency

- "For Herzog EM et al and Kashima K et al datasets, we directly used the beta matrix deposited to GEO." - Please explain wh

The "beta matrix" is the processed DNA-methylation data: each entry is a β-value between 0 and 1, representing the proport

GEO refers to the Gene Expression Omnibus, the NCBI-hosted public repository where high-throughput functional genomics d

We have added an explanation of “beta-value matrix” to the manuscript and the full name of GEO.

- "To disentangle the effect of PE and small GA" - Please refer to this as associations, as effect implies causality, which canno
"effect size".
Thank you, this is an important suggestion. We have replaced the word “effect” with association to avoid misinterpretation fo

- "idiopathic preterm birth samples" - What does idiopathic mean? Please explain for the general readership.
Certainly, "idiopathic preterm birth samples" refers to samples from cases of preterm birth where the cause is not known. Ac
method section when we introduce the Fernando dataset.

[1]Fernando F, Keijser R, Henneman P, van der Kevie-Kersemaekers AM, Mannens MM, van der Post JA, Afink GB, Ris-Stalpe

- For Fernando et al, you report: "We directly used the normalized matrix deposited to GEO." - Above, for Herzog and Kashim
Yes, the uploaded data in Fernando et al. is the normalized version of the aforementioned beta matrix. Usually, normalizatior

We have changed the phrase to “normalized beta matrix” to avoid confusion.

- At first mention, please write out the full names of CD4T and CD8T cells.
Thank you, we have changed the abbreviation of cd4t, cd8t and b cell to their full name at the first mention.

- For all potential confounders listed under "Clinical confounders and source of variance analysis", please list categories and p

We thank the reviewer for this helpful comment. In the revised manuscript, we now provide precise definitions and categories
Pacific Islander, Caucasian, and Other, as recorded in the medical record.

We have updated the Methods to clarify these definitions.

- "We performed the source of variance (SOV) analysis on these clinical variables and previously estimated sample cell propo
implying that confounders were selected purely based on statistical relationships. This is not appropriate. Confounders should
outcome. 3) The variable must not be on a causal pathway. As suggested above, I would much prefer that you visualize your

Thank you this valuable comment. We agree that a priori biological and epidemiological reasoning is more appropriate than re
smoking status, ethnicity, and gestational age at delivery). These were treated as potential confounders based on prior know

Additionally, we have included a DAG in the supplementary materials to make our assumptions about confounder relationship

- "The p-value of PE was adjusted with Benjamini-Hochberg (BH) adjustment (threshold of 0.05)." - This is appropriate and e
Thank you, we have added the clarification that 0.05 is the type I error threshold.

- "We included study participants' GA (GA)," - There is a double abbreviation here, maybe this is a typo?
Yes, we apologize for the typo. We have changed the first GA to its full name, gestational age.

- Please do not use "significant(ly)" as a standalone term. Rather, please use "statistical significance" or "clinical significance"
Thank you for this important suggestion. Indeed, it’s easy to misunderstand the term “significance”. We have changed all “sig

- "We did not include data from Kashimi et al. because there was no significant cpg before the confounder adjustment." - I do
analysis. Otherwise, you will obtain biased estimates.
We apologize for the confusion in our original wording. We agree with the reviewer that excluding a dataset based on statistic
Our actual reason for not including the Kashima et al. dataset was that it was generated from cord blood PBMCs, whereas all
appropriate.
We revised this sentence accordingly.
- "To harmonize the datasets, we applied the combat function to remove batch effects while preserving sample group informa
Thank you, we have changed all “combat” to “ComBat” throughout the manuscript.

- "resulting in an inevitable correlation of PE cases and smaller gestational age." - To make this more precise, I would say tha

Changed the original sentences to "resulting in an inevitable positive correlation of PE cases and smaller gestational age." as

- As a very minor comment, sometimes you write out gestational age, sometimes you write GA. Please be consistent (I sugge
Thank you, we have changed all GA in the manuscript back to gestational age, to ensure consistency.
- What is the reason for applying FWER correction in one part of your analytic pipeline and B-H FDR in another part? Is there

FWER appears only in our DMR analysis because the bumphunter algorithm outputs permutation-based, FWER-controlled p-v
methylation studies [1, 2]. Using FDR at the probe level ensures fair comparison of p-values across datasets and with the lite

[1] Ching T, Ha J, Song MA, et al. Genome-scale hypomethylation in the cord blood DNAs associated with early onset preecla
[2] Knihtilä HM, Kachroo P, Shadid I, Raissadati A, Peng C, McElrath TF, Litonjua AA, Demeo DL, Loscalzo J, Weiss ST, Mirzak
22. PMID: 37995466; PMCID: PMC10709000.

- It is very commendable that the authors made their code publicly available, amazing!
Thank you for the supportive comment.

Results

- "There is no significant difference (P > 0.05) in maternal age, parity, BMI, ethnicity, and smoking status." - Difference betw
We are referring to the difference between PE cases and controls, the detailed statistics(mean, standard deviation, p-values)
We added the clarification in the text "Maternal characteristics were similar between the preeclampsia and control groups, ex

- There is quite a bit of repetition in the Results related to key methodologies that were already explained in detail under Met
Thank you for the suggestion. We've shortened the first paragraph of the Results section and kept only the key details, so tha

- Where you present cell proportion differences between PE and control, you indicate "t" for some cell types, and "beta" for o
Apologies for the typo—these should all be "t," referring to the t-statistics from two-sided t-tests. We used two-sided t-tests f
We have revised the typo in the manuscript.

- "Since previous studies reported that maternal smoking significantly affects DNA methylation (43)." - This is not a full sente
Thanks for flagging this. We have made changes as suggested.

- "Since previous studies reported that maternal smoking significantly affects DNA methylation. To consider this we included t

Thank you. In the revision, we now make explicit that covariate selection is a priori and driven by domain knowledge rather t

Specifically, we select potential confounders based on prior literature and biological rationale: maternal smoking, maternal ag

Also, we adjust for estimated cell-type proportions to address cell-mixture bias in bulk cord-blood EWAS (standard practice),

- "In conclusion, we found that the observed DNA methylation variation among the whole cord blood samples is primarily ass
(and probable) that gestational age alone could have resulted in the same results. In fact, this would be the way to assess th
Thank you for the comment. We removed this sentence, given the updated results.

- Related: The analysis you undertook using the Herzog et al. data, which does not have GA information, shows that adjustm

Indeed, since gestational age (GA) information was not available in the Herzog et al. dataset, we could not directly evaluate c
possibility that GA may also underlie these results.

- Instead of adjusted P values only, it would also be important to make a note of the magnitude of association differences as
Thank you, we have added the magnitude of changes to the association between cell proportions and GA.

- In the Results, you use the term "insignificant" as an opposite to statistically significant. This is incorrect and should not be
Thank you, we have revised as suggested.

- After reading your Results and the parts related to the meta-analysis, I would like to emphasize again that I think excluding
selective selection based on results, which is big NO). Furthermore, it is also a statistical possibility that by adjusting for cell t

We appreciate your thoughtful comment and agree with your comments. As mentioned above, we still decided to exclude Kas
and have a substantially different overall cell composition from whole blood, they are not appropriate for the whole-blood bas
As suggested, we performed a differential methylation analysis on the Kashima et al. dataset, adjusting for estimated cell pro
rationale.
- "The proportions of monocytes also significantly increase as gestation progresses, after adjusting for other variables (p=0.0
We apologize for the error. This p-value was obtained by directly regressing the cell proportion of monocytes on gestational a
“The proportions of monocytes also significantly increase as gestation progresses (p=0.019)”

- "These trends of cell proportions are mostly the same in the case and control groups except for monocytes, which show a p
and methodology allow you to disentangle GA, cell proportions, and PE status.

Thank you for highlighting this important point. We agree that the potential interaction between preeclampsia (PE) and gesta
Additionally, in the new revision, PE and GA also has significant interaction in granocyte and nRBCs(Figure 4b). We have revis

- "None of the interaction terms between datasets and GA turn out to be significant, as the p-values in Fig. 5." - Please check
Thank you, we have revised the sentence to be “None of the gestational-age interaction terms were statistically significant (F

Discussion

- "In this study, we showed there is a lack of association between severe 401 PE and DNA methylation level changes, in the c
adjustments, which is THE key learning here.

Thank you, we revised this sentence to: "In this study, we showed there is a lack of association between severe PE and offsp

- "Instead, severe PE manifests itself impact on the offspring of the affected mother by altering the proportions of some immu
limited evidence that supports a statistical association between cell types and PE. One is that CD8T remains borderline statist
very robust; however, they are the key novel results that make your article stand out. I suggest some rewording to appropria
to study in further studies (and tone down overstated conclusions in THIS study).

We thank the reviewer for this important comment and agree that our original wording overstated the conclusions. We have r
borderline statistical association of CD8T cell proportions with severe PE, and (ii) a statistically significant interaction between

We have changed the sentences to “Our results suggest that severe preeclampsia may be linked to subtle shifts in offspring i

- "This observation is now expanded to their offspring, demonstrating the impact of PE." - This is a causal interpretation, whic

Thank you—we agree and have removed causal phrasing. We have rephrased this line.
This observation is now expanded to their offspring, indicating an association between maternal PE and neonatal DNA methyl
We also replaced similar terms (e.g., “impact,” “effect”) with “association/differences” throughout and added a note in the Di

- I completely agree with your conclusion related to the statistical considerations about proper adjustment for cell proportion
Thank you for the supportive comments.

- Related to Kazmi et al.: " However, they did not adjust for GA due to the concern of its confounding effect on PE." - This sou

Apologies for the confusion. We have revised this sentence accordingly “However, they did not adjust for gestational age, wh

- "We showed that GA affects the methylation pattern both directly and through cell proportion change, …" - I do not think yo
Following the comment, we revised this sentence to “Additionally, we show that gestational age is associated with cell propor

- "Instead, severe PE is associated with significant changes in several cell proportions in the cord blood." - This conclusion ov
Thank you for flagging this. We removed this sentence given the new updated results.

- I lacked a more structured highlight of the CD8T association (independent from GA), and the monocyte interaction effect. T
reading).

Thank you for the insightful observations. We have revised the preprocessing steps based on reviewer comments, and in the

Additionally, PE shows significant interactions with gestational age in more cells (granulocyte, monocyte and nRBC; Figure 4B
rather than central conclusions of this study.

To clarify this, we have added brief highlighting sentences in the Results and Discussion sections to note these associations a

- Further limitations of your study are that there is no information on key lifestyle variables, such as diet and physical activity
Thank you for the important suggestion. We have added this to our discussion section. "Lifestyle factors such as diet and phy

Items

For all items (and supplemental items), please always include abbreviations in the item legends. These are missing from a lot
"Cell_Type_Proportion_Residuals", which sounds like an R variable name; you can make these nicer. For each figure, please r
Thank you for the suggestions! Here are the changes we made accordingly:
Added missed abbreviations in item legends, including EWAS, PBMC, cell type to captions.
Fixed the texts on figures that don't read naturally. (Figure 2)
Replaced ns with exact p-values in Figure 2B, 2D, 3J, 4 and 5
We have changed the GA(PE) in Figure 5A to GA(Disease) to be consistent with previous figures.

Table 1: This table shows the sample characteristics. Due to the low sample size, I would suggest that the authors use nonpa
We have replaced t-tests with Wilcoxon test (nonparametric) to compare numeric variables.

Figure 1: This is a neat figure that shows the analytical pipeline.
Thank you.

Figure 2: The entire figure ended up very small in the downloaded PDF. Panel A) is very difficult to interpret, and not really re
significance, in the figure legend. Why not include exact P values? Panel C) is informative, but would remove the coloring as c

Thank you for the helpful suggestions regarding Figure 2. We agree that Panel A is not central to our results and have remov
confounders, and we explicitly indicate the modeling approach used. We also removed dashed lines from figure texts and incr

Figure 3. Panels A-D-G) Please remove the color. In the other panels, in the volcano plots, please remove the written-out CpG

We have removed the colors from SOV plots (Figure 3A, 3D, 3G). The cell proportion and test results shown in Figure J is not

Figure 4. These plots are great, but I would be more interested in the interaction magnitudes (GA x PE) and interaction P valu

Thank you, we have added the magnitudes and p-values for the interaction term between GA and PE.

Figure 5. I don't really understand the utility of these analyses. Why is it so relevant whether there is an interaction between

We apologize for any confusion. The objective of Figure 5 is to demonstrate that the association between cell proportions and
Kashima's CBMC results, stratified by sample group (preeclampsia vs. control). The interaction term tests whether the relatio
The PBMC label in the figure should be CBMC instead. We have fixed the typo.

Supplemental Items

Supplemental Figure 1. This is a very nice flowchart showing the epigenetic processing pipeline. This is a very technical descr
Thank you for the positive feedback on Supplemental Figure 1. This flowchart was designed to clearly illustrate the DNA meth

Supplemental Figure 2. This is related to the data quality control. This is a very technical description of the quality control pro

We appreciate the reviewer's feedback on Supplemental Figure 2.These are standard steps implemented in the ChAMP R pack

Supplemental Figure 3. Impact of smoking adjustment visualized using volcano plots, showing little to no impact. The only co

Thank you for the feedback. Since multiple reviewers suggested not to use the SOV method to select confounders, we have r
manuscript.

Supplemental Figure 4. Volcano plot showing differentially methylated sites in the Kashima data. All clear.

Thank you.

Supplemental Figure 5. I have the same comments for this figure as I already had for the main figure items.

Thank you for your comments. We have revised supplementary figure 5 following the same instruction you provided for the r

Supplemental Table 1. For each variable in the table (column 1), please include units and/or reference values (e.g., for ethnic
We have added the units for maternal age (years) and gestational age (weeks), as well as the reference category for ethnicit

Supplemental Table 2. These are the top 20 differentially methylated CpGs, but I am not sure what comparison this relates to
consumption". Please clean up this table so that all parts are understandable.

Thank you for the feedback. We are sorry for the confusion. Supplementary Table 2 includes the results of top 20 differentiall
and smoking). We added a description of each column in the table caption to improve readability.

"I previously reviewed this paper previously in Research Hub and you can read these comments via the Research Hub review
severe-
preeclampsia%2Freviews&data=05%7C02%7Clgarmire%40med.umich.edu%7C612fd2770dc44658fa8208ddc463354f%7C1f
data=CMBybQ%2B%2BXX17tXBMHuVPMBTff65eCRCYC6bOoTvJmDM%3D&reserved=0

The original preprint linked to the Research Hub review is here:
https://nam02.safelinks.protection.outlook.com/?url=https%3A%2F%2Fwww.medrxiv.org%2Fcontent%2F10.1101%2F2025
U1hcGkiOnRydWUsIlYiOiIwLjAuMDAwMCIsIlAiOiJXaW4zMiIsIkFOIjoiTWFpbCIsIldUIjoyfQ%3D%3D%7C0%7C%7C%7C&sdata=

Reviewer #2: The authors investigate DNA methylation differences between preeclampsia cases and controls. The main findir
considered to fall into the category of a "negative result". However, in my opinion findings like this provide a step forward in
differences in birth and pregnancy studies more generally.
Thank you for your comments, we appreciate that the reviewer concurs with us on the importance of reporting replicable "ne

I have some comments and proposed additions to the methods and results sections. However, in most cases these will be suc
Thank you!

Introduction:

The introduction seems clear and succinct. It introduces the problem, shows what has been done before, and highlights majo
proportions and other necessary covariates, such as gestational age.

The only suggestion I have for introduction is to consider mentioning the (exact or approximate) sample sizes of each previou
et. al.) only had 135 cases, which would not be considered "large scale". This should be turned into another item highlighting

Thank you for the suggestion! We have added the number fo preeclampsia cases to each mentioned study in the introduction

Methods:

The methods section is written very clearly, it depicts what has been done both in terms of data preparation and subsequent
missing BMI values. My only suggestion for the git repository would be to include a README file with some guidelines describ

Thank you, we have added a README file to the github repository as suggested. The file includes the version of R and each p

The section describing cohorts, sample preparation, and DNA methylation profiling are all excellent. It lists the sample sizes a
is to mention how the step of bisulphite conversion was done.
We used EZ DNA Methylation kit for the bisulfite conversion step (Zymo Research) as recommended by the Infinium Methylat

The data preparation steps can be improved.

First, it is not fully clear how the probes were filtered by detection p-values. The manuscript says that probes with detection p
had to have a low detection p-value)? In addition consider an alternative and more robust method for obtaining detection p-v
Thank you for the comment. We followed the ChAMP pipeline for filtering probes and samples based on detection p-values. S
probes. After that, probes that failed in any sample were also removed.
We have clarified these details in the revised Methods section under "DNA methylation data pre-processing and quality contro
We also examined the recommended non-specific fluorescence (NSP) method proposed by Heiss & Just (PMID 30678737), wh
larger number of undetected probes—approximately 69,517 in our dataset compared with 7,941 under the ChAMP criterion. A
ChAMP/minfi filtering strategy, which is widely used and more compatible with our analysis framework.

It is unclear what "removing probes with no match CpG site" mean. Are these non-CG (CH) probes as well as control (rs) pro

Thanks for pointing this out. Yes, by "removing probes with no matched CpG site" we referred to non-cg labelled probes(ch p
pipeline of the "ChAMP" package. We did not remove the rs probes (SNPs) from our analysis.

We agree that our original wording was unclear and have revised the Methods to specify that we removed ch probes.

Removing cross-hybridizing probes (that map to multiple locations in the genome) is clear. But based on the reported numbe
https://nam02.safelinks.protection.outlook.com/?url=https%3A%2F%2Fzwdzwd.github.io%2FInfiniumAnnotation&data=05%
MDAwMCIsIlAiOiJXaW4zMiIsIkFOIjoiTWFpbCIsIldUIjoyfQ%3D%3D%7C0%7C%7C%7C&sdata=%2BsS112HQBhChMSHuKr84I

Thank you for the suggestion. We have extracted the suggested new list of cross-hybridization probes and updated our prepr
filtered dataset has 803,359 probes in total (see updated supplementary figure 1). This change doesn't affect our conclusions

The normalization was done with BMIQ, which adjusts the intensities between Type I and Type II probes. I think this is a goo

Thanks for the comment. While it is true that our downstream analyses primarily compare samples within the same probe, no
comparability across samples. The BMIQ method specifically corrects for probe-type bias while preserving biological variation
pipelines[3], and we believe it cannot be omitted even if our primary comparisons are sample-level rather than probe-type le

[1] Andrew E. Teschendorff, Francesco Marabita, Matthias Lechner, Thomas Bartlett, Jesper Tegner, David Gomez-Cabrero, S
org.proxy.lib.umich.edu/10.1093/bioinformatics/bts680
[2] Wang, Z., Wu, X. & Wang, Y. A framework for analyzing DNA methylation data from Illumina Infinium HumanMethylation
[3] Welsh, H., Batalha, C.M.P.F., Li, W. et al. A systematic evaluation of normalization methods and probe replicability using i

ComBat was used in order to deal with potential batch effects. I would recommend against using ComBat as it has been repe
sentrix plates they can be controlled in the same way as cell type proportion effects - by including them in the model. Furthe

Thank you for the insightful suggestion. We have updated the data processing pipeline by removing the ComBat batch correc
accordingly.

Cell type deconvolution step is a bit unclear. The manuscript states that it used a reference by Gervin et. al. and in the softwa
performed should be provided.

We only used the cell type deconvolution functions from EpiDISH package. By the time we wrote this manuscript, there were

We have explained the deconvolution pipeline in the method section under "cell type deconvolution" and the "software usage

In addition, the newer version of EpiDISH has a "uni life" reference (centUniLife.m object) which contains 19 cell types (cord

7&data=05%7C02%7Clgarmire%40med.umich.edu%7C612fd2770dc44658fa8208ddc463354f%7C1f41d613d3a14ead918d2a
K3Kr8nECffm8fxmMBsvG5RKds11A%3D&reserved=0). It would be interesting to test if this reference would allow a more gra
Thank you for the suggestion. The study by Guo et al. developed the "UniLife" reference by combining 7 cord blood cell types
manuscript. Even though Guo et al.'s reference includes more total cell types, the additional adult cell types are less relevant
We applied the UniLife reference to our own cord blood data. As expected, most adult cell types were not detected. However,
keep Gervin et al.–based reference.

The strategy for selecting clinical confoudners raises a small concern. The effects were averaged across all investigated CpGs

Thank you, this is a very important suggestion. We have changed the analysis so that all confounders (clinical variables, cell p

The volcano plots in figure 3 have been updated to reflect this change. The change doesn't affect previous results.

For EWAS the limma package is used in order to perform a modulated analysis which pools the variance estimates across ma
Thank you for your supportive comment.

The meta analysis and combination of cohorts again uses ComBat in order to remove batch effect. As mentioned previously e
model supplied to limma.

Thank you for raising this important point. We have carefully addressed this concern by excluding ComBat in each individual c
ComBat because the differences among cohorts are likely non-linear and may not be fully captured by including cohort as a d

Including additional preterm samples as controls seems like a good idea. But it is uncler why there is an additional correction

Thank you for the comment. We applied bacon only to the analysis that included idiopathic preterm samples because it showe
widely agrees that an inflation score > 1.2 is problematic and needs to be adjusted.

All other analyses have inflation score within normal range: for in-house data, the final model has λ = 0.922; for Ching et al.

However, for the analysis with idiopathic preterm samples, the λ = 1.383. It's recommended that a bacon adjustment be mad

For the detection of differentially methylated regions the authors used "bumphunter" which is a well known algorithm. The se
specified as well.

Thank you for the suggestion! We have added the detailed choice of parameters for "bumphunter" function in the method sec
parameter choice see "5.3 - DMR.R" in the code). "

Results:

The section about cell proportion variability across clinical variables is clear and well examined. One comment here is that it w
We apologize for not being able to incorporate this new information. We tried to use UniLife to deconvolute our data, but coul
We have added the need of more fine grid cord blood reference in the discussion section. "Now, cord blood references only co

It also seems relevant to check whether the same patterns of cell type proportion difference could be reproduced in other coh
Thank you for this helpful suggestion. We did examine the cell type proportion difference in the pooled cohort and presented
However, we were unable to generate fully confounder-adjusted boxplots for the public datasets, as they al have limitations:
samples were PBMCs (thus lacking granulocytes). For this dataset, we compared PE and controls without adjustment for othe

I would also recommend one additional step to make sure that cell type heterogeneity is the sole cause of PE DNA methylatio
sizes a more complex model will be severely under-powered. Hence, it could be suspected that the lack of significant differen

Briefly, I suggest investigating the cytosines reported as significantly associated with PE (when no adjustment for confounder
heterogeneity this analysis will reveal that the effect sizes became a lot smaller and the directions of effects are no longer co

Thank you for the suggestion! We plotted the effect sizes of CpGs before and after confounder adjustment. We used t-statisti
adjustment are indeed caused by cell type heterogeneity.

We have added this new result to result section and supplementary figure 5.

An additional analysis that would be really interesting to see is whether the original EWAS hits were not caused by a mismatc
overlap with the cytosines reported to be different between PE and controls. This would strongly indicate that one of the caus

Thank you for this insightful suggestion. We performed the proposed analysis by computing differentially methylated probes l
dataset (analyzed in the same manner, without confounder adjustment), 19,297 of the 35,936 significant probes overlapped

Both datasets have slightly over 50% overlaps with the preterm vs fullterm probes, which supports the interpretation that ge
effect may also contributes to the biases in the previous studies.

Discussion:

The discussion is very professional and clear. It highlights the issue of cellular heterogeneity and lists examples of this issue i

One thing I would like to see added here is a discussion about more granular cell type decomposition and how that could play
We apologize for not being able to incorporate this new information. The naïve and memory B-cell signatures in the UniLife re
We have acknowledged in the Discussion that investigating finer-grained cell proportion changes remains an important direct
Another thing I would like to see added is a point about the broader context of the findings. The issues investigated by the au

Thank you, this is a very valuable suggestion. We have added a sentence in the discussion section to expand our conclusion t

Other:

Some wording and sentences

1. "We validated this lack of CpG changes using multiple published cord blood methylation datasets."

Should be more precise. These are not "CpG" changes but cytosine methylation changes.

Thank you for pointing this out. We agree that 'CpG methylation change' is not precise, because methyl groups are located o
methylation changes'.

2. "Cord blood consists of many diverse cell types, each with a distinct epigenome profile as defined."

Unclear what the last two words "as defined" are trying to say. I think they should be removed?

We have removed the redundant "as defined" from the sentence.

3. SVD stands for "Singular Value Decomposition", not "Singular Vector Decomposition".
Thank you, we have corrected this.

Some comments about the figures:

Figure 1. Study overview and experiment design.

All clear and nicely presented.

Thank you for your supportive comments.

Figure 2. Cell types in samples.

The samples in heatmap are currently clustered by granulocyte count, which is not that interesting. Consider arranging the sa
We removed figure 2A as suggested by another reviewer, because it's less informative.

Figure labels (in boxplots) have underscores in them.
We have removed the underscores in the labels.

Figure 3.

The title of this figure is a bit long, I would move the part about cohorts and figure letters into the caption text instead.
We have moved the cohorts into the caption as suggested.

Some figure annotations contain underscores.
We have removed the underscores in annotations.

The "p-value" should not be capitalized (in volcano plots).
Thank you, we have revised to use lower-case p in "p-value".

Figure 4. Cell type proportions in relationship with gestational age.

I would recommend showing the actual p-values instead of writing NS. Some readers might want to draw their own conclusio
Thank you, we have added actual p-values for all cell types.

Figure 5. Cell proportions in relationship with gestational age are coherent in two different datasets

The term "P-interaction" is a bit awkward, consider using "interaction p-value" instead.
Thank you, we have changed "P-interaction" to "interaction p-value"

Supplementary Figure 1.

All clear.

Supplementary Figure 2.

Density plot B is said to be a result of density plot (A) after removing a single sample. However, for some reason, seems like

We apologize for the earlier mistake. The difference between plots A and B is due to data normalization and the removal of or

We have revised the caption for supplementary figure 2 to include this revision.

Supplementary Figure 4.

"p-value" should not be capitalized.
Revised.

Supplementary Figure 5.

Some underscores in figure labels, and CamelCase ("CellType") for boxplot.
Revised.

Table 1.

All clear.

Supplementary Table 1.

All clear.

Supplementary Table 2.

All clear.

Good study, I had a good time while reading your manuscript and read it with interest. Hope this is helpful.

P.S. I previously reviewed this paper previously in Research Hub and you can read these comments via the Research Hub rev
with-severe-
preeclampsia%2Freviews&data=05%7C02%7Clgarmire%40med.umich.edu%7C612fd2770dc44658fa8208ddc463354f%7C1f
data=1o%2FJPsR%2F9A8ORU16VtYXj5bV9QKQNkrhQ5IGK61z8HY%3D&reserved=0

Reviewer #3:

Summary
This study investigates the association between cord blood DNA methylation and pre-eclampsia using both original data and s
pre-eclampsia cases.

Comments

1. Main comments
- The authors tested associations between estimated cell type proportions and case/control status or gestational age using lin
compositional regression models).

Thank you for the important advice. Indeed, including all cell types in one regression model would be a violation of the assum
(centered log-ratio transformation) or dropping one column because ILR gave more stable and statistically valid result.

We updated all the affected analyses. See method section under "CpG-level epigenome-wide association analysis (EWAS)"

- To explore DNA methylation changes within specific cell types in relation to pre-eclampsia, it would be valuable to perform a
https://nam02.safelinks.protection.outlook.com/?url=https%3A%2F%2Fwww.nature.com%2Farticles%2Fs41467-020-18618
y&data=05%7C02%7Clgarmire%40med.umich.edu%7C612fd2770dc44658fa8208ddc463354f%7C1f41d613d3a14ead918d2a
UjfWB%2B0k2fpJQk3aBfVIBInzzuAM%3D&reserved=0.

We did attempt to apply the cellDMC algorithm, as described in the referenced study, to test for interactions between PE stat
Results section to document this attempt and its outcome.

- Could the authors estimate the proportion of the association between pre-eclampsia and cell type proportions that is mediat

Thank you for your suggestion. We have included a mediation analysis as suggested.

Below is the effect size of direct, indirect and total effect of this mediation analysis:

From the figure, the indirect effect(GA--cell proportion change) is the main drivers for most cell types, the only exception bei

We added this analysis to the result section under "Associations between cord blood cell types and severe preeclampsia" and

2. Abstract

- "Previous epigenome-wide association studies (EWAS) on PE have produced inconsistent results, possibly due to inadequate
The inconsistency is observed in cord blood samples. We have added the information to the abstract.

- Authors should add sample size of the own and public data analyzed in the study.

Thank you for the suggestion. We have added the sample size for our in-house data and other mentioned public datasets in t

- "We validated this lack of CpG changes using multiple published cord blood methylation datasets." Authors should change to

Thank you. We have removed the redundant sentence "We validated this lack of CpG changes using multiple published cord b

- In the summary, authors should state clear that they talk about cord blood and not other tissues.

We added the specification of the cord blood tissue type in the summary part.

3. Methods
- The datasets analysed in the manuscript use different pre-processing pipelines. While this is fine for cases and controls proc
process these controls following the same pipeline as for the cases.

Thanks for pointing this out. For the idiopathic preterm cohort, Fernando et al. mentioned in the Sentrix file that they used C
the processed beta matrix directly, merge it with our data and normalize the merged dataset.

- In the section "Cell-type deconvolution in umbilical cord whole blood (CB)", authors should clarify if this was done for all the
Thank you, we have added a clarification that the same cell type deconvolution process was done to all datasets(in house and

- Authors should indicate the type of meta-analysis done (fixed effects? Weighted-variance?).

We apologize for the earlier mislabeling. Our approach integrates the public datasets at the raw data level (beta matrices) so
maximizes statistical power. Given that we had access to individual-level data from each cohort, we considered pooled analys

We have changed all "meta-analysis" in the manuscript to "pooled analysis".

- Authors should give more details about this point "To harmonize the datasets, we applied the combat function to remove ba

By "harmonize the datasets," we mean that the raw methylation data from our in-house cohort and the public datasets were
an empirical Bayes framework to adjust for additive and multiplicative batch effects while preserving biological variation relat
under study.
We have revised the Methods to include these details under the "Pooled-analysis using three datasets" subsection.

- "Lastly, we calculated differentially methylated probes again, with adjustment to the sample group and estimated cell types

We apologize for the confusing wording. It should be "with adjustment to estimated cell types and plotted another volcano pl

We have revised the sentence.

- "We did not include data from Kashimi et al. because there was no significant cpg before the confounder adjustment." I thir

Thank you for pointing this out. Another reason we did not include Kashima's dataset is that, unlike the other three cohorts, i
methylation analyses, this difference would likely introduce substantial bias if the datasets were simply pooled. We have revis

- Why is the model of idiopathic preterm controls corrected using "bacon" but not the others? It sounds inconsistent. I would
Thank you for the comment. We applied bacon only to the analysis that included idiopathic preterm samples because it showe
widely agree on an inflation score > 1.2 to be problematic and needs to be adjusted.

All other analysis have inflation score within normal range: for in-house data, the final model has λ = 0.922; for Ching et al.

However, for the analysis with idiopathic preterm samples, the λ = 1.383. It’s recommended that a bacon adjustment to redu [illegible]
method section.

- For the DMR I would suggest to use more than one tool and keep the overlap as final results as there are considerable diffe [illegible]

Thank you for the suggestion. We used bumphunter and dmrff algorithms to detect significant DMR. Unfortunately, although [illegible]
DMRcate, probeLasso, or comb-p. Unfortunately, all these methods require a significant CPG list as an input. Since we don’t h [illegible]

4. Results
- I would delete some of the text referring to methods from the results section. (ie. I would delete lines 263-270, and 273-27 [illegible]
Thank you. We have removed some redundant text in the first paragraph of the result section.

- In Figure 1 add "gene-based EWAS" and "DMR" analyses
Fixed

5. Discussion
- "We did not detect significant differences in the inferred proportion of endothelial cells between the control and PE groups u [illegible]

The study using paired placental samples is still ongoing. We unfortunately don’t have a preprint or paper out yet. We will rer [illegible]

Minor comments
1. Define variables the first time they appear (ie. BMI in the abstract)
Revised.
2. Correct typos (ie. Preeclampsia(PE) - space missing)
We have ensured all parentheses have space in front of them.
3. Human gene names in cursive (ie. AVPR1a, OXTR, and .PKCB)
All gene symbols have been reformatted.
4. Change "didn't" to "did not".
Fixed.
5. Correct: "... (GSE103253). and (3)..."
We removed “and”.
6. Correct: "...cpg..."
Fixed
7. Keep naming of "combat" consistent along the text.
We have changed all “combat” to “ComBat”.
8. In the sentence "... related to Rheumatoid Arthritis after...", change disease name to non-capital letters.
Fixed
9. Change "Causcasian" for "European origin / ancestry".
Revised

Editorial Manager®

Cookie Preference Center

We use cookies which are necessary to make our site work. We may also use additional cookies to analyze, improve and personalize our content and your digital experience. For more information, see our Cookie Policy.

You may choose not to allow some types of cookies. However, blocking some types may impact your experience of our site and the services we are able to offer. See the different category headings below to find out more or change your settings.

Allow all

Manage Consent Preferences

Strictly Necessary Cookies

Always active

These cookies are necessary for the website to function and cannot be switched off in our systems. They are usually only set in response to actions made by you which amount to a request for services, such as setting your privacy preferences, logging in or filling in forms. You can set your browser to block or alert you about these cookies, but some parts of the site will not then work.

Functional Cookies

These cookies enable the website to provide enhanced functionality and personalisation. They may be set by us or by third party providers whose services we have added to our pages. If you do not allow these cookies then some or all of these services may not function properly.

Performance Cookies

These cookies allow us to count visits and traffic sources so we can measure and improve the performance of our site. They help us to know which pages are the most and least popular and see how visitors move around the site.

Targeting Cookies

These cookies may be set through our site by our advertising partners. They may be used by those companies to build a profile of your interests and show you relevant adverts on other sites. If you do not allow these cookies, you will experience less targeted advertising.

Cookie List

Clear

Apply Cancel
Consent Leg.Interest

Confirm my choices

Powered by onetrust
