# Supplementary material for: Cord blood DNA methylation and cell-type composition are not significantly associated with severe preeclampsia after cell-type and clinical covariate adjustment
Source: Gigascience. 2026 Jan 16;15:giag002. doi: 10.1093/gigascience/giag002 (PMC13014471; doi:10.1093/gigascience/giag002)
Supplement: giag002_Reviewer_1_Report_original_submission — Tibor Varga -- 7/14/2025 [file giag002_reviewer_1_report_original_submission.pdf]

Reviewer Report

Title: Cord Blood DNA methylation and Cell Type composition are not significantly associated with severe preeclampsia, after confounding adjustment

Version: Original Submission **Date:** 7/14/2025

Reviewer name: Tibor Varga

Reviewer Comments to Author:

Credentials
I am an Associate Professor of Epidemiology and Health Equity at the Department of Public Health at the University of Copenhagen. I have been reviewing articles for over a decade for >30 biomedical journals, and I have been a key reviewer and statistical reviewer at the journal Diabetologia every year since 2020, with around 10 reviewed manuscripts each year for the past couple of years, with excellent editorial feedback. I obtained Clarivate Web of Science Academy certificates in Scientific Peer Review. My expertise is in cardiometabolic disease epidemiology, genetic epidemiology, biostatistics, machine learning, artificial intelligence, algorithmic fairness, and health inequalities. I have significant experience authoring and reviewing papers related to various omics studies, including genomics, epigenomics, and lipidomics. I am familiar with epigenetic association analysis pipelines and have reviewed, e.g., several papers related to epigenomics clocks. I also have the necessary expertise to assess the study design and the statistical methodology of this paper. In fact, I have analyzed omics data using similar pipelines, and am familiar with some of the bioinformatics toolkits utilized in this paper (e.g., limma, pathway analysis tools). No AI technologies were used in the writing of this review other than a standard spell checker to correct typos.
Overall
I read the study by Yang X. et al. with interest. I was particularly interested in this study due to its dual aims: I really liked that the study had a strong biological hypothesis, but also aimed to highlight the importance of methodological considerations related to confounder adjustment. Based on my assessment, I see the second aim (statistical) fully delivered, but the first aim (biological/causal) potentially misleading or overstated. I fully agree with the authors in their final message that all studies should pay attention to proper confounder adjustment, including clinical variables and also cell proportions, when it comes to epigenomics analysis. However, the authors themselves fell into a trap of getting a bit lost in adjustments and not properly considering confounders. First, it appears that confounder selection was automated based on statistical relationships, which shows the lack of thought put into appropriately selecting these based on a priori hypotheses (e.g., via drawing up a DAG). Second, the results are often misinterpreted. The authors claim a causal relationship (perhaps via poorly chosen wording) in the Discussion, and seem to conclude that cell proportions are key drivers in this study, whereas to me it appears, based on the results, that gestational age is the strongest correlate with DNA methylation patterns, rather than cell proportions. In my opinion, the following key findings emerged here, and this would be a logical order for presentation:
1) Preeclampsia strongly associates with gestational age (known);

2) Cell proportions strongly correlate with gestational age (known);
3) CD8T cell proportions associate with preeclampsia even after gestational age adjustment (main effect, key novel finding);
4) Monocyte cell proportions show differential association with gestational age according to preeclampsia status (interaction, key novel finding).
Then, I would suggest the authors dive deeper into a "causal ideation": yes, DNA methylation associations disappear after adjustment for gestational age (and perhaps cell proportions), but this does not mean that they are not important. In fact, they can still be causal drivers of outcomes! But a more in-depth exploration into what comes first: the disease, the methylation, altered cell proportions, and what impacts what, would be a real added value to the Discussion of this paper. Please see my detailed comments below, hope they are helpful.
Introduction
- I suggest that you do not abbreviate preeclampsia as PE. It does not change the word count, and it is easier to read the full word out and avoid an unnecessary abbreviation.
- You mention that the Kazmi et al. article reported certain findings "after adjusting for cell types". Does this mean cell proportions, or how does this adjustment normally happen?
- "(Kazmi et al) However, their results did not adjust for the gestational age, the major confounder of preeclampsia" - This sentence needs expansion. A confounder between what two factors? Can you explain how gestational age is a confounder in this setting (with respect to the key statistical criteria for being a confounder)?
- The Introduction is excellent in clearly outlining a problem statement of poor confounder adjustment in epidemiological studies. I fully agree with the authors that this is often ignored in these studies. Key references are listed, showing a pattern of ignoring key confounders.
- Perhaps some more information in the Introduction on source tissues would be helpful to the reader. Are all the referred studies analyzed cord blood or rather maternal blood? What are the expected differences in results given tissue sources?
- "It is therefore essential to account for such heterogeneity, to improve the accuracy and sensitivity, and avoid biased conclusions" - Accuracy and sensitivity of what? From your problem statement and Introduction, my understanding is that this study relates to causal/biological discovery and highlighting methodological limitations (re: confounder adjustment). Accuracy and sensitivity are terms related to predictive statistics that have not been mentioned before, so these terms feel out of place. Besides, I do not think your study design is helpful for prediction.
- "Particularly, to ensure that any differences in DNA methylation are due to confounding factors, the analysis needs to be adjusted for cell proportions" - I think you meant to say that "to ensure that differences in DNA methylation are NOT due to confounding factors", right?
- "In this study, we pay special attention to these issues to seek a plausible epigenomic association between severe PE and cord blood of offspring from PE patients." - This reads a bit clumsily, and wording should be changed to more accurately describe the goals of your study. Associations are not sought after "between PE and cord blood", but PE and various epigenetic and cellular markers IN cord blood.
- I am surprised that eclampsia is not mentioned in the Introduction as an even more severe condition. Is there any evidence related to the associations between epigenetic markers and cellular markers and

eclampsia?

Methods

- Please write out "OBGYNs" in full words at first mention.
- "Severe PE was characterized by OBGYNs at Kapiolani Medical Center as sustained pregnancy induced hypertension(systolic/diastolic blood pressure >= 140/90) with urine protein and/or organ dysfunction."
- First, what do you mean by organ dysfunction? Which organ? Second, my read is that both elevated BP and proteinuria are needed for this diagnosis, but organ dysfunction is optional. Or is it that either "elevated BP-proteinuria" or "elevated BP-organ dysfunction" combinations are satisfactory? Please clarify.
- "…and had cord blood samples remaining in the HiBR" - So your inclusion criterion is that there are samples remaining. Can this selection process introduce selection bias? I.e., for what reasons are cord blood samples removed from HiBR? If this is related to certain outcomes or baseline characteristics of the participants, then there is a selection mechanism that should be acknowledged, and the process's impact should be considered.
- "We evaluated sample integrity, purity, and concentration on the Nanodrop and removed samples of low quality." - What is the Nanodrop? How was low quality defined, and according to what metrics?
- It would be very useful to present a directed acyclic graph (DAG) or a causal loop diagram (CLD) at some point in the manuscript. This could already happen in the Introduction stage, where you present the problem statement and your scientific hypothesis. This could formalize your hypothesis and also help you ascertain the confounder structure around the investigated statistical hypotheses. For some of the variables you chose to adjust for, I cannot immediately see whether they are potential confounders or mediators/colliders (in which case you should not adjust), so I suggest that you visualize everything with the DAG, and that you actually write out the three criteria for being a confounder in the Methods for the reader. I know that this is basic information, but this is key information for your manuscript that should be well understood by every reader of your paper to be able to fully appreciate the results (and who knows, you might uncover some new information through the visualization that you have not considered?).
- "The demographic and clinical information of the patients was collected and analyzed to identify any potential confounding effects." - What do you mean by this sentence? How was this done?
- "To validate the observations in the HiBR cohort, we did an exhaustive search among published work of cord blood (including PBMC) DNA methylation in association with PE (16,17,23)" - I suggest that you include a brief supplementary document on how you undertook this "exhaustive search". What does this term mean? Is this a systematic review? Did you establish a search term or involve a librarian? You refer to three papers, but it is unclear how these articles were identified.
- "baby gender, and baby birth weight" - While, of course, this is understandable, I am wondering if "baby" is scientifically appropriate. I suggest replacing this with "infant".
- "including 10 early-onsets and 13 late-onsets" - You previously introduced abbreviations for these: EOPE, LOPE. I suggest you either stick to the abbreviations or remove the abbreviations altogether.
- "For Herzog EM et al and Kashima K et al datasets, we directly used the beta matrix deposited to GEO."
- Please explain what the "beta matrix" is (I am assuming these are the summary level effect estimates), and what GEO stands for.
- "To disentangle the effect of PE and small GA" - Please refer to this as associations, as effect implies

causality, which cannot be demonstrated by these results. In general, please be mindful of this throughout the manuscript and refer to all observed results as associations, and not effects. Even for betas, I would prefer "magnitude of association", rather than "effect size".
- "idiopathic preterm birth samples" - What does idiopathic mean? Please explain for the general readership.
- For Fernando et al, you report: "We directly used the normalized matrix deposited to GEO." - Above, for Herzog and Kashima, you just referred to this as the "beta matrix". Is there a difference between the two (i.e., one is normalized, the other one not)?
- At first mention, please write out the full names of CD4T and CD8T cells.
- For all potential confounders listed under "Clinical confounders and source of variance analysis", please list categories and precise definitions. E.g., is BMI pre-pregnancy BMI (and when was it measured)? How is smoking status defined (is it during pregnancy, before pregnancy, what are the categories)? What are the categories for ethnicity?
- "We performed the source of variance (SOV) analysis on these clinical variables and previously estimated sample cell proportions to identify important confounding variables that need to be adjusted, as done before." - Despite the inclusion of three references at the end of this sentence, this needs clarification. You explain a statistical process after this sentence, implying that confounders were selected purely based on statistical relationships. This is not appropriate. Confounders should be selected based on a priori assumptions about whether something is a confounder or not. The three criteria for being a confounder are: 1) The variable must be statistically associated with the exposure. 2) The variable must cause the outcome. 3) The variable must not be on a causal pathway. As suggested above, I would much prefer that you visualize your investigated association using a DAG and determine confounders based on that exploration.
- "The p-value of PE was adjusted with Benjamini-Hochberg (BH) adjustment (threshold of 0.05)." - This is appropriate and established in the literature. I suggest that you clarify that the threshold is an alpha type 1 error threshold.
- "We included study participants' GA (GA)," - There is a double abbreviation here, maybe this is a typo?
- Please do not use "significant(ly)" as a standalone term. Rather, please use "statistical significance" or "clinical significance", depending on the context. The term has been massively overused in biomedical literature, with a generally poor understanding among the readership.
- "We did not include data from Kashimi et al. because there was no significant cpg before the confounder adjustment." - I do not understand this justification, and this is potentially a flawed approach. My assessment is that all samples, regardless of the statistical significance of original associations (with or without adjustment), should be included in the meta-analysis. Otherwise, you will obtain biased estimates.
- "To harmonize the datasets, we applied the combat function to remove batch effects while preserving sample group information" - Please correct this to "ComBat". There are further mentions of this in the manuscript, please double-check consistent naming.
- "resulting in an inevitable correlation of PE cases and smaller gestational age." - To make this more precise, I would say that there is a positive correlation between PE status and smaller gestational age.
- As a very minor comment, sometimes you write out gestational age, sometimes you write GA. Please be consistent (I suggest writing it out).

- What is the reason for applying FWER correction in one part of your analytic pipeline and B-H FDR in another part? Is there a specific reason for correcting for false positive rates vs. false discovery rates in the two analytic processes?
- It is very commendable that the authors made their code publicly available, amazing!

Results

- "There is no significant difference (P > 0.05) in maternal age, parity, BMI, ethnicity, and smoking status." - Difference between what groups? Please be explicit.
- There is quite a bit of repetition in the Results related to key methodologies that were already explained in detail under Methods. These could be removed to shorten the Results section (e.g., around 2/3 of the first paragraph of the Results is repetition, referring to the same supplemental material files that you already referred to in the Methods).
- Where you present cell proportion differences between PE and control, you indicate "t" for some cell types, and "beta" for other cell types. What motivates this inconsistency? What do these represent? Is the scale here % difference between the two groups (if so, please state the unit explicitly).
- "Since previous studies reported that maternal smoking significantly affects DNA methylation (43)." - This is not a full sentence, please combine it with the next one.
- "Since previous studies reported that maternal smoking significantly affects DNA methylation. To consider this we included the variable "smoking" despite its smaller effect on data variance." - This is precisely what I was referring to above at confounder adjustment selection. This is a more appropriate way of selecting potential confounders for adjustment.
- "In conclusion, we found that the observed DNA methylation variation among the whole cord blood samples is primarily associated with cell type differences rather than severe PE." - According to your analysis, this is an incorrect interpretation. You have no proof of that, as you adjusted the model for a range of other variables as well. E.g., it is entirely plausible (and probable) that gestational age alone could have resulted in the same results. In fact, this would be the way to assess this. To run parallel models and see whether the GA adjustment impacts the results, first. If differentially methylated sites remain, then you could assess whether cell proportions impact these associations.
- Related: The analysis you undertook using the Herzog et al. data, which does not have GA information, shows that adjustment for cell proportions alone makes all statistically significant sites disappear. So this is an interesting finding, but unfortunately still doesn't exclude the possibility of GA being the driver of these results.
- Instead of adjusted P values only, it would also be important to make a note of the magnitude of association differences as well.
- In the Results, you use the term "insignificant" as an opposite to statistically significant. This is incorrect and should not be used. Please use "not statistically significant".
- After reading your Results and the parts related to the meta-analysis, I would like to emphasize again that I think excluding Kashima et al from the meta-analysis is not appropriate, and will bias your findings (just because they have null results before cell proportion adjustments, this does not mean that they should not be added to the meta-analysis, this is selective selection based on results, which is big NO). Furthermore, it is also a statistical possibility that by adjusting for cell type proportions, some differentially methylated sites BECOME statistically significant, further illustrating the flawed logic here.
- "The proportions of monocytes also significantly increase as gestation progresses, after adjusting for

other variables (p=0.019)." - What are the adjustments here, in these analyses?
- "These trends of cell proportions are mostly the same in the case and control groups except for monocytes, which show a potential interaction effect between PE and gestational age." - THIS is perhaps your most important finding, sort of hidden in Results; you should emphasize this more!! Please include the magnitude of interaction and the P value. This result and methodology allow you to disentangle GA, cell proportions, and PE status.
- "None of the interaction terms between datasets and GA turn out to be significant, as the p-values in Fig. 5." - Please check grammar in this sentence.
Discussion
- "In this study, we showed there is a lack of association between severe 401 PE and DNA methylation level changes, in the cord blood samples of the offspring of these PE patients from multiple cohorts." - This is the first sentence of the Discussion section, but this statement is not exactly true, as this finding is only true when you explicitly mention the adjustments, which is THE key learning here.
- "Instead, severe PE manifests itself impact on the offspring of the affected mother by altering the proportions of some immune cell types in the blood." - This is a misleading and flawed conclusion. First, you cannot conclude a causal relationship. Second, this association is very, very likely to be almost completely explained by gestational age. There is only very limited evidence that supports a statistical association between cell types and PE. One is that CD8T remains borderline statistically significantly associated with severe PE vs. control status after adjustment for GA, and the second is the observed statistically significant interaction for monocytes between PE and gestational age. I would not consider these two results very robust; however, they are the key novel results that make your article stand out. I suggest some rewording to appropriately show these interesting findings, but in a way that rather emphasizes these as emerging novel hypotheses to study in further studies (and tone down overstated conclusions in THIS study).
- "This observation is now expanded to their offspring, demonstrating the impact of PE." - This is a causal interpretation, which you should avoid.
- I completely agree with your conclusion related to the statistical considerations about proper adjustment for cell proportion in these kinds of analysis. This is backed by your findings.
- Related to Kazmi et al.: " However, they did not adjust for GA due to the concern of its confounding effect on PE." - This sounds like a very strange reasoning, as "concern of confounding" is exactly the reason for an adjustment.
- "We showed that GA affects the methylation pattern both directly and through cell proportion change, …" - I do not think you can conclude these causal interpretations.
- "More importantly, the cell proportions in severe PE case groups are also affected by the disease itself, making them critical confounders in EWAS studies." - This is statistically not correct. One of the criteria for being a confounder is that the variable must cause the outcome. If the outcome causes the variable, then it might be a collider, which you should not adjust for.
- "Instead, severe PE is associated with significant changes in several cell proportions in the cord blood."
- This conclusion overstates the results.
- I lacked a more structured highlight of the CD8T association (independent from GA), and the monocyte interaction effect. These two key pieces of evidence should be emphasized a bit more, as the most important indications of potential cell proportion-related findings in terms of PE (the rest of the results

reflect cell proportion changes according to GA, in my reading).
- Further limitations of your study are that there is no information on key lifestyle variables, such as diet and physical activity, which both can have an impact on methylation patterns.
Items
For all items (and supplemental items), please always include abbreviations in the item legends. These are missing from a lot of items (cell types, EWAS, RE, etc.). For all figures, please edit the axis names, grouping names, and category names from the raw output from R to human-understandable names. E.g., the Figure 2D y-axis name is "Cell_Type_Proportion_Residuals", which sounds like an R variable name; you can make these nicer. For each figure, please replace "ns" with the exact P values (e.g., an adjusted P value of 0.051 would be ns, but informative).
Table 1: This table shows the sample characteristics. Due to the low sample size, I would suggest that the authors use nonparametric tests for the comparisons.
Figure 1: This is a neat figure that shows the analytical pipeline.
Figure 2: The entire figure ended up very small in the downloaded PDF. Panel A) is very difficult to interpret, and not really related to the results in the paper; you never mention hierarchical clustering in the paper, so I would remove this plot. Panel B) is informative; however, it does not explain how different numbers of stars reflect different levels of statistical significance, in the figure legend. Why not include exact P values? Panel C) is informative, but would remove the coloring as colors here do not include extra information. Panel D) is informative - are these confounder-adjusted means?
Figure 3. Panels A-D-G) Please remove the color. In the other panels, in the volcano plots, please remove the written-out CpG names; these are irrelevant for this study. Panel J) Are these confounder-adjusted means?
Figure 4. These plots are great, but I would be more interested in the interaction magnitudes (GA x PE) and interaction P values than the main associations between GA and cell proportions. Again, these are your key results in my opinion.
Figure 5. I don't really understand the utility of these analyses. Why is it so relevant whether there is an interaction between PBMC and WB? The previous interaction plot (Figure 4B) was great, but I do not get the utility of this one.
Supplemental Items
Supplemental Figure 1. This is a very nice flowchart showing the epigenetic processing pipeline. This is a very technical description of the flow, which goes beyond my expertise, so I am unable to ascertain whether this is fully appropriate or not.
Supplemental Figure 2. This is related to the data quality control. This is a very technical description of the quality control process, which goes beyond my expertise, so I am unable to ascertain whether this is fully appropriate or not.
Supplemental Figure 3. Impact of smoking adjustment visualized using volcano plots, showing little to no impact. The only comment I have here is to include in the legend that the red color indicates statistical significance (and looking at this plot, the reader can see that there are no statistically significant CpGs in either panel).
Supplemental Figure 4. Volcano plot showing differentially methylated sites in the Kashima data. All clear.
Supplemental Figure 5. I have the same comments for this figure as I already had for the main figure

items.
Supplemental Table 1. For each variable in the table (column 1), please include units and/or reference values (e.g., for ethnicity, what is the reference category). Please include units for the cell proportions as well. Are these %?
Supplemental Table 2. These are the top 20 differentially methylated CpGs, but I am not sure what comparison this relates to, and what the adjustments are. The table has no legend, so it is unclear what statistical tests were used and what each column means. This is likely a raw output from a statistical software, which needs to be edited further for "human consumption". Please clean up this table so that all parts are understandable.
"I previously reviewed this paper previously in Research Hub and you can read these comments via the Research Hub review page here: https://www.researchhub.com/paper/9416337/cell-type-proportions-rather-than-dna-methylation-in-the-cord-blood-show-significant-associations-with-severe-preeclampsia/reviews
The original preprint linked to the Research Hub review is here: https://www.medrxiv.org/content/10.1101/2025.06.09.25329270v1"

Methods

Are the methods appropriate to the aims of the study, are they well described, and are necessary controls included? Choose an item.

Conclusions

Are the conclusions adequately supported by the data shown? Choose an item.

Reporting Standards

Does the manuscript adhere to the journal's guidelines on [minimum standards of reporting?] Choose an item.

Choose an item.

Statistics

Are you able to assess all statistics in the manuscript, including the appropriateness of statistical tests used? Choose an item.

Quality of Written English

Please indicate the quality of language in the manuscript: Choose an item.

Declaration of Competing Interests

Please complete a declaration of competing interests, considering the following questions:

- Have you in the past five years received reimbursements, fees, funding, or salary from an organisation that may in any way gain or lose financially from the publication of this manuscript, either now or in the future?
- Do you hold any stocks or shares in an organisation that may in any way gain or lose financially from the publication of this manuscript, either now or in the future?
- Do you hold or are you currently applying for any patents relating to the content of the manuscript?
- Have you received reimbursements, fees, funding, or salary from an organization that holds or has applied for patents relating to the content of the manuscript?
- Do you have any other financial competing interests?
- Do you have any non-financial competing interests in relation to this paper?

If you can answer no to all of the above, write 'I declare that I have no competing interests' below. If your reply is yes to any, please give details below.

I declare that I have no competing interests.

I agree to the open peer review policy of the journal. I understand that my name will be included on my report to the authors and, if the manuscript is accepted for publication, my named report including any attachments I upload will be posted on the website along with the authors' responses. I agree for my report to be made available under an Open Access Creative Commons CC-BY license (http://creativecommons.org/licenses/by/4.0/). I understand that any comments which I do not wish to be included in my named report can be included as confidential comments to the editors, which will not be published.

Choose an item.

To further support our reviewers, we have joined with Publons, where you can gain additional credit to further highlight your hard work (see: https://publons.com/journal/530/gigascience). On publication of this paper, your review will be automatically added to Publons, you can then choose whether or not to claim your Publons credit. I understand this statement.

Yes Choose an item.
