# Supplementary material for: Cord blood DNA methylation and cell-type composition are not significantly associated with severe preeclampsia after cell-type and clinical covariate adjustment
Source: Gigascience. 2026 Jan 16;15:giag002. doi: 10.1093/gigascience/giag002 (PMC13014471; doi:10.1093/gigascience/giag002)
Supplement: giag002_Reviewer_2_Report_original_submission — Karolis Koncevicius -- 7/15/2025 [file giag002_reviewer_2_report_original_submission.pdf]

Reviewer Report

Title: Cord Blood DNA methylation and Cell Type composition are not significantly associated with severe preeclampsia, after confounding adjustment

Version: Original Submission **Date:** 7/15/2025

Reviewer name: Karolis Koncevicius

Reviewer Comments to Author:

The authors investigate DNA methylation differences between preeclampsia cases and controls. The main finding of the study is that previously reported differences were likely caused by cell type heterogeneity and gestational age mismatch. Given what is known about the issues of cellular heterogeneity the finding is not surprising and can be considered to fall into the category of a "negative result". However, in my opinion findings like this provide a step forward in understanding the real relationships between epigenetics, cell type differences, and disease. As such this result should be relevant to anyone interested in DNA modification dynamics in preeclampsia and possibly the investigation of epigenetic differences in birth and pregnancy studies more generally.
I have some comments and proposed additions to the methods and results sections. However, in most cases these will be suggestions for improvement rather than hard flaws with the study.
Introduction:
The introduction seems clear and succinct. It introduces the problem, shows what has been done before, and highlights major finding disagreements between the results of the studies conducted so far, which clearly indicates that there is a problem. Then the authors propose a potential solution to this problem - performing EWAS while adjusting for cell type proportions and other necessary covariates, such as gestational age.
The only suggestion I have for introduction is to consider mentioning the (exact or approximate) sample sizes of each previous study. I was able to find this information in the next section - "materials and methods", and after seeing how small the sample sizes were it was less surprising that their results disagreed. Even the previous reported meta analysis (Kazmi et. al.) only had 135 cases, which would not be considered "large scale". This should be turned into another item highlighting the need of the present study - to integrate together all the previous studies conducted so far in order to boost sample size further.
Methods:
The methods section is written very clearly, it depicts what has been done both in terms of data preparation and subsequent data analysis. In addition there is a link to a GitHub repository containing the code. The data-preparation file contained in the GitHub repository follows the steps mentioned in the methods - even the removal of one outlier and imputation of missing BMI values. My only suggestion for the git repository would be to include a README file with some guidelines describing how to run the scripts on some actual datasets.
The section describing cohorts, sample preparation, and DNA methylation profiling are all excellent. It lists the sample sizes as well as GEO repository IDs for each dataset that is used in the study. The

methods for DNA extraction seem standard and DNA methylation profiling with arrays was performed by a dedicated facility. The only thing potentially missing here is to mention how the step of bisulphite conversion was done.

The data preparation steps can be improved.

First, it is not fully clear how the probes were filtered by detection p-values. The manuscript says that probes with detection p-value below 0.01 were removed. But the list of such probes will be different for each sample. Was the probe removed even if a single sample had a poor detection p-value? Or was the strategy the same as for low bead count (5% of samples had to have a low detection p-value)? In addition consider an alternative and more robust method for obtaining detection p-values (PMID 30678737)

It is unclear what "removing probes with no match CpG site" mean. Are these non-CG (CH) probes as well as control (rs) probes? If yes, in my opinion, that would be a clearer wording.

Removing cross-hybridizing probes (that map to multiple locations in the genome) is clear. But based on the reported number I think the annotation for which probes have cross-hybridization issues is probably outdated. Here I would recommend to use a modern and comprehensive annotation of probe issues compiled by Zhou.et.al (PMID 27924034, https://zwdzwd.github.io/InfiniumAnnotation).

The normalization was done with BMIQ, which adjusts the intensities between Type I and Type II probes. I think this is a good strategy but probably will not be that relevant for the results of the paper, as the analysis will not involve comparison between probes, but between samples within the same probe.

ComBat was used in order to deal with potential batch effects. I would recommend against using ComBat as it has been repeatedly reported to cause complex issues and should not be used without strict supervision (PMIDs 29616078 and 32605541, among others). Since batch effects are expected to manifest as mean-intensity differences between experiments and sentrix plates they can be controlled in the same way as cell type proportion effects - by including them in the model. Furthermore ComBat might interfere with cell type deconvolution, since the reference datasets used for deconvolution were likely not treated with ComBat.

Cell type deconvolution step is a bit unclear. The manuscript states that it used a reference by Gervin et. al. and in the software section it states that the R package EpiDISH was used to perform the deconvolution. However, as fas as I am aware, EpiDISH does not contain a reference from Gervin et. al. Here a clearer specification about how deconvolution was performed should be provided.

In addition, the newer version of EpiDISH has a "uni life" reference (centUniLife.m object) which contains 19 cell types (cord blood + 12 extended cell types, such as memory/naive stratification for T lymphocytes, DOI https://doi.org/10.1186/s13073-025-01489-7). It would be interesting to test if this reference would allow a more granular specification of cell type differences between PE and controls.

The strategy for selecting clinical confoudners raises a small concern. The effects were averaged across all investigated CpGs. However, if a confounder (such as BMI) has an influence on a small set of cytosines it will not be detected by such a global analysis and yet it would still confound those specific CpGs in site-by-site comparisons.

For EWAS the limma package is used in order to perform a modulated analysis which pools the variance estimates across many CpGs in order to increase statistical power. The corretion for multiple testing is also performed. I can see no issues with the EWAS step.

The meta analysis and combination of cohorts again uses ComBat in order to remove batch effect. As

mentioned previously even a single application of ComBat can cause issues, and if it is used again on top of datasets already pre-processed using ComBat it can only make things worse. Instead, I would simply inlcude a dummy variable for cohort in a regression model supplied to limma.

Including additional preterm samples as controls seems like a good idea. But it is uncler why there is an additional correction for null-distribution bias and inflation ("bacon") here, which was not used in any of the previous analyses.

For the detection of differentially methylated regions the authors used "bumphunter" which is a well known algorithm. The selected method has a lot of parameters, but none of them are listed here. It would be good to expand the description a bit and mention what parameters were used in order to detect DMR's. And if default settings were used, that could be specified as well.

Results:

The section about cell proportion variability across clinical variables is clear and well examined. One comment here is that it would be really interesting to check if UniLife reference might give additional insights.

It also seems relevant to check whether the same patterns of cell type proportion difference could be reproduced in other cohorts. Seems like this comparison was performed and described later (in the section talking about GA and cell type association). But I would consider merging it with this section for better structure and flow.

I would also recommend one additional step to make sure that cell type heterogeneity is the sole cause of PE DNA methylation differences. Currently a case can be made that a model with all the additional adjustments has 10+ more covariates when compared with a model that does not adjust for cell types and additional clinical variates. Given low cohort sample sizes a more complex model will be severely under-powered. Hence, it could be suspected that the lack of significant differences (after adjustment for multiple testing) can also be a side effect of low statistical power. In order to test this posibility I would recommend following a similar strategy that was done in a meta analysis of schizophrenia (PMID 33646943). Briefly, I suggest to investigate the cytosines reported as significantly associated with PE (when no adjustment for confounders is performed) and to compare their effect sizes (DNA methylation differences) and effect directions (hypo vs hyper methylated) before and after correction for cell type proportions. If the results were indeed caused by cell type heterogeneity this analysis will reveal that the effect sizes became a lot smaller and the directions of effects are no longer consistent with the original findings. However, if the effect direction (hypo vs hyper) after adjustments still correlates with original results then the problem of low power could be suspected as a culprit.

An additional analysis that would be really interesting to see is whether the original EWAS hits were not caused by a mismatch in GA. Since there is a dataset with pre-term and full-term samples it is possible to look for differentially methylated cytosines between pre- and full- without any adjustment for cell types. And then to compare whether those cytosines overlap with the cytosines reported to be different between PE and controls. This would strongly indicate that one of the causes of previous results is GA mismatch and make a strong case for taking GA into account when doing EWAS in any study related to pregnancy and birth.

Discussion:

The discussion is very professional and clear. It highlights the issue of cellular heterogeneity and lists examples of this issue in previous EWAS studies. It spends considerable amount of time talking about

potential limitations, which include small sample sizes and using computationally estimated cell type proportions.

One thing I would like to see added here is a discussion about more granular cell type decomposition and how that could play a role. For example, B lymphocytes might not show an effect between PE and controls, but the effect could still be present in the proportion of naive and mature B cells.

Another thing I would like to see added is a point about the broader context of the findings. The issues investigated by the authors are likely relevant not only to preeclampsia cases but to all epigenetic research conducted on cohorts related to pregnancy outcomes. I would like to see a cautionary note aiming at this broader target.

Other:

Some wording and sentences

1. "We validated this lack of CpG changes using multiple published cord blood methylation datasets." Should be more precise. These are not "CpG" changes but cytosine methylation changes.
2. "Cord blood consists of many diverse cell types, each with a distinct epigenome profile as defined." Unclear what the last two words "as defined" are trying to say. I think they should be removed?
3. SVD stands for "Singular Value Decomposition", not "Singular Vector Decomposition".

Some comments about the figures:

Figure 1. Study overview and experiment design.

All clear and nicely presented.

Figure 2. Cell types in samples.

The samples in heatmap are currently clustered by granulocyte count, which is not that interesting. Consider arranging the samples by diseases status instead.

Figure labels (in boxplots) have underscores in them.

Figure 3.

The title of this figure is a bit long, I would move the part about cohorts and figure letters into the caption text instead.

Some figure annotations contain underscores.

The "p-value" should not be capitalized (in volcano plots).

Figure 4. Cell type proportions in relationship with gestational age.

I would recommend showing the actual p-values instead of writing NS. Some readers might want to draw their own conclusions and be able to choose their own p-value cut-offs.

Figure 5. Cell proportions in relationship with gestational age are coherent in two different datasets

The term "P-interaction" is a bit awkward, consider using "interaction p-value" instead.

Supplementary Figure 1.

All clear.

Supplementary Figure 2.

Density plot B is said to be a result of density plot (A) after removing a single sample. However, for some reason, seems like more than one sample (density line) disappeared from A. Maybe more than one outlier was removed here?

Supplementary Figure 4.

"p-value" should not be capitalized.

Supplementary Figure 5.

Some underscores in figure labels, and CamelCase ("CellType") for boxplot.
Table 1.
All clear.
Supplementary Table 1.
All clear.
Supplementary Table 2.
All clear.
Good study, I had a good time while reading your manuscript and read it with interest. Hope this is helpful.
P.S. I previously reviewed this paper previously in Research Hub and you can read these comments via the Research Hub review page here: https://www.researchhub.com/paper/9416337/cell-type-proportions-rather-than-dna-methylation-in-the-cord-blood-show-significant-associations-with-severe-preeclampsia/reviews

Methods

Are the methods appropriate to the aims of the study, are they well described, and are necessary controls included? Choose an item.

Conclusions

Are the conclusions adequately supported by the data shown? Choose an item.

Reporting Standards

Does the manuscript adhere to the journal's guidelines on minimum standards of reporting? Choose an item.

Choose an item.

Statistics

Are you able to assess all statistics in the manuscript, including the appropriateness of statistical tests used? Choose an item.

Quality of Written English

Please indicate the quality of language in the manuscript: Choose an item.

Declaration of Competing Interests

Please complete a declaration of competing interests, considering the following questions:

- Have you in the past five years received reimbursements, fees, funding, or salary from an organisation that may in any way gain or lose financially from the publication of this manuscript, either now or in the future?
- Do you hold any stocks or shares in an organisation that may in any way gain or lose financially from the publication of this manuscript, either now or in the future?
- Do you hold or are you currently applying for any patents relating to the content of the manuscript?
- Have you received reimbursements, fees, funding, or salary from an organization that holds or has applied for patents relating to the content of the manuscript?
- Do you have any other financial competing interests?
- Do you have any non-financial competing interests in relation to this paper?

If you can answer no to all of the above, write 'I declare that I have no competing interests' below. If your reply is yes to any, please give details below.

I declare that I have no competing interests

I agree to the open peer review policy of the journal. I understand that my name will be included on my report to the authors and, if the manuscript is accepted for publication, my named report including any attachments I upload will be posted on the website along with the authors' responses. I agree for my report to be made available under an Open Access Creative Commons CC-BY license (http://creativecommons.org/licenses/by/4.0/). I understand that any comments which I do not wish to be included in my named report can be included as confidential comments to the editors, which will not be published.

Choose an item.

To further support our reviewers, we have joined with Publons, where you can gain additional credit to further highlight your hard work (see: https://publons.com/journal/530/gigascience). On publication of this paper, your review will be automatically added to Publons, you can then choose whether or not to claim your Publons credit. I understand this statement.

Yes Choose an item.
