# Supplementary material for: Cord blood DNA methylation and cell-type composition are not significantly associated with severe preeclampsia after cell-type and clinical covariate adjustment
Source: Gigascience. 2026 Jan 16;15:giag002. doi: 10.1093/gigascience/giag002 (PMC13014471; doi:10.1093/gigascience/giag002)
Supplement: giag002_Reviewer_3_Report_original_submission — Mariona Bustamante -- 7/16/2025 [file giag002_reviewer_3_report_original_submission.pdf]

Reviewer Report

Title: Cord Blood DNA methylation and Cell Type composition are not significantly associated with severe preeclampsia, after confounding adjustment

Version: Original Submission **Date:** 7/16/2025

Reviewer name: Mariona Bustamante

Reviewer Comments to Author:

Summary
This study investigates the association between cord blood DNA methylation and pre-eclampsia using both original data and several public datasets. The findings suggest that differences in DNA methylation between cases and controls are largely attributable to variations in cell type proportions, which are, in part, explained by the shorter gestational age observed in pre-eclampsia cases.
Comments
1. Main comments
- The authors tested associations between estimated cell type proportions and case/control status or gestational age using linear regression. However, since cell type proportions are compositional in nature, I recommend reanalyzing these associations using statistical methods specifically designed for compositional data (e.g., log-ratio transformations or compositional regression models).
- To explore DNA methylation changes within specific cell types in relation to pre-eclampsia, it would be valuable to perform an epigenome-wide association study (EWAS) that includes an interaction term between case/control status and cell type proportions, as demonstrated in this study: https://www.nature.com/articles/s41467-020-18618-y.
- Could the authors estimate the proportion of the association between pre-eclampsia and cell type proportions that is mediated by gestational age versus the proportion that is directly attributable to pre-eclampsia? A formal mediation analysis could help disentangle these effects and clarify the underlying mechanisms.
2. Abstract
- "Previous epigenome-wide association studies (EWAS) on PE have produced inconsistent results, possibly due to inadequate adjustment for confounders." In which tissue? Cord blood? Placenta?
- Authors should add sample size of the own and public data analyzed in the study.
- "We validated this lack of CpG changes using multiple published cord blood methylation datasets." Authors should change to "CpG DNA methylation changes". Moreover, in previous sentences authors already say this "disappeared across our data, the two public datasets, and the meta-analysis.". Isn't it redundant?
- In the summary, authors should state clear that they talk about cord blood and not other tissues.
3. Methods
- The datasets analysed in the manuscript use different pre-processing pipelines. While this is fine for cases and controls processed within the study, it could introduce technical noise when using cases and controls from different studies. In particular when using the idiopathic preterm babies from one study as

controls in another study. I would suggest to pre-process these controls following the same pipeline as for the cases.
- In the section "Cell-type deconvolution in umbilical cord whole blood (CB)", authors should clarify if this was done for all the datasets, including public datasets, or just for their samples.
- Authors should indicate the type of meta-analysis done (fixed effects? Weighted-variance?).
- Authors should give more details about this point "To harmonize the datasets, we applied the combat function to remove batch effects while preserving sample group information."
- "Lastly, we calculated differentially methylated probes again, with adjustment to the sample group and estimated cell types and plotted another volcano plot." What does "adjustment to sample group" mean?
- "We did not include data from Kashimi et al. because there was no significant cpg before the confounder adjustment." I think lack of cohort individual associations is not a reason to exclude the study from the meta-analysis and I would suggest the authors to include it.
- Why is the model of idiopathic preterm controls corrected using "bacon" but not the others? It sounds inconsistent. I would suggest to apply bacon or not to all the models.
- For the DMR I would suggest to use more than one tool and keep the overlap as final results as there are considerable differences between methods.
4. Results
- I would delete some of the text referring to methods from the results section. (ie. I would delete lines 263-270, and 273-275, etc…).
- In Figure 1 add "gene-based EWAS" and "DMR" analyses
5. Discussion
- "We did not detect significant differences in the inferred proportion of endothelial cells between the control and PE groups using the DNA methylation data from paired placental samples." This sentence requires a reference.
Minor comments
1. Define variables the first time they appear (ie. BMI in the abstract)
2. Correct typos (ie. Preeclampsia(PE) - space missing)
3. Human gene names in cursive (ie. AVPR1a, OXTR, and PKCB)
4. Change "didn't" to "did not".
5. Correct: "... (GSE103253). and (3)..."
6. Correct: "...cpg..."
7. Keep naming of "combat" consistent along the text.
8. In the sentence "... related to Rheumatoid Arthritis after...", change disease name to non-capital letters.
9. Change "Causcasian" for "European origin / ancestry".

Methods

Are the methods appropriate to the aims of the study, are they well described, and are necessary controls included? Choose an item.

Conclusions

Are the conclusions adequately supported by the data shown? Choose an item.

Reporting Standards

Does the manuscript adhere to the journal's guidelines on minimum standards of reporting? Choose an item.

Choose an item.

Statistics

Are you able to assess all statistics in the manuscript, including the appropriateness of statistical tests used? Choose an item.

Quality of Written English

Please indicate the quality of language in the manuscript: Choose an item.

Declaration of Competing Interests

Please complete a declaration of competing interests, considering the following questions:

- Have you in the past five years received reimbursements, fees, funding, or salary from an organisation that may in any way gain or lose financially from the publication of this manuscript, either now or in the future?
- Do you hold any stocks or shares in an organisation that may in any way gain or lose financially from the publication of this manuscript, either now or in the future?
- Do you hold or are you currently applying for any patents relating to the content of the manuscript?
- Have you received reimbursements, fees, funding, or salary from an organization that holds or has applied for patents relating to the content of the manuscript?
- Do you have any other financial competing interests?
- Do you have any non-financial competing interests in relation to this paper?

If you can answer no to all of the above, write 'I declare that I have no competing interests' below. If your reply is yes to any, please give details below.

None

I agree to the open peer review policy of the journal. I understand that my name will be included on my report to the authors and, if the manuscript is accepted for publication, my named report including any attachments I upload will be posted on the website along with the authors' responses. I agree for my report to be made available under an Open Access Creative Commons CC-BY license (http://creativecommons.org/licenses/by/4.0/). I understand that any comments which I do not wish to

be included in my named report can be included as confidential comments to the editors, which will not be published.

Choose an item.

To further support our reviewers, we have joined with Publons, where you can gain additional credit to further highlight your hard work (see: https://publons.com/journal/530/gigascience). On publication of this paper, your review will be automatically added to Publons, you can then choose whether or not to claim your Publons credit. I understand this statement.

Yes Choose an item.
